# Deprotective Lossen rearrangement: a direct and general transformation of Nms-amides to unsymmetrical ureas†

**DOI:** 10.1039/d4sc04974h

**Published:** 2024-09-10

**Authors:** Philipp Spieß, Jakub Brześkiewicz, Nuno Maulide

**Affiliations:** a Institute of Organic Chemistry, University of Vienna Währinger Strasse 38 1090 Vienna Austria nuno.maulide@univie.ac.at

## Abstract

Ureas stand out as potent pharmacophores in drug development, rendering them a prime focus for synthesis. Herein, we present an appealing entry point for urea synthesis from protected amines (Nms-amides) and relying on a Lossen-type rearrangement process as an elegant example of deprotective functionalisation. The method developed exhibits an exceptionally broad tolerance towards various protected amines, encompassing numerous drug derivatives, and delivers high reaction yields.

## Abstract 2

Herein, we report the development of a deprotective functionalisation reaction that allows for the synthesis of unsymmetrical ureas through a Lossen-type rearrangement of Nms-protected amines.

## Graphical abstract

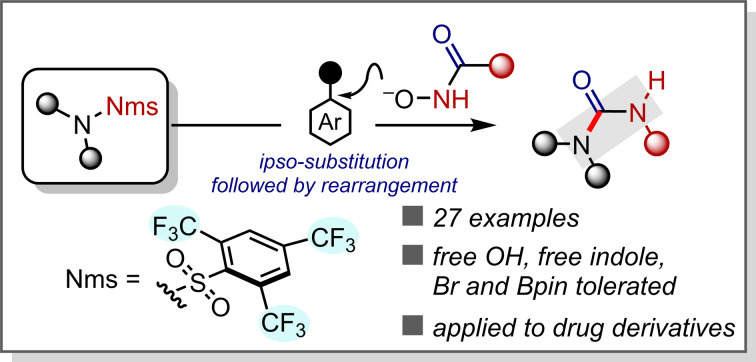

## Introduction

**Scheme 1:**
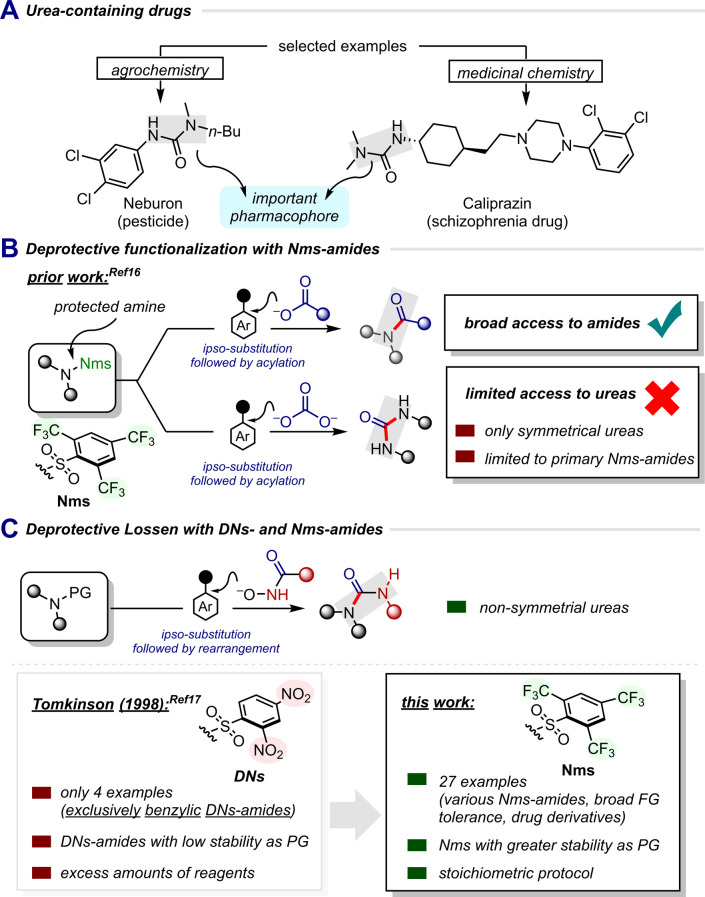
Importance of ureas, previous work on deprotective functionalisation and this work on a deprotective Lossen rearrangement.

The inception of organic chemistry is widely considered to be marked by the landmark synthesis of urea,^1^ and ureas have shortly thereafter become pivotal entities in drug development and medicinal chemistry.^2^ The versatile urea functionality enables the modulation of drug efficacy and constitutes an important pharmacophore in FDA-approved drugs (Scheme 1A).^3–7^ As a result, new strategies for the synthesis of ureas, particularly unsymmetrical ones, are constantly being sought, striving in particular to avoid commonly employed toxic reagents (*e.g.*, phosgene).^8–14^

Protecting groups, especially for amines—typically aimed at temporarily downregulating their basicity or nucleophilicity—have long been integral to organic synthesis. However, despite their utility, protecting groups are increasingly viewed unfavourably due to their tendency to increase step count in given synthetic sequences.

Our group recently introduced a synthetic concept termed “deprotective functionalisation” focused on Nms-amides,^15^ with the aim of circumventing the traditional approach of initial deprotection followed by a separate functionalisation, as two discrete steps.^16^ This concept revolves around the notion of simultaneous deprotection and functionalisation in a single step (rather than two telescoped operations) and leverages the innate reactivity of the Nms group. In our recent study, we presented the direct conversion of Nms-amides into carboxamides (using carboxylates as nucleophiles) or symmetrical ureas (using carbonates) (Scheme 1B).^16^

Although the reaction scope for amides exceeded our expectations, the synthesis of ureas notably remained limited, as only primary sulphonamides proved effective as starting materials, and the restriction to symmetric products was a notorious limitation. Yet, considering the prevalence of ureas in drugs, the prospect of a universal method for non-symmetrical urea synthesis *via* “deprotective functionalisation” remained enticing. Our attention was drawn to a seminal work by Tomkinson and coworkers, which showed the ability of 2,4-dinitrobenzenesulphonamides (DNs) to engage hydroxamic acids, resulting in the formation of non-symmetrical ureas (Scheme 1C left).^17^ Although these reactions were efficient, the range of substrates was limited to four single examples, with only benzylic dinitrobenzenesulphonamides shown to undergo the transformation. Moreover, using DNs-amides in multi-step syntheses poses significant challenges due to their instability towards organometallic reagents, reductions and even mildly basic conditions (*e.g.* NEt_3_), sometimes even restricting their installation step.^18^ This overall instability makes them often unsuitable for protecting amines, especially in longer synthesis sequences. In addition, this earlier work used an excess of both hydroxamic acid and base, which makes it less favourable from an economic point of view.

In light of Tomkinson's report,^17^ we hypothesised that Nms-amides, which have been shown to possess greatly enhanced stability relative to nitrobenzenesulfonamides,^15^ could serve as an efficacious solution if they are capable of reacting with hydroxamic acids. This could overcome the inherent limitations of DNs-sulphonamides, allowing the full potential of deprotective functionalisation to be exploited.

Herein, we report the successful implementation of mild and economical reaction conditions for the conversion of Nms-amides into ureas in a single step, thus unfolding a truly compatible deprotective functionalisation method for the synthesis of non-symmetrical ureas (Scheme 1C right).

## Results and discussion

**Scheme 2:**
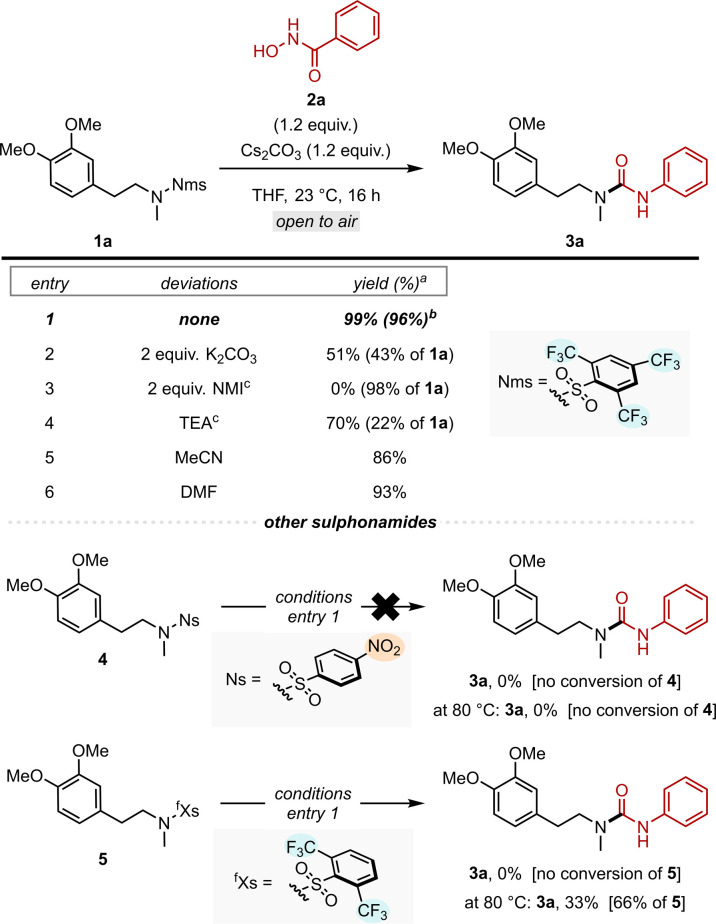
Optimisation studies with Nms-amides and other sulphonamides. All reactions were performed on a 0.1 mmol scale. ^*a*^NMR yield using CH_2_Br_2_ as internal standard. ^*b*^Isolated yield. ^*c*^2 equivalents of 2a were used. NMI = *N*-methylimidazole.

We set out to investigate the reaction between Nms-amide 1a and commercially available benzhydroxamic acid (2a). After a series of optimisation reactions, we found the conditions detailed in Scheme 2 to be optimal, enabling the isolation of the desired urea product 3a in an excellent yield of 96%. Notably, this reaction can be carried out at room temperature without any precautions to ensure either an inert atmosphere or the strict absence water. These reaction conditions differ from Tomkinson's earlier work in that the equivalents of base and hydroxamic acid used were greatly reduced (from 2.0 equivalents to now 1.2 equivalents).^17^ In addition, THF was used as the reaction solvent instead of DMF.

Our optimisation showed the choice of base to be crucial to ensure high conversions (entries 2–4). While triethylamine and K_2_CO_3_ still afforded the desired urea in moderate to good yields (with unreacted 1a constituting the remainder of the mass balance, entries 2, 4), *N*-methylimidazole (NMI) allowed no conversion of the substrate (entry 3). While a range of non-aprotic solvents gave the desired product in comparably good yields (entries 5–6), THF remains the best performing reaction solvent. While this deprotective functionalisation reaction proceeded readily with Nms-amides, we became intrigued by the prospect of testing other widespread sulphonamides.^19–21^ However, neither Ns-amide (4) nor ^f^Xs-amide (5) gave the desired urea product 3a under our optimised reaction conditions at ambient temperature, all starting sulfonamide remained unreacted (Scheme 2 bottom). Consequently, we hypothesised that heating might have a beneficial effect on the conversion of 4 and 5. Indeed, at a temperature of 80 °C, the ^f^Xs-amide (5) exhibited a low level of conversion (33%), while the Ns-amide (4) still remained entirely unreacted. It is conceivable that raising the temperature even further could boost the conversion of ^f^Xs-amide, but doing so in THF would pose additional challenges. However, what these studies unmistakably demonstrate is the significant variability in reactivity among the series: Nms, ^f^Xs, Ns. This variability showcases the delicate balance that electron density imparts to this reaction.

**Scheme 3:**
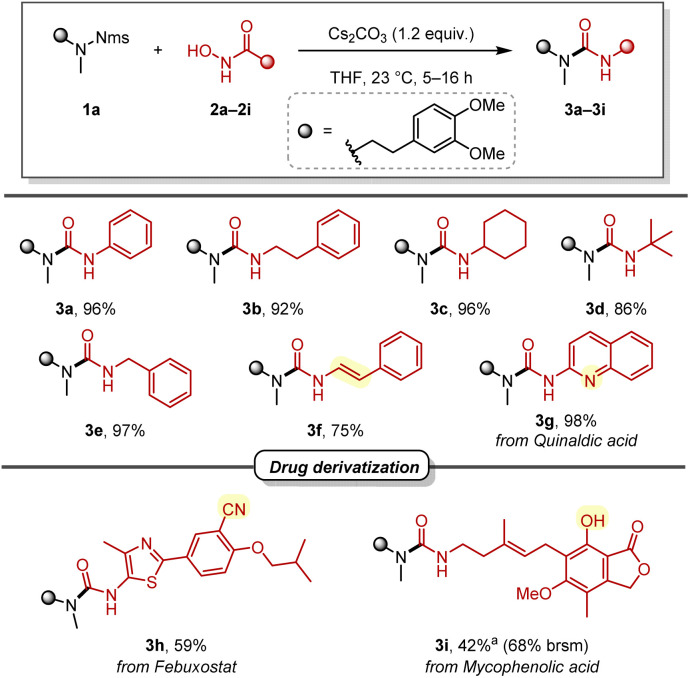
Scope of hydroxamic acids. ^*a*^1.5 equiv. of hydroxamic acid and Cs_2_CO_3_ were used.

With optimised conditions in hand, we turned to explore the scope of hydroxamic acids (Scheme 3). Alkyl, benzyl, and alkenyl hydroxamic acids were all successfully applied to this deprotective urea synthesis, providing the desired ureas (3a–3f) in 75–97% yield. Notably, with 3f, an enamide (a moiety renowned for its versatility as a building block in organic synthesis^22–24^) was directly accessed through the rearrangement of a cinnamic acid derivative. In addition, a heterocyclic hydroxamic acid derived from quinaldic acid provided the desired urea in excellent yield (3g). Looking at more complex structures, a derivative of febuxostat, a drug for the treatment of gout, and mycophenolic acid, an immunosuppressant, were also synthesised in acceptable yields (3h and 3i). It is noteworthy that the free phenol functionality of mycophenolic acid (3i) was tolerated without the need for a protecting group.

**Scheme 4:**
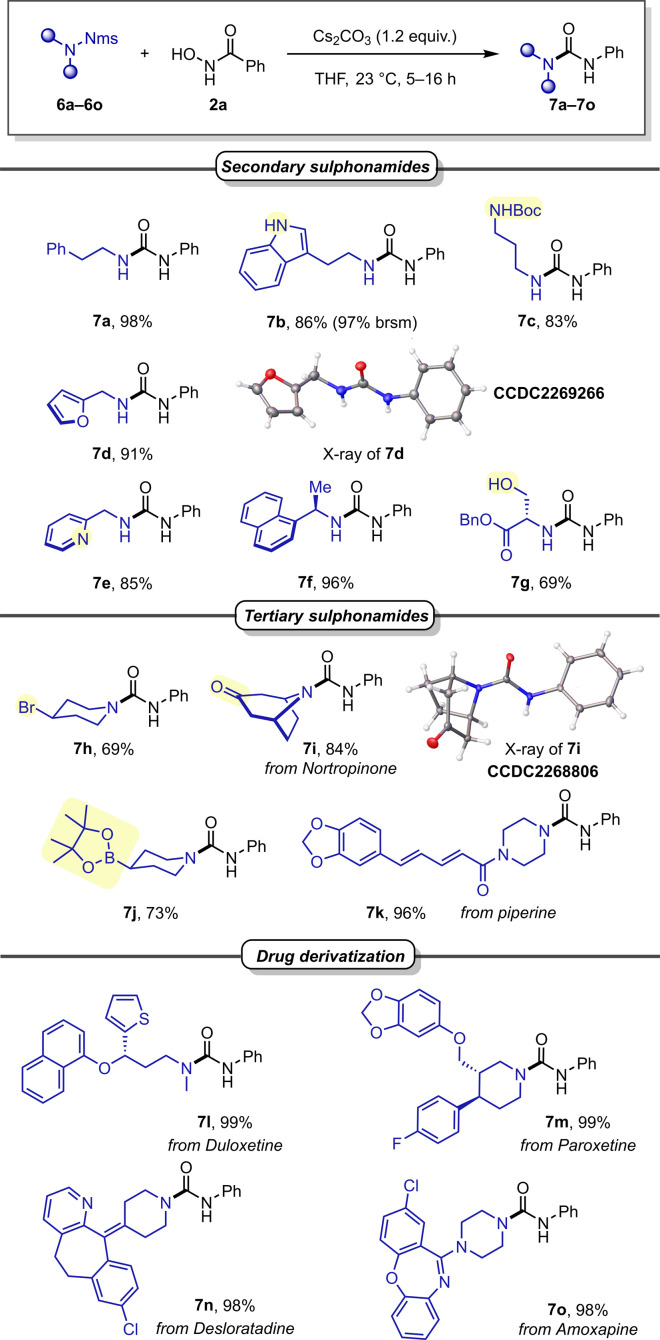
Scope of sulphonamides.

We subsequently investigated different Nms-amides (Scheme 4) and were pleased to find that various secondary sulphonamides readily provided the desired urea products without adjustment of the reaction conditions (7a–7c). It is noteworthy that other nitrogen-containing functionalities, such as Boc-protected amine and a free indole, were well accommodated and provided the desired products in good reaction yields (7b, 7c). Product 7c merits special mention, as it features a diamine showcasing the orthogonality of Nms and Boc in the context of deprotective functionalisation.

Then, benzylic amines were investigated, all of which gave excellent yields, including such containing heterocyclic moieties such as furan (7d) or pyridine (7e), as well as a substrate with increased steric hindrance (7f). Encouraged by these promising outcomes, we were eager to examine Nms-protected serine, carrying a free hydroxyl group. In the event, it readily furnished the desired urea product in good yield (7g). Sensitive functionalities like Bpin or a secondary alkylbromide exhibited good tolerance under the basic conditions (7j and 7h). Similarly, a ketone (7i) and an unsaturated carboxamide (7k) were also well accommodated functionalities.

Encouraged by these findings, we shifted our focus to more intricate sulphonamide structures in drug derivatives. A range of blockbuster drugs, including Duloxetine (7l), Paroxetine (7m), Desloratadine (7n), and Amoxapine (7o), produced the desired ureas in near quantitative yields. This underscores the method's remarkable suitability for unlocking potential new drug derivatives in medicinal chemistry with ease.

**Scheme 5:**
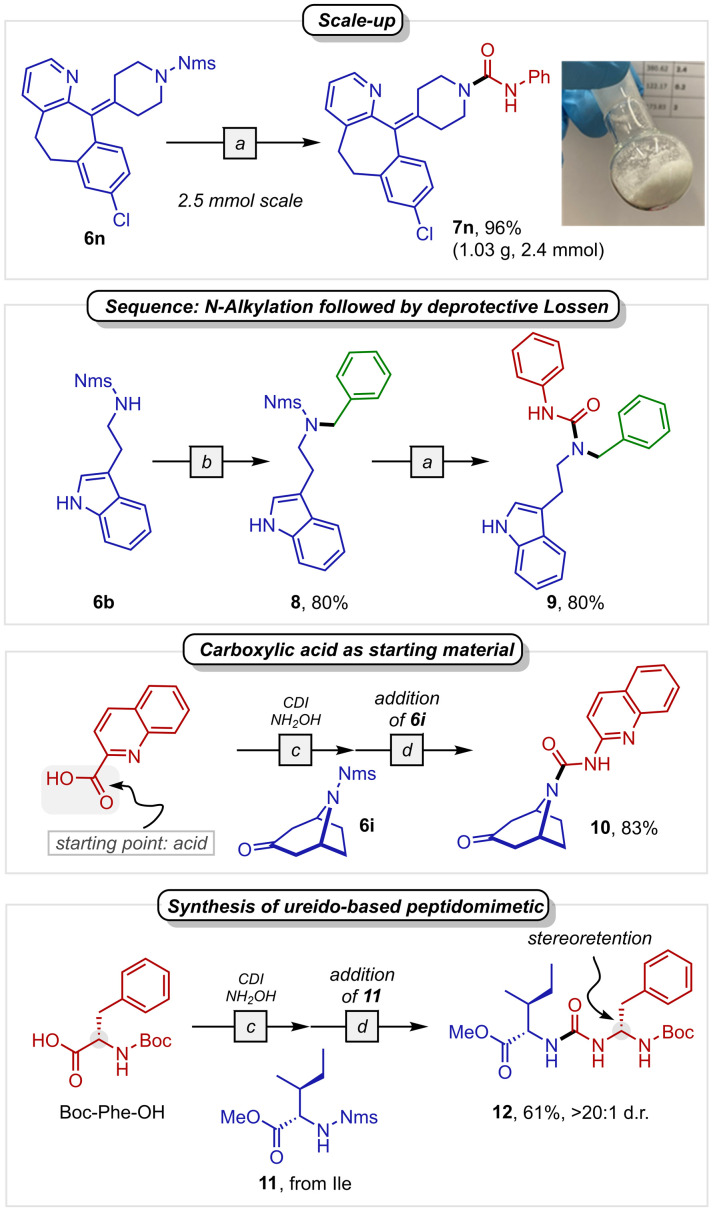
Application. ^*a*^See conditions from Scheme 2 entry 1. ^*b*^ Benzyl bromide (5.0 equiv.), K_2_CO_3_ (5.0 equiv.), acetone, 23 °C. ^*c*^Acid (1.5 equiv.), CDI (1.8 equiv.), MeCN, 1 h, then NH_2_OH·HCl (2.0 equiv.), 2 h. ^*d*^Addition of sulphonamide 6i or 11 (1.0 equiv.) and Cs_2_CO_3_ (4.0 equiv.).

The deprotective functionalisation of Desloratadine was performed on a gram-scale with comparable yields (Scheme 5, large scale *vs.* 98%, small scale). Following this, we aimed to assess the Nms-amide within a reaction sequence to exploit its capability for both *N*-alkylation as well as deprotective functionalisation. As shown (Scheme 5, middle), *N*-alkylation of Nms-amide 6b can be followed by its conversion to the corresponding indole urea 9 through deprotective Lossen rearrangement.

Finally, we aimed at streamlining the reaction by eliminating the need to separately prepare the hydroxamic acid before adding it to the sulphonamide. By generating the hydroxamic acid *in situ* (CDI + hydroxylamine),^12^ one enables the simple addition of sulphonamide 6i and Cs_2_CO_3_ to successfully deliver urea 10 in an excellent yield of 83%. This same approach was also utilised in synthesising a dipeptidomimetic (12), starting from Boc-Phe-OH and Nms-protected isoleucine (11) (Scheme 5, bottom). Notably, stereoretention was observed on the phenylalanine moiety. These results collectively highlight the simplicity and versatility of the chemistry described in this work.

**Scheme 6:**
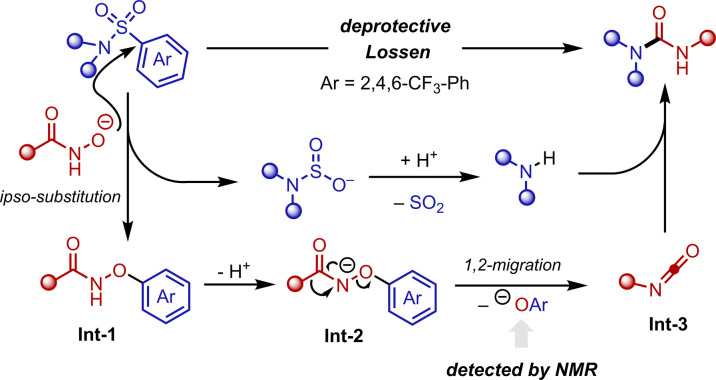
Proposed mechanism.

A plausible deprotective functionalisation mechanism can be derived from literature^13^ and our observations (Scheme 6): the *in situ* generated hydroxamate anion can accomplish *ipso*-attack to the aryl moiety of the Nms-amide.^25^ The thus formed Meisenheimer complex collapses and a hydroxamic ester (Int-1) is formed. Deprotonation of Int-1 forms Int-2, which undergoes 1,2-migration to form isocyanate (Int-3), releasing ArO^−^, whose presence was also confirmed after post-reaction.^26^ Ultimately, the free amine and the isocyanate (Int-3) recombine to form the observed urea.

## Conclusions

In summary, we have developed a mild reaction protocol capable of converting Nms-amides into ureas through the reaction with hydroxamic acids. The reaction exhibits remarkable versatility in accommodating various functionalities, thereby establishing itself as a dependable approach for effectively modifying complex protected amines with consistently high yields. Our investigations have also underscored that other sulphonamide protecting groups (Ns/^f^Xs) lack comparable reactivity. The method's remarkable scalability, in conjunction with its integration with preceding *N*-alkylation steps or the *in situ* preparation of hydroxamic acids, renders it a compelling option for more intricate urea synthesis.

## Data availability

All experimental data, and detailed experimental procedures are available in the published article and ESI.†

## Author contributions

The work was conceptualised by N. M. The experiments were performed by P. S and J. B. The manuscript was written through contributions of all authors. P. S and N. M. were involved in manuscript editing, finalising and overall supervision of the project. N. M. secured funding and supervised the entire work.

## Conflicts of interest

There are no conflicts to declare.

## Supplementary Material

SC-OLF-D4SC04974H-s001

SC-OLF-D4SC04974H-s002
