# Supplementary material for: Deprotective Lossen rearrangement: a direct and general transformation of Nms-amides to unsymmetrical ureas
Source: Chem Sci. 2024 Sep 10;15(38):15799–803. doi: 10.1039/d4sc04974h (PMC11385062; doi:10.1039/d4sc04974h)
Supplement: SC-015-D4SC04974H-s001 [file SC-015-D4SC04974H-s001.pdf]

Supplementary Information (SI) for Chemical Science.
This journal is © The Royal Society of Chemistry 2024

Deprotective Lossen Rearrangement: A Direct and General Transformation of Nms-Amides to Unsymmetrical Ureas

Philipp Spieß[a], Jakub Brześkiewicz[a], Nuno Maulide[a]*

[a]Institute of Organic Chemistry, University of Vienna, Währinger Straße 38, 1090 Vienna (Austria)
E-Mail: nuno.maulide@univie.ac.at, Homepage: http://maulide.univie.ac.at

Contents

1. General Information 2
2. Preparation of NmsCl and Nms-Amides 3
3. Reaction Optimization 6
4. Test of Various Sulfonamides 7
5. Experimental 8
   5.1. General Procedures 8
   5.2. Characterization of Hydroxamic Acids 9
   5.3. Characterization of Ureas 12
   5.4. Unsuccessful Substrates 25
   5.5. Scale-up Procedure 26
   5.6. One-pot Urea Synthesis 27
   5.7. Synthesis of ureido-based peptidomimetic 28
6. X-ray Crystallographic Data 29
7. NMR Spectra 31
8. References 62

1. General Information

Unless otherwise stated, all glassware was flame-dried before use and all reactions were performed under an atmosphere of argon. All solvents were distilled from appropriate drying agents prior to use. All reagents were used as received from commercial suppliers unless otherwise stated. Reaction progress was monitored by thin layer chromatography (TLC) performed on aluminum plates coated with silica gel F254 with 0.2 mm thickness. Chromatograms were visualized by fluorescence quenching with UV light at 254 nm or by staining using potassium permanganate. Flash column chromatography was performed using silica gel 60 (230-400 mesh, Merck and co.). Neat infrared spectra were recorded using a Perkin-Elmer Spectrum 100 FT-IR spectrometer. Wavenumbers (ν_{max}) are reported in cm^{-1}. Mass spectra were obtained using a Finnigan MAT 8200 or (70 eV) or an Agilent 5973 (70 eV) spectrometer, using electrospray ionization (ESI). All ^{1}H NMR and ^{13}C NMR spectra were recorded using a Bruker AV-400 or AV-600 spectrometer at 300K. Chemical shifts are given in parts per million (ppm, δ), referenced to the solvent peak of $CDCl_3$ defined at δ = 7.26 ppm (^{1}H-NMR) and δ = 77.2 (^{13}C-NMR), DMSO-d_6 defined at δ = 2.50 ppm (^{1}H-NMR) and δ = 39.5 (^{13}C-NMR), CD_3OD defined at δ = 3.31 ppm (^{1}H-NMR) and δ = 49.0 (^{13}C-NMR) or aetone-d_6 defined at δ = 2.05 ppm (^{1}H-NMR) and δ = 206.3, 29.8 (^{13}C-NMR). Coupling constants are quoted in Hz (*J*). ^{1}H-NMR splitting patterns are designated as singlet (s), doublet (d), triplet (t), quartet (q), heptet (hept), as they appeared in the spectrum. If the appearance of a signal differs from the expected splitting pattern, the observed pattern is designated as apparent (app). Splitting patterns that could not be interpreted or easily visualized are designated as multiplet (m) or broad (br).

2. Preparation of NmsCl and Nms-Amides

2,4,6-Tris(trifluoromethyl)benzenesulfonyl chloride (NmsCl)

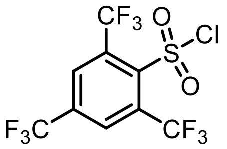

2,4,6-Tris(trifluoromethyl)sulfonyl chloride (NmsCl) was prepared according to a procedure reported in literature.[1] **^{1}H NMR (400 MHz, $CDCl_3$):** δ 8.43 (s, 2H) ppm. **^{19}F NMR (377 MHz, $CDCl_3$):** δ -54.3 (6F), -63.7 (3F) ppm. **^{13}C NMR (101 MHz, $CDCl_3$):** δ 145.5, 137.1 (q, *J* = 35.8 Hz), 133.6 (q, *J* = 35.3 Hz), 130.2 (m), 121.6 (q, *J* = 276.9 Hz) ppm.

Guide for the preparation of Nms-protected amines:

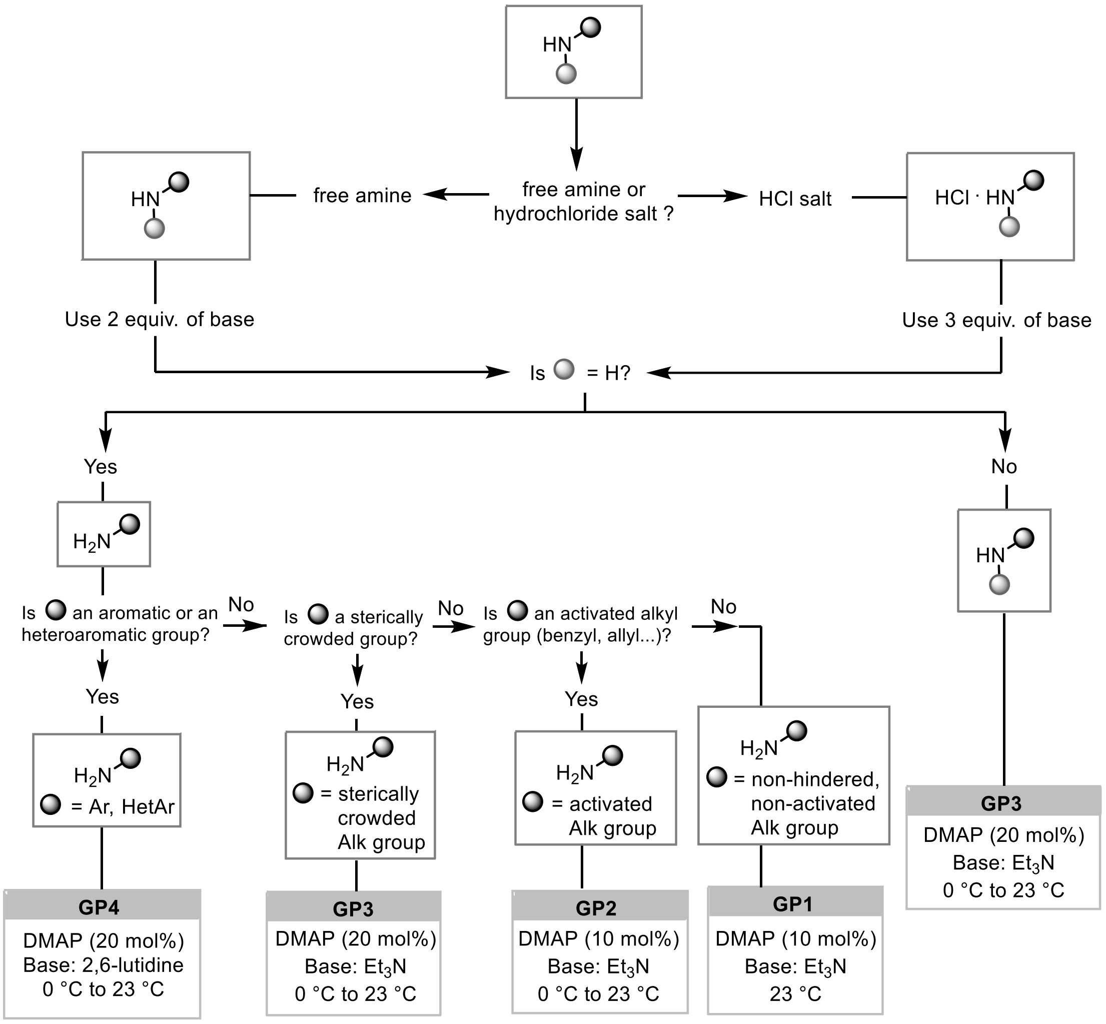

General Procedure 1 (GP1, Protection of non-hindered primary amines):

The corresponding amine (1.0 equiv.) was dissolved in DCM (0.1M) at room temperature (18-25 °C). DMAP (10 mol%), Et_3N (2.0 equiv. for free amines, 3.0 equiv for hydrochloride salts of amines) and NmsCl (1.2 equiv.) were added sequentially to the solution at the same temperature. The mixture was stirred at room temperature for 3-5 h. The progress of the reaction was monitored by TLC analysis. Then, the solvents were removed under vacuum. The residue was purified by column chromatography (silica gel, heptane:EtOAc).

General Procedure 2 (GP2, Protection of benzylamines):

The corresponding amine (1.0 equiv.) was dissolved in DCM (0.1M) at room temperature (18-25 °C). DMAP (10 mol%), Et_3N (2.0 equiv. for free amines, 3.0 equiv. for hydrochloride salts of amines) were added at this temperature. Then, the mixture was cooled to 0 °C and NmsCl (1.2 equiv.) was added in one portion. The reaction was allowed to slowly warm up to room temperature over 15 h (without removing the cooling bath after the addition of NmsCl). The progress of the reaction was monitored by TLC analysis. Then, the solvents were removed under vacuum. The residue was purified by column chromatography (silica gel, heptane:EtOAc).

General Procedure 3 (GP3, Protection of hindered primary amines and secondary amines):

The corresponding amine (1.0 equiv.) was dissolved in DCM (0.1M) at room temperature (18-25 °C). DMAP (20 mol%) and Et_3N (2.0 equiv. for free amines, 3.0 equiv for hydrochloride salts of amines) were added at this temperature. Then, the mixture was cooled to 0 °C and the arenesulfonyl chloride (1.2 equiv.) was added in one portion. The reaction was allowed to slowly warm up to room temperature over 14-20 h (without removing the cooling bath right after the addition of NmsCl). The progress of the reaction was monitored by TLC analysis. Then, the solvents were removed under vacuum. The residue was purified by column chromatography (silica gel, heptane:EtOAc).

General Procedure 4 (GP4, Protection of anilines):

The corresponding amine (1.0 equiv.) was dissolved in DCM (0.1M) at room temperature (18-25 °C). DMAP (20 mol%) and 2,6-lutidine (2.0 equiv.) were added at this temperature. Then, the mixture was cooled to 0 °C and the arenesulfonyl chloride (1.2 equiv.) was added in one portion. The reaction was allowed to slowly warm up to room temperature over 14-20 h (without removing the cooling bath right after the addition of NmsCl). The progress of the reaction was monitored by TLC analysis. Then, the solvents were removed under vacuum. Then, the solvents were removed under vacuum. The residue was purified by column chromatography (silica gel, heptane:EtOAc).

The Nms-amides (**1a**, **6a-6o**) used in this work were previously prepared by our group from the corresponding amines under the reaction conditions described above.[2,3]

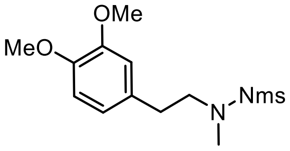

1aRef2

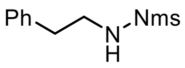

6aRef3

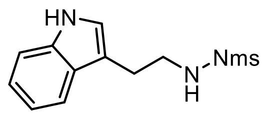

6bRef2

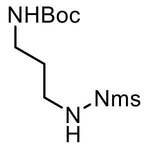

6cRef2

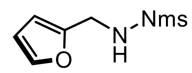

6dRef2

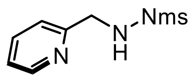

6eRef2

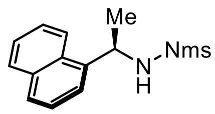

6fRef2

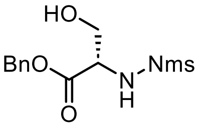

6gRef2

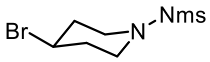

6hRef2

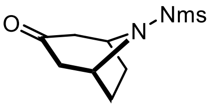

6iRef2

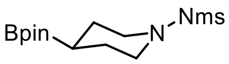

6jRef2

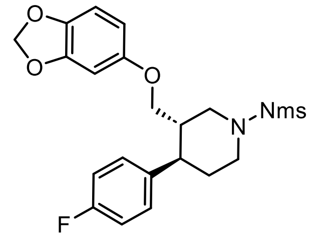

6mRef2

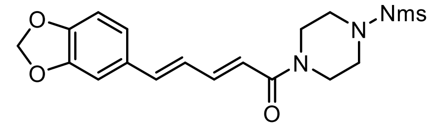

6kRef3

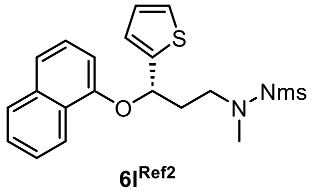

6lRef2

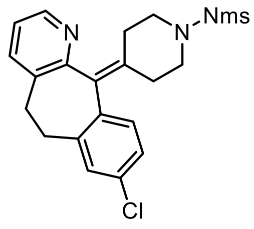

6nRef2

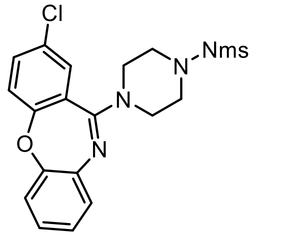

6oRef2

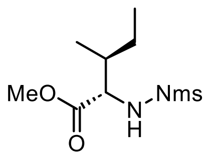

11 Ref2

3. Reaction Optimization

General procedure: A vial was filled with sulfonamide **1** (53.9 mg, 0.1 mmol, 1.0 equiv.) and benzhydroxamic acid (**2a**). The base was added, followed by solvent (0.1 M) and the mixture was stirred for 16 h at room temperature. The crude material was passed through a pad of Celite using EtOAc as eluent. The yield was determined by ^{1}H-NMR analysis using dibromomethane (7.0 µL, 17.4 mg, 0.1 mmol) as internal standard.

Table 1. Base optimization[a]

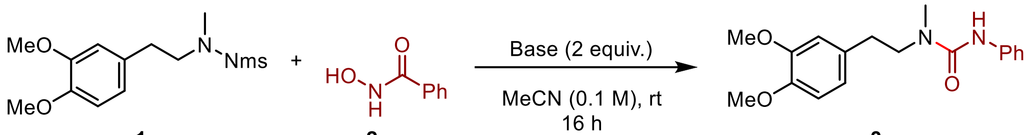

Entry	Base	Yield (%)[b, c]
1	Cs_2CO_3	**92**
2	$CsHCO_3$	**77** (23)
3	K_2CO_3	**51** (43)
4	N-Methylimidazole	**0** (98)
5	TEA	**70** (22)

[a] **1** (0.1 mmol), benzhydroxamic acid (0.2 mmol), base (0.2 mmol), MeCN (1 mL), rt, 16 h, air atmosphere. [b] NMR yield. [c] Yield of unreacted starting sulfonamide in parenthesis. TEA = triethylamine.

Table 2. Solvent optimization and other deviations[a]

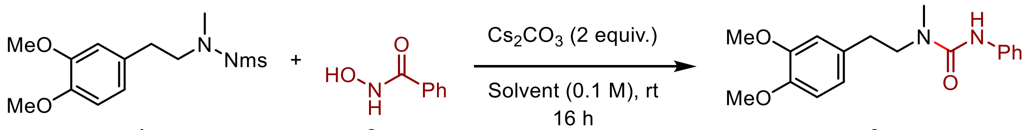

Entry	Solvent	Other deviations	Yield (%)[b]
1	DMF	-	**93**[c, d]
2	MeCN	1.2 equiv. of hydroxamic acid	**86**
3	THF	-	**99**
4	THF	1.2 equiv. of hydroxamic acid	**99**
**5**	**THF**	**1.2 equiv. of hydroxamic acid and 1.2 equiv. of Cs_2CO3**	**99 (96)**[c]

[a] **1c** (0.1 mmol), benzhydroxamic acid (0.2 mmol), Cs_2CO_3 (0.2 mmol), solvent (1 mL), rt, 16 h, air atmosphere. [b] NMR yield. [c] Isolated yield. [d] Reaction performed under argon.

4. Test of Various Sulfonamides

General procedure: A vial was filled with sulfonamide **1a**, **4** or **5** (0.1 mmol, 1.0 equiv.) and benzhydroxamic acid (16.5 mg, 0.12 mmol, 1.2 equiv.). Cs_2CO_3 (39.1 mg, 1.2 mmol, 1.2 equiv.) was added, followed by THF (1 mL) and the mixture was stirred for 16 h at desired temperature. The crude material was passed through a pad of Celite using EtOAc as eluent. The yield was determined by ^1H NMR analysis using dibromomethane (7.0 µL, 17.4 mg, 0.1 mmol) as internal standard.

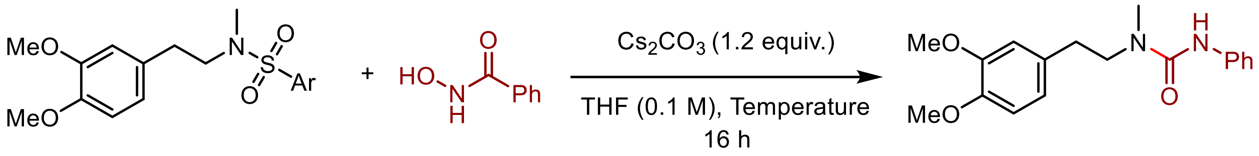

1a, **4** or **5** **2a** **3a**

Table 3. Reactivity of Ns-, ^f^Xs- and Nms-amides in deprotection-Lossen rearrangement

Temperature: 23 °C			
**Ar**	**4-NO$_2$ (4)**	**2,6-CF$_3$ (5)**	**2,4,6-CF$_3$ (1)**
**NMR Yield (%)**	0	0	98

Table 4. Reactivity of Ns- and ^f^Xs-amides in deprotection-Lossen rearrangement at 80 °C

Temperature: 80 °C		
**Ar**	**4-NO$_2$ (5)**	**2,6-CF$_3$ (6)**
**NMR Yield (%)**	0	33

5. Experimental

5.1. General Procedures

General procedure (GP1) for synthesis of hydroxamic acids starting from carboxylic acids:

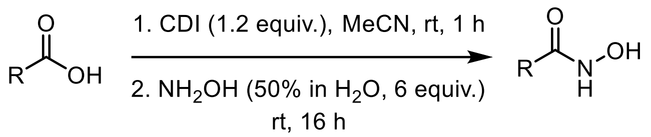

Carboxylic acid (1.0 equiv.) and CDI (1.2 equiv.) were dissolved in MeCN (0.25 M) and the resulting solution was stirred at 23 °C for 1 h. Then, a solution of NH_2OH (50% in water, 6 equiv.) was added to the flask and the reaction mixture was continued to stir at room temperature. After 16 h, the crude reaction mixture was diluted with water (20 mL) and EtOAc (20 mL) and the layers separated. The aqueous phase was extracted with EtOAc (2 x 20 mL). The combined organic phases were dried over anhydrous Na_2SO_4 and concentrated *in vacuo*. The crude material was purified by column chromatography (silica gel, heptane/EtOAc).

General procedure (GP2) for synthesis of hydroxamic acids starting from acyl chlorides:

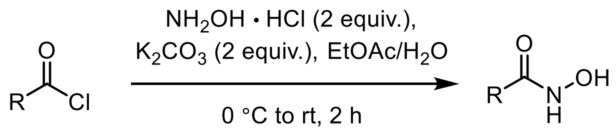

Hydroxylamine hydrochloride (2.0 equiv.), K_2CO_3 (2.0 equiv.), EtOAc and water were added sequentially to the flask. After cooling to 0 °C, acyl chloride (1.0 equiv.) was added dropwise. The reaction mixture was stirred at room temperature for 2 h and then diluted with water (20 mL) and EtOAc (20 ml) and separated. The aqueous phase was extracted additional times with EtOAc (2 x 20 mL). The combined organic phases were dried over anhydrous Na_2SO_4 and concentrated *in vacuo*. The crude material was purified by recrystallization from (heptane/EtOAc).

General procedure (GP3) for deprotective Lossen:

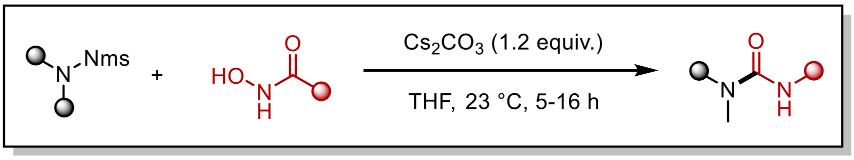

A vial was loaded with sulfonamide (1.0 equiv.) and hydroxamic acid (1.2 equiv.). Cs_2CO_3 (1.2 equiv.) was added, followed by THF (0.1 M). The mixture was stirred for 16 h at room temperature. The crude material was passed through a pad of Celite using EtOAc as eluent. After evaporation of the solvent, the crude material was subjected to column chromatography (silica gel, heptane/EtOAc) to obtain the desired urea products.

Note: The reaction was performed open to air.

5.2. Characterization of Hydroxamic Acids

***N*-Hydroxy-3-phenylpropanamide (2b)**

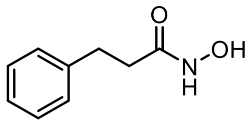

Following the GP2 (5 mmol scale), the titled compound was obtained as a white solid (570 mg, 3.45 mmol, 69%).

^{1}H NMR (400 MHz, DMSO-*d*$_6$): δ 10.37 (br, 1H), 8.70 (br, 1H), 7.30 – 7.16 (m, 5H), 2.81(t, *J* = 7.7 Hz, 2H), 2.26 (t, *J* = 7.7 Hz, 2H) ppm.

^{13}C NMR (101 MHz, DMSO-*d*$_6$): δ 168.2, 141.0, 128.3, 128.2, 125.9, 33.8, 30.8 ppm.

All NMR data were in accordance with the literature.[4]

***N*-Hydroxycyclohexanecarboxamide (2c)**

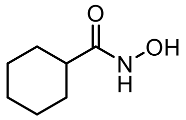

Following the GP1 (5 mmol scale), the titled compound was obtained as a white solid (366 mg, 2.56 mmol, 51%).

^{1}H NMR (400 MHz, DMSO-*d*$_6$): δ 10.32 (br, 1H), 8.61 (br, 1H), 1.96 (tt, J = 11.6, 3.3 Hz, 1H), 1.71 – 1.59 (m, 5H), 1.37 – 1.15 (m, 5H) ppm.

^{13}C NMR (101 MHz, DMSO-*d*$_6$): δ 172.3, 41.2, 29.1, 25.4, 25.3 ppm.

All NMR data were in accordance with the literature.[4]

***N*-Hydroxypivalamide (2d)**

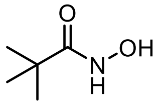

Following the GP2 (5 mmol scale), the titled compound was obtained as a white solid (177 mg, 1.51 mmol, 30%).

^{1}H NMR (400 MHz, DMSO-*d*$_6$): δ 10.29 (br, 1H), 8.52 (br, 1H), 1.08 (s, 9H) ppm.

^{13}C NMR (101 MHz, DMSO-*d*$_6$): δ 174.4, 36.9, 27.2 ppm.

All NMR data were in accordance with the literature.[4]

***N*-Hydroxy-2-phenylacetamide (2e)**

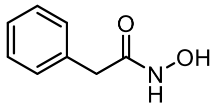

Following the GP2 (5 mmol scale), the titled compound was obtained as a white solid (383 mg, 2.53 mmol, 51%).

^{1}H NMR (400 MHz, DMSO-*d*$_6$): δ 11.01 (br, 1H), 8.88 (br, 1H), 7.20–7.30 (m, 5H), 3.27 (s, 2H) ppm.

^{13}C NMR (101 MHz, DMSO-*d*$_6$): δ 167.2, 136.3, 130.0, 128.2, 126.5, 39.1 ppm.

All NMR data were in accordance with the literature.[4]

***N*-Hydroxycinnamamide (2f)**

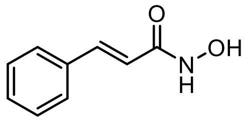

Following the GP1 (5 mmol scale), the titled compound was obtained as a white solid (292 mg, 1.79 mmol, 36%).

^{1}H NMR (400 MHz, DMSO-*d*$_6$): δ 10.76 (br, 1H), 9.05 (br, 1H), 7.57– 7.36 (m, 6H), 6.47 (d, J = 15.8 Hz, 1H) ppm.

^{13}C NMR (101 MHz, DMSO-*d*$_6$): δ 162.7, 138.3, 134.8, 129.5, 128.9, 127.5, 119.1 ppm.

All NMR data were in accordance with the literature.[4]

***N*-Hydroxyquinoline-2-carboxamide (2g)**

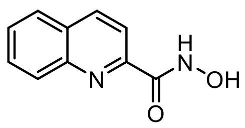

Following the GP1 (5 mmol scale), the titled compound was obtained as a white solid (755 mg, 4.01 mmol, 80%).

^{1}H NMR (400 MHz, DMSO-*d*$_6$): δ 11.51 (br, 1 H), 9.18 (br, 1 H), 8.53 (d, J = 8.5 Hz, 1 H), 8.09−8.04 (m, 3H), 7.84 (m, 1 H), 7.69 (m, 1H) ppm.

^{13}C NMR (101 MHz, DMSO-*d*$_6$): δ 161.7, 150.3, 146.0, 137.6, 130.4, 129.2, 128.6, 128.0, 127.9, 118.7 ppm.

All NMR data were in accordance with the literature.[5]

2-(3-Cyano-4-isobutoxyphenyl)-*N*-hydroxy-4-methylthiazole-5-carboxamide (2h)

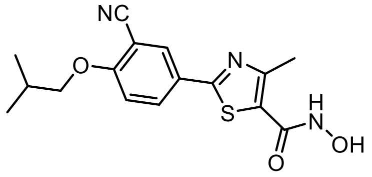

Following the GP1 (5 mmol scale), the titled compound was obtained as a white solid (595 mg, 1.80 mmol, 36%).

^{1}H NMR (400 MHz, CD_3OD): δ 8.17 – 8.07 (m, 2H), 7.21 (d, *J* = 8.9 Hz, 1H), 3.96 (d, *J* = 6.4 Hz, 2H), 2.69 (s, 3H), 2.16 (hept, *J* = 6.6 Hz, 1H), 1.09 (d, *J* = 6.7 Hz, 6H) ppm.

^{13}C NMR (101 MHz, CD_3OD): δ 168.7, 164.7, 164.0, 161.4, 134.2, 132.8, 127.1, 124.2, 116.4, 114.4, 103.5, 76.8, 29.4, 19.3, 17.3 ppm.

IR (neat): ν_{max} 1686, 1605, 1510, 1427, 1370, 1280, 1114, 1009, 765 cm^{-1}.

HRMS (ESI$^+$): exact mass calculated for $[M+H]^+$ $(C_{16}H_{18}N_3O_3S)^+$ requires *m/z* 332.1063, found *m/z* 332.1063.

(*E*)-*N*-Hydroxy-6-(4-hydroxy-6-methoxy-7-methyl-3-oxo-1,3-dihydroisobenzofuran-5-yl)-4-methylhex-4-enamide (2i)

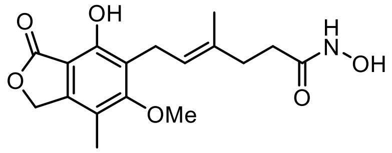

Following the GP1 (1 mmol scale), the titled compound was obtained as a white solid (232 mg, 0.69 mmol, 69%).

^{1}H NMR (400 MHz, $CDCl_3$): δ 8.41 (br, 1H), 7.84 (br, 2H), 5.26 (t, *J* = 6.6 Hz, 1H), 5.20 (s, 2H), 3.77 (s, 3H), 3.39 (d, *J* = 6.8 Hz, 2H), 2.29 (app s, 4H), 2.15 (s, 3H), 1.79 (s, 3H) ppm.

^{13}C NMR (101 MHz, $CDCl_3$): δ 173.0, 171.0, 163.7, 153.7, 144.4, 134.2, 124.1, 122.0, 117.1, 106.7, 70.2, 61.2, 34.8, 31.7, 22.8, 16.1, 11.7 ppm.

IR (neat): ν_{max} 1727, 1673, 1633, 1290, 1141, 1080, 1029, 962, 574 cm^{-1}.

HRMS (ESI$^+$): exact mass calculated for $[M+Na]^+$ $(C_{17}H_{21}NO_6Na)^+$ requires *m/z* 358.1261, found *m/z* 358.1262.

5.3. Characterization of Ureas

1-(3,4-Dimethoxyphenethyl)-1-methyl-3-phenylurea (3a)

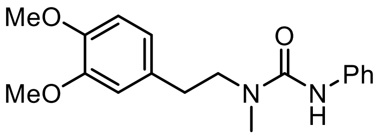

Following the GP3 (0.1 mmol scale), the titled compound was obtained as a colourless oil (30.2 mg, 96 µmol, 96%).

^{1}H NMR (400 MHz, CDCl$_3$): δ 7.25 – 7.16 (m, 4H), 6.99 (t, *J* = 7.1 Hz, 1H), 6.85 – 6.76 (m, 3H), 5.98 (s, 1H), 3.86 (s, 3H), 3.85 (s, 3H), 3.57 (t, *J* = 7.0 Hz, 2H), 2.96 (s, 3H), 2.85 (t, *J* = 7.0 Hz, 2H) ppm.

^{13}C NMR (101 MHz, CDCl$_3$): δ 155.0, 148.7, 147.3, 138.5, 131.1, 128.2, 122.2, 120.3, 119.1, 111.5, 111.1, 55.4 (2C), 51.2, 34.4, 33.6 ppm.

IR (neat): ν_{max} 3323, 2932, 1642, 1513, 1438, 1233, 1140, 1025, 751 cm^{-1}.

HRMS (ESI$^+$): exact mass calculated for $[M+Na]^+$ ($C_{18}H_{22}N_2O_3Na$)$^+$ requires *m/z* 337.1523, found *m/z* 337.1521.

1-(3,4-Dimethoxyphenethyl)-1-methyl-3-phenethylurea (3b)

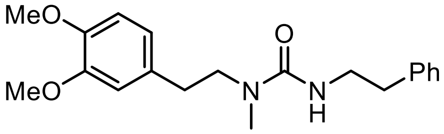

Following the GP3 (0.1 mmol scale), the titled compound was obtained as a colourless oil (31.6 mg, 92 µmol, 92%).

^{1}H NMR (400 MHz, CDCl$_3$): δ 7.33 – 7.27 (m, 2H), 7.24 – 7.15 (m, 3H), 6.78 (d, *J* = 8.6 Hz, 1H), 6.73 – 6.67 (m, 2H), 4.21 (t, *J* = 5.5 Hz, 1H), 3.86 (s, 3H), 3.85 (s, 3H), 3.50 – 3.39 (m, 4H), 2.81 – 2.70 (m, 7H) ppm.

^{13}C NMR (101 MHz, CDCl$_3$): δ 158.0, 149.1, 147.7, 139.6, 131.9, 129.0, 128.7, 126.5, 120.8, 112.2, 111.5, 56.0 (2C), 51.2, 42.1, 36.5, 34.8, 34.3 ppm.

IR (neat): ν_{max} 3347, 2932, 1627, 1512, 1453, 1260, 1232, 1138, 700 cm^{-1}.

HRMS (ESI$^+$): exact mass calculated for $[M+Na]^+$ ($C_{20}H_{26}N_2O_3Na$)$^+$ requires *m/z* 365.1836, found *m/z* 365.1834.

3-Cyclohexyl-1-(3,4-dimethoxyphenethyl)-1-methylurea (3c)

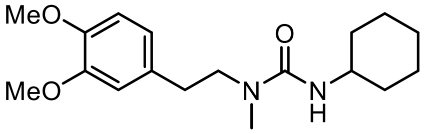

Following the GP3 (0.1 mmol scale), the titled compound was obtained as a white solid (30.9 mg, 96 µmol, 96%).

^{1}H NMR (400 MHz, $CDCl_3$): δ 6.79 (d, *J* = 8.0 Hz, 1H), 6.75 – 6.69 (m, 2H), 3.94 (d, *J* = 7.5 Hz, 1H), 3.86 (s, 3H), 3.85 (s, 3H), 3.62 – 3.50 (m, 1H), 3.44 (t, *J* = 7.2 Hz, 2H), 2.80 (s, 3H), 2.76 (t, *J* = 7.2 Hz, 2H), 1.90 – 1.81 (m, 2H), 1.69 – 1.54 (m, 3H), 1.39 – 1.26 (m, 2H), 1.17 – 1.05 (m, 1H), 1.03 – 0.90 (m, 2H) ppm.

^{13}C NMR (101 MHz, $CDCl_3$): δ 157.4, 149.0, 147.6, 132.0, 120.7, 112.1, 111.4, 55.9, 55.9, 51.3, 49.2, 34.6, 34.2, 34.0, 25.7, 25.0 ppm.

IR (neat): ν_{max} 2935, 2851, 1624, 1512, 1264, 1154, 1132, 1026 cm^{-1}.

HRMS (ESI^{+}): exact mass calculated for $[M+Na]^{+}$ $(C_{18}H_{28}N_2O_3Na)^{+}$ requires *m/z* 343.1992, found *m/z* 343.1990.

3-(*Tert*-butyl)-1-(3,4-dimethoxyphenethyl)-1-methylurea (3d)

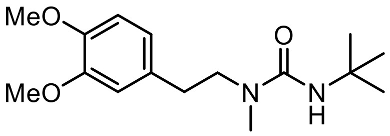

Following the GP3 (0.1 mmol scale), the titled compound was obtained as a colourless oil (25.2 mg, 86 µmol, 86%).

^{1}H NMR (400 MHz, $CDCl_3$): δ 6.80 (d, *J* = 7.8 Hz, 1H), 6.75 – 6.69 (m, 2H), 3.85 (s, 4H), 3.85 (s, 3H), 3.41 (t, *J* = 7.0 Hz, 2H), 2.80 (s, 3H), 2.75 (t, *J* = 7.0 Hz, 2H) , 1.22 (s, 9H) ppm.

^{13}C NMR (101 MHz, $CDCl_3$): δ 157.6, 149.2, 147.8, 132.2, 120.9, 112.1, 111.6, 56.1, 56.0, 51.4, 50.5, 34.7, 34.3, 29.5 ppm.

IR (neat): ν_{max} 2960, 1632, 1513, 1452, 1261, 1235, 1141, 1027, 763 cm^{-1}.

HRMS (ESI^{+}): exact mass calculated for $[M+Na]^{+}$ $(C_{16}H_{26}N_2O_3Na)^{+}$ requires *m/z* 317.1836, found *m/z* 317.1834.

3-Benzyl-1-(3,4-dimethoxyphenethyl)-1-methylurea (3e)

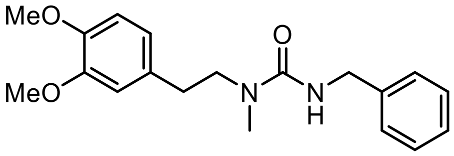

Following the GP3 (0.1 mmol scale), the titled compound was obtained as a colourless oil (32.0 mg, 97 µmol, 97%).

^{1}H NMR (400 MHz, $CDCl_3$): δ 7.35 – 7.28 (m, 2H), 7.28 – 7.21 (m, 3H), 6.77 (d, *J* = 7.9 Hz, 1H), 6.75 – 6.67 (m, 2H), 4.46 (t, *J* = 5.0 Hz, 1H), 4.35 (d, *J* = 5.5 Hz, 2H), 3.84 (s, 3H), 3.82 (s, 3H), 3.50 (t, *J* = 7.2 Hz, 2H), 2.84 (s, 3H), 2.78 (t, *J* = 7.2 Hz, 2H) ppm.

^{13}C NMR (101 MHz, $CDCl_3$): δ 158.1, 149.1, 147.7, 139.7, 131.9, 128.7, 127.8, 127.3, 120.8, 112.1, 111.4, 56.0 (2C), 51.3, 45.1, 34.8, 34.3 ppm.

IR (neat): ν_{max} 3345, 2931, 1628, 1512, 1453, 1260, 1232, 1138, 698 cm^{-1}.

HRMS (ESI$^+$): exact mass calculated for $[M+Na]^+$ $(C_{19}H_{24}N_2O_3Na)^+$ requires *m/z* 351.1679, found *m/z* 351.1678.

(*E*)-1-(3,4-Dimethoxyphenethyl)-1-methyl-3-styrylurea (3f)

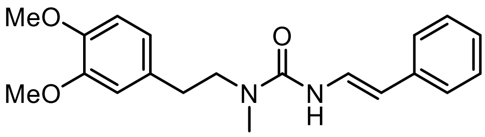

Following the GP3 (0.1 mmol scale), the titled compound was obtained as a colourless oil (25.6 mg, 75 µmol, 75%).

^{1}H NMR (400 MHz, $CDCl_3$): δ 7.44 (dd, *J* = 14.6, 10.6 Hz, 1H), 7.25 (s, 4H), 7.15 – 7.09 (m, 1H), 6.83 (d, *J* = 7.9 Hz, 1H), 6.80 – 6.74 (m, 2H), 6.14 (d, *J* = 10.5 Hz, 1H), 5.75 (d, *J* = 14.6 Hz, 1H), 3.89 (s, 3H), 3.85 (s, 3H), 3.54 (t, *J* = 7.0 Hz, 2H), 2.91 (s, 3H), 2.83 (t, *J* = 7.0 Hz, 3H) ppm.

^{13}C NMR (101 MHz, $CDCl_3$): δ 154.6, 149.4, 148.1, 137.0, 131.7, 128.7, 125.9, 125.6, 125.2, 121.0, 112.2 111.7, 109.0, 56.1 (2C), 51.8, 35.1, 34.4 ppm.

IR (neat): ν_{max} 1710, 1631, 1513, 1453, 1263, 1137, 1026, 760, 530 cm^{-1}.

HRMS (ESI$^+$): exact mass calculated for $[M+Na]^+$ $(C_{20}H_{24}N_2O_3Na)^+$ requires *m/z* 363.1679, found *m/z* 363.1677.

1-(3,4-Dimethoxyphenethyl)-1-methyl-3-(quinolin-2-yl)urea (3g)

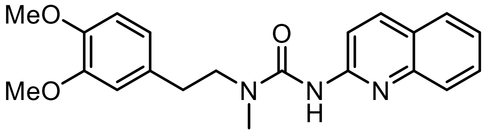

Following the GP3 (0.1 mmol scale), the titled compound was obtained as a colourless oil (35.9 mg, 98 µmol, 98%).

^{1}H NMR (400 MHz, CD_3OD): δ 8.15 (d, *J* = 9.0 Hz, 1H), 7.87 (d, *J* = 9.0 Hz, 1H), 7.78 (d, *J* = 8.0 Hz, 1H), 7.73 (d, *J* = 8.4 Hz, 1H), 7.68 – 7.61 (m, 1H), 7.45 – 7.38 (m, 1H), 6.88 (s, 1H), 6.80 (s, 2H), 3.79 (s, 3H), 3.70 – 3.61 (m, 5H), 3.02 (s, 3H), 2.86 (t, *J* = 7.1 Hz, 2H) ppm.

^{13}C NMR (101 MHz, CD_3OD): 157.4, 154.0, 150.5, 149.2, 147.0, 139.4, 133.1, 131.2, 128.8, 127.1, 126.8, 125.9, 122.4, 115.8, 113.9, 113.2, 56.4 (2C), 52.2, 35.4, 34.7 ppm.

IR (neat): ν_{max} 2995, 2834, 1664, 1600, 1495, 1421, 1261, 1112, 753 cm^{-1}.

HRMS (ESI$^+$): exact mass calculated for $[M+H]^+$ $(C_{21}H_{24}N_3O_3)^+$ requires *m/z* 366.1812, found *m/z* 366.1809.

3-(2-(3-Cyano-4-isobutoxyphenyl)-4-methylthiazol-5-yl)-1-(3,4-dimethoxyphenethyl)-1-methylurea (3h)

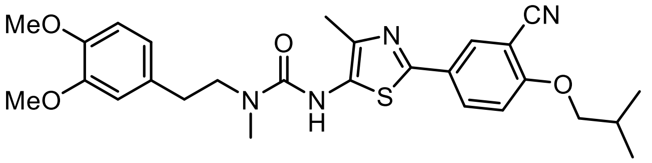

Following the GP3 (0.1 mmol scale), the titled compound was obtained as a pale-yellow oil (30.1 mg, 59 µmol, 59%).

^{1}H NMR (400 MHz, $CDCl_3$): δ 8.06 (d, *J* = 2.2 Hz, 1H), 7.92 (dd, *J* = 8.8, 2.3 Hz, 1H), 6.94 (d, *J* = 8.9 Hz, 1H), 6.84 – 6.73 (m, 3H), 6.13 (s, 1H), 3.88 – 3.81 (m, 8H), 3.59 (t, *J* = 7.0 Hz, 2H), 3.01 (s, 3H), 2.86 (t, *J* = 7.0 Hz, 2H), 2.24 (s, 3H), 2.17 (hept, *J* = 6.7 Hz, 1H), 1.07 (d, *J* = 6.7 Hz, 6H) ppm.

^{13}C NMR (101 MHz, $CDCl_3$): δ 161.3, 156.9, 154.4, 149.4, 148.1, 137.7, 131.5, 131.3, 130.9, 130.2, 127.5, 120.8, 116.0, 112.6, 112.1, 111.8, 102.7, 75.6, 56.1 (2C), 52.0, 35.1, 34.1, 28.3, 19.2, 14.5 ppm.

IR (neat): ν_{max} 2958, 2227, 1645, 1504, 1260, 1234, 1126, 1011, 730 cm^{-1}.

HRMS (ESI$^+$): exact mass calculated for $[M+Na]^+$ $(C_{27}H_{32}N_4O_4SNa)^+$ requires *m/z* 531.2036, found *m/z* 531.2046.

(*E*)-1-(3,4-dimethoxyphenethyl)-3-(5-(4-hydroxy-6-methoxy-7-methyl-3-oxo-1,3-dihydroisobenzofuran-5-yl)-3-methylpent-3-en-1-yl)-1-methylurea (3i)

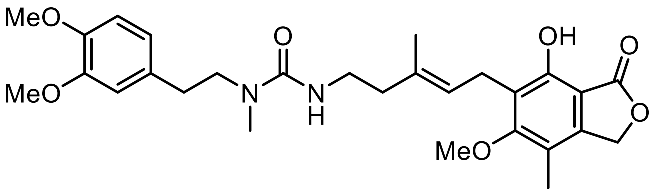

Following the GP3 (0.1 mmol scale), the titled compound was obtained as a colourless oil (21.6 mg, 42 µmol, 42%). In addition, unreacted starting material was recovered (20.6 mg, 38 µmol, 38%)

^{1}H NMR (400 MHz, $CDCl_3$): δ 7.66 (s, 1H), 6.76 (d, *J* = 8.7 Hz, 1H), 6.70 – 6.63 (m, 2H), 5.24 (t, *J* = 6.5 Hz, 1H), 5.15 (s, 2H), 4.19 (t, *J* = 5.0 Hz, 1H), 3.84 (s, 6H), 3.75 (s, 3H), 3.43 – 3.34 (m, 4H), 3.27 (q, *J* = 6.4 Hz, 2H), 2.72 – 2.63 (m, 5H), 2.18 – 2.09 (m, 5H), 1.79 (s, 3H) ppm.

^{13}C NMR (101 MHz, $CDCl_3$): δ 173.0, 163.7, 158.0, 153.7, 149.0, 147.6, 144.2, 133.6, 132.0, 124.5, 122.2, 120.8, 117.0, 112.1, 111.4, 106.5, 70.2, 61.1, 56.0 (2C), 51.2, 39.8, 38.4, 34.6, 34.3, 22.8, 15.9, 11.7 ppm.

IR (neat): ν_{max} 3421, 2934, 1730, 1624, 1513, 1452, 1261, 1134, 1025 cm^{-1}.

HRMS (ESI$^+$): exact mass calculated for $[M+Na]^+$ $(C_{28}H_{36}N_2O_7Na)^+$ requires *m/z* 535.2415, found *m/z* 535.2416.

1-Phenethyl-3-phenylurea (7a)

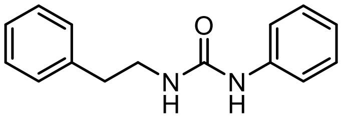

Following the GP3 (0.1 mmol scale), the titled compound was obtained as a white solid (20.4 mg, 85 µmol, 85%).

^{1}H NMR (400 MHz, CD_3OD): δ 7.35 – 7.17 (m, 9H), 6.96 (t, *J* = 7.3 Hz, 1H), 3.43 (t, *J* = 7.2 Hz, 2H), 2.82 (t, *J* = 7.2 Hz, 2H) ppm.

^{13}C NMR (101 MHz, CD_3OD): δ 158.3, 141.0, 140.7, 129.9, 129.8, 129.6, 127.4, 123.4, 120.1, 42.4, 37.4 ppm.

IR (neat): ν_{max} 2940, 1543, 1501, 1474, 1356, 749, 697 cm^{-1}.

HRMS (ESI$^+$): exact mass calculated for $[M+Na]^+$ $(C_{15}H_{16}N_2ONa)^+$ requires *m/z* 263.1155, found *m/z* 263.1154.

1-(2-(1*H*-Indol-3-yl)ethyl)-3-phenylurea (7b)

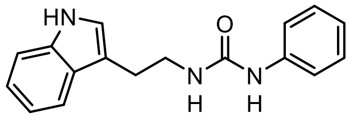

Following the GP3 (0.2 mmol scale), the titled compound was obtained as a white solid (47.8 mg, 171 µmol, 86%). In addition, unreacted starting material was recovered (11.0 mg, 22 µmol, 11%).

^{1}H NMR (600 MHz, Acetone-*d_6*): δ 10.03 (s, 1H), 7.95 (s, 1H), 7.62 (dd, *J* = 7.9, 0.7 Hz, 1H), 7.49 – 7.46 (m, 2H), 7.38 (dt, *J* = 8.1, 0.8 Hz, 1H), 7.22 – 7.17 (m, 3H), 7.09 (ddd, *J* = 8.1, 7.1, 1.1 Hz, 1H), 7.01 (ddd, *J* = 7.9, 7.0, 1.0 Hz, 1H), 6.92 – 6.87 (m, 1H), 5.84 (s, 1H), 3.54 (td, *J* = 7.1, 5.9 Hz, 2H), 2.99 – 2.94 (m, 2H) ppm.

^{13}C NMR (151 MHz, Acetone-*d_6*): δ 156.2, 141.8, 137.8, 129.4, 128.6, 123.4, 122.1 (2C), 119.4, 119.4, 118.9, 113.5, 112.1, 41.1, 26.9 ppm.

IR (neat) ν_{max}: 3381, 1641, 1594, 1566, 1499, 1442, 1310, 1241, 1217, 739 cm^{-1}.

HRMS (ESI$^+$): exact mass calculated for $[M+H]^+$ ($C_{17}H_{18}N_3O^+$) requires m/z 280.1444, found m/z 280.1443.

***Tert*-butyl (3-(3-phenylureido)propyl)carbamate (7c)**

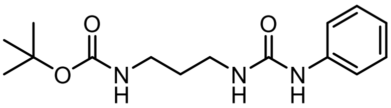

Following the GP3 (0.1 mmol scale), the titled compound was obtained as a white solid (24.2 mg, 83 µmol, 83%).

^{1}H NMR (400 MHz, Acetone-*d_6*): δ 8.04 (s, 1H), 7.51 – 7.43 (m, 2H), 7.25 – 7.09 (m, 2H), 6.93 – 6.85 (m, 1H), 6.09 (s, 1H), 5.90 (s, 1H), 3.24 (q, *J* = 6.2 Hz, 2H), 3.13 (q, *J* = 6.4 Hz, 2H), 1.66 – 1.58 (m, 2H), 1.40 (s, 9H) ppm.

^{13}C NMR (101 MHz, Acetone-*d_6*): δ 157.0, 156.5, 141.7, 129.4, 122.2, 119.0, 78.5, 38.0, 37.3, 31.7, 28.6 ppm.

IR (neat) ν_{max}: 3324, 1672, 1649, 1596, 1556, 1519, 1496, 1272, 1247, 1164, 753 cm^{-1}.

HRMS (ESI$^+$): exact mass calculated for $[M+Na]^+$ ($C_{15}H_{23}N_3NaO_3^+$) requires m/z 316.1632, found m/z 316.1636.

1-(Furan-2-ylmethyl)-3-phenylurea (7d)

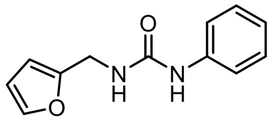

Following the GP3 (0.1 mmol scale), the titled compound was obtained as a white solid (19.6 mg, 91 µmol, 91%).

^{1}H NMR (400 MHz, Acetone-*d$_6$*): δ 7.98 (s, 1H), 7.51 – 7.43 (m, 3H), 7.25 – 7.16 (m, 2H), 6.92 (tt, *J* = 7.6, 1.1 Hz, 1H), 6.34 (dd, *J* = 3.2, 1.9 Hz, 1H), 6.25 (dd, *J* = 3.2, 0.8 Hz, 1H), 6.16 (s, 1H), 4.39 (d, *J* = 5.7 Hz, 2H) ppm.

^{13}C NMR (101 MHz, Acetone-*d$_6$*): δ 155.8, 154.3, 142.7, 141.5, 129.5, 122.4, 119.0, 111.2, 107.3, 37.4 ppm.

IR (neat) ν_{max}: 1633, 1593, 1568, 1442, 1421, 1002, 764, 723, 690 cm^{-1}.

HRMS (ESI$^+$): exact mass calculated for $[M+Na]^+$ ($C_{12}H_{12}N_2NaO_2^+$) requires m/z 239.0791, found m/z 239.0790.

1-Phenyl-3-(pyridin-2-ylmethyl)urea (7e)

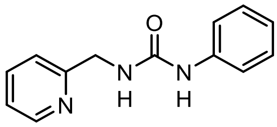

Following the GP3 (0.1 mmol scale), the titled compound was obtained as a white solid (22.3 mg, 98 µmol, 98%)

^{1}H NMR (400 MHz, Acetone-*d$_6$*): δ 8.54 – 8.45 (m, 1H), 8.34 (s, 1H), 7.74 (td, *J* = 7.7, 1.8 Hz, 1H), 7.54 – 7.49 (m, 2H), 7.39 (d, *J* = 7.9 Hz, 1H), 7.26 – 7.17 (m, 3H), 6.93 – 6.85 (m, 1H), 6.55 (s, 1H), 4.51 (d, *J* = 5.6 Hz, 2H) ppm.

^{13}C NMR (151 MHz, Acetone-*d$_6$*): δ 159.9, 156.3, 149.7, 141.7, 137.4, 129.4, 122.8, 122.3, 122.2, 119.0, 45.8 ppm.

IR (neat) ν_{max}: 3329, 3291, 1629, 1593, 1557, 753, 735, 692, 650 cm^{-1}.

HRMS (ESI$^+$): exact mass calculated for $[M+Na]^+$ ($C_{13}H_{13}N_3NaO^+$) requires m/z 250.0951, found m/z 250.0954.

(*R*)-1-(1-(Naphthalen-1-yl)ethyl)-3-phenylurea (7f)

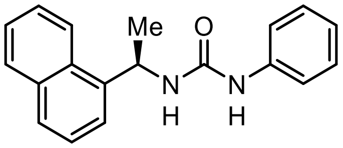

Following the GP3 (0.1 mmol scale), the titled compound was obtained as a white solid (28.0 mg, 96 µmol, 96%).

^{1}H NMR (700 MHz, Acetone-*d$_6$*): δ 8.25 (d, *J* = 8.5 Hz, 1H), 7.92 (d, *J* = 8.1 Hz, 1H), 7.86 – 7.78 (m, 2H), 7.63 (d, *J* = 7.1 Hz, 1H), 7.58 – 7.55 (m, 1H), 7.53 – 7.49 (m, 1H), 7.49 – 7.45 (m, 3H), 7.22 – 7.17 (m, 2H), 6.92 – 6.88 (m, 1H), 6.23 (d, *J* = 7.4 Hz, 1H), 5.82 (p, *J* = 7.0 Hz, 1H), 1.62 (d, *J* = 6.9 Hz, 3H) ppm.

^{13}C NMR (176 MHz, Acetone-*d$_6$*): δ 155.2, 141.6, 141.5, 135.0, 131.9, 129.6, 129.5, 128.4, 127.0, 126.5, 126.3, 124.3, 123.1, 122.2, 118.8, 45.9, 22.4 ppm.

IR (neat) ν_{max}: 3307, 1626, 1594, 1552, 1232, 773, 743 cm^{-1}.

HRMS (ESI$^+$): exact mass calculated for $[M+H]^+$ ($C_{19}H_{19}N_2O^+$) requires m/z 291.1492, found m/z 291.1485.

Benzyl (phenylcarbamoyl)-*L*-serinate (7g)

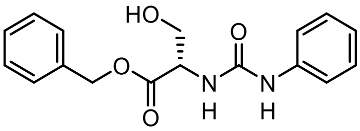

Following the GP3 (0.1 mmol scale), the titled compound was obtained as a colourless oil (21.7 mg, 69 µmol, 69%).

^{1}H NMR (600 MHz, Acetone-*d$_6$*): δ 8.27 (s, 1H), 7.51 – 7.47 (m, 2H), 7.44 – 7.39 (m, 2H), 7.37 – 7.29 (m, 3H), 7.25 – 7.21 (m, 2H), 6.93 (tt, *J* = 7.5, 1.1 Hz, 1H), 6.26 (d, *J* = 8.1 Hz, 1H), 5.20 (s, 2H), 4.56 (dt, *J* = 8.2, 3.6 Hz, 1H), 4.34 (s, 1H), 4.07 – 4.00 (m, 1H), 3.91 – 3.84 (m, 1H) ppm.

^{13}C NMR (151 MHz, Acetone-*d$_6$*): δ 172.1, 155.8, 141.4, 137.2, 129.5, 129.3, 128.8, 128.6, 122.5, 118.9, 67.1, 63.5, 56.2 ppm.

IR (neat) ν_{max}: 3509, 3319, 1719, 1641, 1062, 1051, 731 cm^{-1}.

HRMS (ESI$^+$): exact mass calculated for $[M+H]^+$ ($C_{17}H_{19}N_2O_4^+$) requires m/z 315.1339, found m/z 315.1337.

4-Bromo-*N*-phenylpiperidine-1-carboxamide (7h)

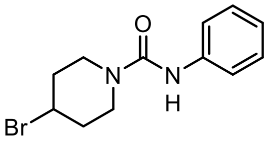

Following the GP3 (0.1 mmol scale), the titled compound was obtained as a white solid (19.5 mg, 69 µmol, 69%).

^{1}H NMR (400 MHz, Acetone-*d$_6$*): δ 7.98 (s, 1H), 7.55 – 7.43 (m, 2H), 7.26 – 7.13 (m, 2H), 7.03 – 6.87 (m, 1H), 4.59 – 4.42 (m, 1H), 3.90 – 3.79 (m, 2H), 3.43 – 3.29 (m, 2H), 2.24 – 2.14 (m, 2H), 1.98 – 1.88 (m, 2H) ppm.

^{13}C NMR (101 MHz, Acetone-*d$_6$*): δ 155.7, 141.6, 129.2, 122.7, 120.3, 51.0, 43.9, 36.9 ppm.

IR (neat) ν_{max}: 3299, 2915, 1632, 1622, 1518, 1446, 1242, 1230, 1011, 745, 691 cm^{-1}.

HRMS (ESI$^+$): exact mass calculated for [M+Na]$^+$ ($C_{12}H_{15}BrN_2NaO^+$) requires m/z 305.0260, found m/z 305.0262.

3-Oxo-*N*-phenyl-8-azabicyclo[3.2.1]octane-8-carboxamide (7i)

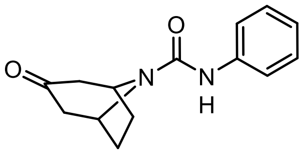

Following the GP3 (0.2 mmol scale), the titled compound was obtained as a white solid (46.0 mg, 188 µmol, 94%).

^{1}H NMR (400 MHz, Acetone-*d$_6$*): δ 8.18 (s, 1H), 7.61 – 7.54 (m, 2H), 7.28 – 7.21 (m, 2H), 7.00 – 6.93 (m, 1H), 4.76 – 4.59 (m, 2H), 2.83 – 2.77 (m, 2H), 2.29 – 2.23 (m, 2H), 2.14 – 2.07 (m, 2H), 1.72 – 1.66 (m, 2H) ppm.

^{13}C NMR (101 MHz, Acetone-*d$_6$*): δ 207.6, 154.4, 154.4, 141.3, 141.2, 129.3, 123.0, 122.9, 120.3, 120.2, 54.3, 49.0, 29.7 ppm.

IR (neat) ν_{max}: 3298, 1710, 1625, 1587, 1510, 1444, 1382, 1350, 996 cm^{-1}.

HRMS (ESI$^+$): exact mass calculated for [M+Na]$^+$ ($C_{14}H_{16}N_2NaO_2^+$) requires m/z 267.1104, found m/z 267.1102.

_N_-Phenyl-4-(4,4,5,5-tetramethyl-1,3,2-dioxaborolan-2-yl)piperidine-1-carboxamide (7j)

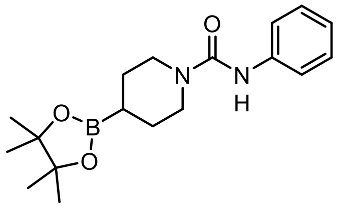

Following the GP3 (0.1 mmol scale), the titled compound was obtained as a white solid (24.1 mg, 73 µmol, 73%).

^{1}H NMR (400 MHz, Acetone-d_6): δ 7.82 (s, 1H), 7.52 (dd, *J* = 8.6, 1.0 Hz, 2H), 7.23 – 7.15 (m, 2H), 6.95 – 6.88 (m, 1H), 3.85 (dt, *J* = 13.0, 4.1 Hz, 2H), 3.09 (ddd, *J* = 13.1, 9.9, 3.1 Hz, 2H), 1.70 – 1.60 (m, 2H), 1.56 – 1.45 (m, 2H), 1.25 – 1.20 (m, 12H), 1.19 – 1.12 (m, 1H) ppm.

^{13}C NMR (101 MHz, Acetone-d_6): δ 155.8, 141.9, 129.1, 122.4 (2C), 120.2, 120.1 83.8, 46.0, 27.9, 25.1 ppm.

^{11}B NMR (193 MHz, Acetone-d_6): δ 33.7 ppm.

IR (neat) ν_{max}: 1638, 1537, 1500, 1444, 1244, 1139, 749 cm^{-1}.

HRMS (ESI$^+$): exact mass calculated for [M+H]$^+$ ($C_{18}H_{28}BN_2O_3^+$) requires m/z 331.2191, found m/z 331.2193.

4-((2*E*,4*E*)-5-(Benzo[d][1,3]dioxol-5-yl)penta-2,4-dienoyl)-N-phenylpiperazine-1-carboxamide (7k)

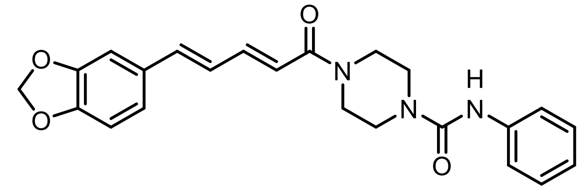

Following the GP3 (0.1 mmol scale), the titled compound was obtained as a pale yellow solid (39.0 mg, 96 µmol, 96%).

^{1}H NMR (700 MHz, CD_2Cl_2): δ 7.41 (ddd, *J* = 14.6, 7.3, 2.9 Hz, 1H), 7.37 (d, *J* = 7.7 Hz, 2H), 7.28 (t, *J* = 7.9 Hz, 2H), 7.04 (dd, *J* = 10.6, 4.1 Hz, 1H), 7.00 (d, *J* = 1.5 Hz, 1H), 6.90 (dd, *J* = 8.0, 1.5 Hz, 1H), 6.80 – 6.76 (m, 3H), 6.68 (s, 1H), 6.42 (d, *J* = 14.6 Hz, 1H), 5.98 (s, 2H), 3.77 – 3.44 (m, 8H) ppm.

^{13}C NMR (176 MHz, CD_2Cl_2): δ 166.2, 155.4, 149.0, 148.9, 144.0, 139.7, 131.3, 129.3, 125.5, 123.7, 123.3, 120.6, 119.5, 109.0, 106.1, 102.2, 45.9, 44.6, 44.1, 42.0 ppm.

IR (neat): ν_{max} 3361, 1629, 1534, 1501, 1443, 1250 cm^{-1}.

HRMS (ESI$^+$): exact mass calculated for [M+Na]$^+$ ($C_{23}H_{23}N_3O_4Na$)$^+$ requires *m/z* 428.1581, found *m/z* 428.1580.

(*S*)-1-Methyl-1-(3-(naphthalen-1-yloxy)-3-(thiophen-2-yl)propyl)-3-phenylurea (7l)

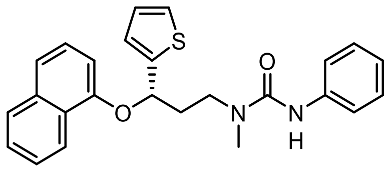

Following the GP3 (0.1 mmol scale), the titled compound was obtained as a white solid (41.5 mg, 99 μmol, 99%).

^{1}H NMR (600 MHz, $CDCl_3$): δ 8.40 – 8.33 (m, 1H), 7.81 – 7.73 (m, 1H), 7.53 – 7.45 (m, 2H), 7.40 (d, *J* = 8.2 Hz, 1H), 7.25 – 7.20 (m, 2H), 7.16 (t, *J* = 7.8 Hz, 2H), 7.10 – 7.07 (m, 3H), 6.97 – 6.92 (m, 2H), 6.86 (d, *J* = 7.7 Hz, 1H), 6.35 (s, 1H), 5.78 (dd, *J* = 8.4, 4.3 Hz, 1H), 3.73 – 3.57 (m, 2H), 3.00 (s, 3H), 2.57 – 2.39 (m, 2H) ppm.

^{13}C NMR (101 MHz, $CDCl_3$): δ 155.6, 152.7, 144.4, 139.0, 134.8, 128.8, 127.8, 126.8, 126.6, 126.2, 125.8, 125.6, 125.2, 125.1, 122.9, 121.9, 121.2, 119.8, 107.5, 73.7, 46.2, 37.4, 34.9 ppm.

IR (neat) ν_{max}: 1642, 1594, 1525, 1438, 1234, 1093, 691 cm^{-1}.

HRMS (ESI$^+$): exact mass calculated for $[M+H]^+$ ($C_{25}H_{25}N_2O_2S^+$) requires m/z 417.1631, found m/z 417.1625.

(3*S*,4*R*)-3-((Benzo[*d*][1,3]dioxol-5-yloxy)methyl)-4-(4-fluorophenyl)-*N*-phenylpiperidine-1-carboxamide (7m)

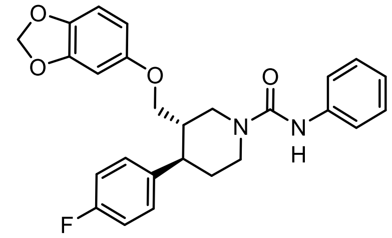

Following the GP3 (0.1 mmol scale), the titled compound was obtained as a white solid (44.4 mg, 99 μmol, 99%).

^{1}H NMR (400 MHz, Acetone-*d6*): δ 8.04 (s, 1H), 7.57 (dd, *J* = 8.6, 1.0 Hz, 2H), 7.35 – 7.29 (m, 2H), 7.27 – 7.20 (m, 2H), 7.09 – 7.02 (m, 2H), 6.98 – 6.91 (m, 1H), 6.66 (d, *J* = 8.5 Hz, 1H), 6.43 (d, *J* = 2.5 Hz, 1H), 6.22 (dd, *J* = 8.5, 2.5 Hz, 1H), 5.92 – 5.87 (m, 2H), 4.66 – 4.51 (m, 1H), 4.42 – 4.31 (m, 1H), 3.69 – 3.64 (m, 1H), 3.58 (dd, *J* = 9.6, 6.6 Hz, 1H), 3.01 – 2.81 (m, 3H), 2.21 – 2.12 (m, 1H), 1.84 – 1.77 (m, 2H) ppm.

^{13}C NMR (101 MHz, Acetone-*d6*): δ 162.44 (d, *J* = 242.7 Hz), 155.8, 155.7, 155.5, 149.2, 142.7, 141.8, 141.8, 140.9 (d, *J* = 2.5 Hz), 130.2 (d, *J* = 7.8 Hz), 129.2, 122.7, 122.7, 120.4, 120.3, 116.1 (d, *J* = 21.6 Hz), 108.7, 106.6, 102.1, 98.8, 70.0, 48.4, 45.6, 44.9, 42.8, 34.9 ppm.

^{19}F NMR (377 MHz, Acetone-d_6): δ -118.0 ppm.

IR (neat) ν_{max}: 1633, 1500, 1486, 1468, 1442, 1269, 1220, 1179, 1036, 830, 751, 693 cm^{-1}.

HRMS (ESI$^+$): exact mass calculated for $[M+H]^+$ ($C_{26}H_{26}FN_2O_4^+$) requires m/z 449.1871, found m/z 449.1873.

4-(8-Chloro-5,6-dihydro-11*H*-benzo[5,6]cyclohepta[1,2-*b*]pyridin-11-ylidene)-*N*-phenylpiperidine-1-carboxamide (7n)

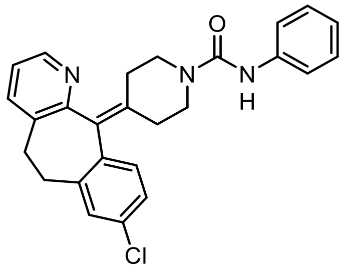

Following the GP3 (0.1 mmol scale), the titled compound was obtained as a white solid (42.0 mg, 98 µmol, 98%).

^{1}H NMR (400 MHz, CDCl$_3$): δ 8.40 (dd, *J* = 4.8, 1.6 Hz, 1H), 7.44 (dd, *J* = 7.7, 1.5 Hz, 1H), 7.34 – 7.31 (m, 2H), 7.27 – 7.25 (m, 2H), 7.18 – 7.08 (m, 4H), 7.04 – 6.97 (m, 1H), 6.48 (s, 1H), 3.82 – 3.69 (m, 2H), 3.44 – 3.30 (m, 2H), 3.29 – 3.18 (m, 2H), 2.89 – 2.74 (m, 2H), 2.67 – 2.55 (m, 1H), 2.51 – 2.33 (m, 3H) ppm.

^{13}C NMR (101 MHz, CDCl$_3$): δ 157.0, 155.0, 146.8, 139.7, 139.2, 137.7, 137.7, 137.0, 134.7, 133.5, 133.1, 130.6, 129.1, 128.9, 126.3, 123.2, 122.5, 120.1, 45.0, 44.9, 31.8, 31.6, 30.6, 30.4 ppm.

IR (neat) ν_{max}: 1710, 1639, 1595, 1532, 1499, 1478, 1440, 1227, 994, 752 cm^{-1}.

HRMS (ESI$^+$): exact mass calculated for $[M+H]^+$ ($C_{26}H_{25}ClN_3O^+$) requires m/z 430.1681, found m/z 430.1682.

4-(2-Chlorodibenzo[*b*,*f*][1,4]oxazepin-11-yl)-*N*-phenylpiperazine-1-carboxamide (7o)

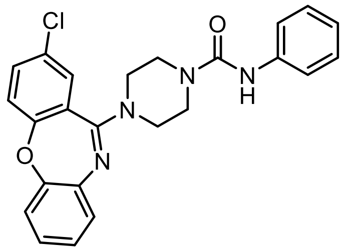

Following the GP3 (82 µmol scale), the titled compound was obtained as a white solid (34.6 mg, 80 µmol, 98%).

^{1}H NMR (400 MHz, Acetone-*d$_6$*): δ 8.04 (s, 1H), 7.59 – 7.50 (m, 4H), 7.38 (d, *J* = 8.6 Hz, 1H), 7.26 – 7.21 (m, 2H), 7.18 – 7.08 (m, 3H), 7.04 – 7.00 (m, 1H), 6.95 (t, *J* = 7.4 Hz, 1H), 3.78 – 3.55 (m, 8H) ppm.

^{13}C NMR (101 MHz, Acetone-*d$_6$*): δ 160.3, 159.6, 156.0, 152.7, 141.5, 141.2, 133.7, 130.9, 129.8, 129.2, 127.8, 126.6, 126.0, 125.4, 123.9, 122.8, 121.0, 120.3, 48.1, 44.6 ppm.

IR (neat) ν_{max}: 1624, 1587, 1526, 1467, 1450, 1412, 1252, 1235, 1211, 1086, 747 cm^{-1}.

HRMS (ESI$^+$): exact mass calculated for $[M+H]^+$ ($C_{24}H_{22}ClN_4O_2^+$) requires m/z 433.1426, found m/z 433.1428.

1-(2-(1*H*-Indol-3-yl)ethyl)-1-benzyl-3-phenylurea (9)

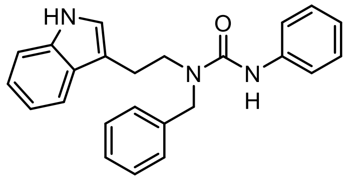

Following the GP3 (48 μmol scale), the titled compound was obtained as a white solid (14.2 mg, 38 μmol, 80%).

^{1}H NMR (400 MHz, Acetone-d_6): δ 10.08 (s, 1H), 7.64 (d, *J* = 7.9 Hz, 1H), 7.42 – 7.33 (m, 5H), 7.28 – 7.10 (m, 8H), 7.08 – 7.02 (m, 1H), 6.91 – 6.85 (m, 1H), 4.68 (s, 2H), 3.70 (t, *J* = 7.2 Hz, 2H), 3.08 (t, *J* = 7.2 Hz, 2H) ppm.

^{13}C NMR (101 MHz, Acetone-d_6): δ 156.3, 141.5, 140.0, 137.8, 129.3, 129.1, 128.5, 127.9, 124.0, 122.5, 122.4, 120.2, 119.8, 119.3, 112.4, 50.5, 48.4, 24.9 ppm.

IR (neat) ν_{max}: 3272, 1624, 1537, 1493, 1444, 1218, 1237, 1200, 749, 736 cm^{-1}.

HRMS (ESI$^+$): exact mass calculated for $[M+H]^+$ ($C_{24}H_{24}N_3O^+$) requires m/z 370.1914, found m/z 370.1916.

5.4. Unsuccessful Substrates

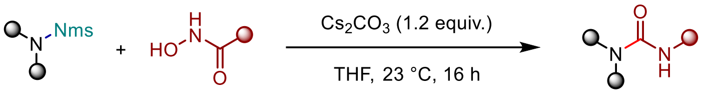

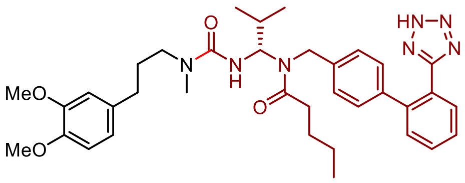

nd, decomposition

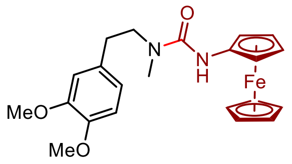

nd, only starting material left

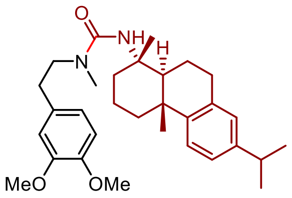

nd, only starting material left

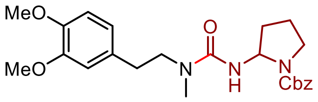

nd, decomposition

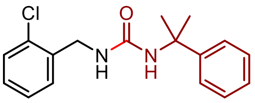

nd, decomposition

Reaction conditions: sulfonamide (0.1 mmol), hydroxamic acid (0.12 mmol), Cs_2CO_3 (0.12 mmol), THF (1 mL), 23 °C, 16 h, air atmosphere. nd = not determined.

5.5. Scale-up Procedure

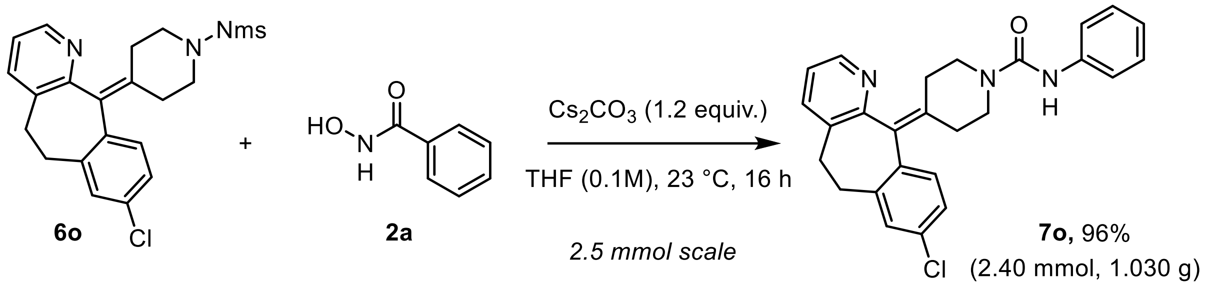

A 50 mL round-bottom flask was loaded with sulfonamide **1n** (1.636 g, 2.5 mmol, 1.0 equiv.), benzhydroxamic acid (411 mg, 3 mmol, 1.2 equiv.), Cs_2CO_3 (977 mg, 3 mmol, 1.2 equiv.) and THF (25 mL) was added. The flask was placed in a sand bath and the mixture was stirred for 16 h at 23 °C. The crude material was passed through a pad of Celite using EtOAc as eluent. After evaporation of the solvent, the crude material was subjected to column chromatography (silica gel, heptane/EtOAc) to afford the urea **8n** as a white solid (1.030 g, 2.40 mmol, 96%)

Note: Reaction was performed open to air.

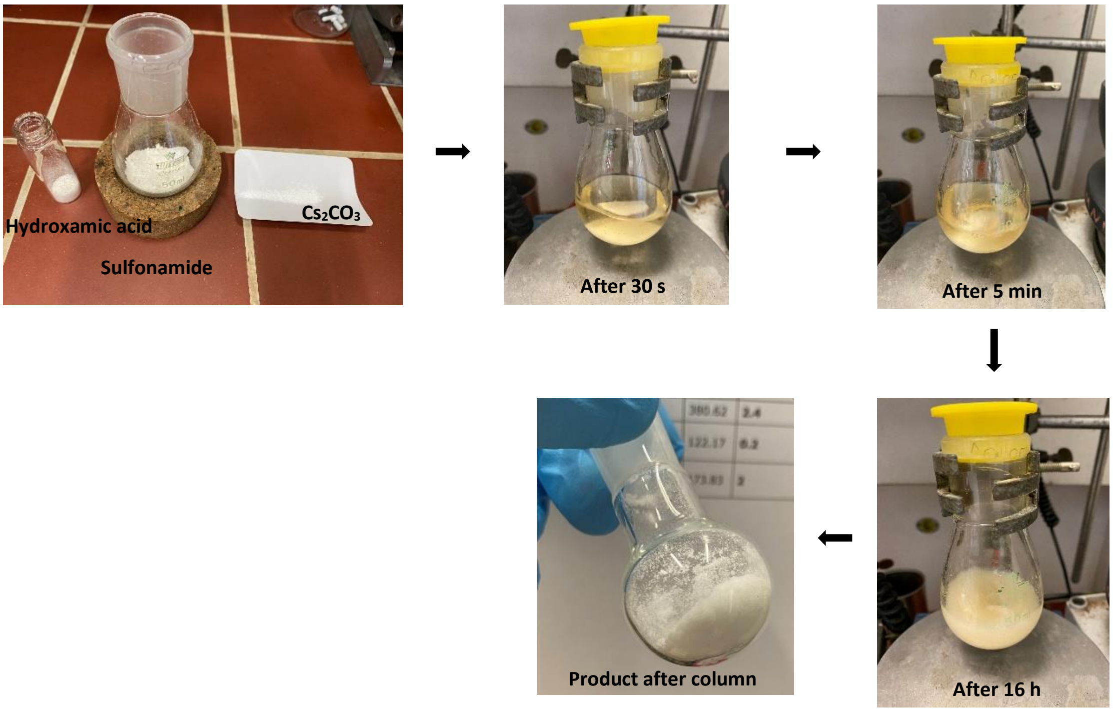

5.6. One-pot Urea Synthesis

3-Oxo-*N*-(quinolin-2-yl)-8-azabicyclo[3.2.1]octane-8-carboxamide (10)

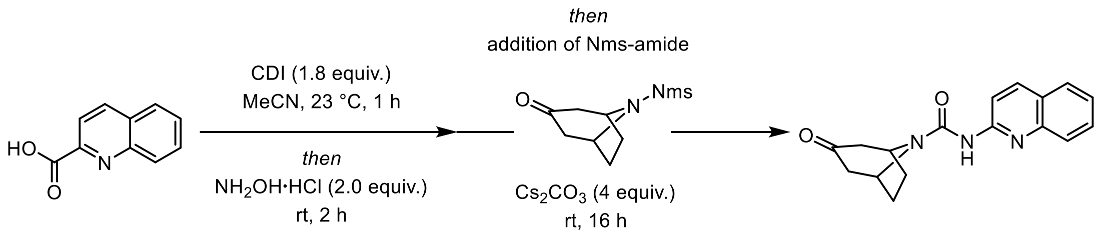

A vial (8 mL) was loaded with quinaldic acid (52.0 mg, 0.3 mmol, 1.5 equiv.) dissolved in MeCN (2 mL). Then, 1,1'-carbonyldiimidazole (CDI, 60.2 mg, 0.36 mmol, 1.8 equiv.) was added and the solution was stirred for 1 h. Hydroxylamine hydrochloride (27.8 mg, 0.4 mmol, 2.0 equiv.) was added and the resulting mixture was stirred for another 2 h. Finally, sulfonamide **6j** (93.9 mg, 0.2 mmol, 1.0 equiv.) and Cs_2CO_3 (261 mg, 0.8 mmol, 4.0 equiv.) were added and the solution was stirred for 16 h. The crude material was then passed through a Celite plug and, after solvent concentration, purified by column chromatography (heptane/EtOAc, 0-70%) to afford the titled compound as a colourless solid (46.7 mg, 167 µmol, 83%).

^{1}H NMR (600 MHz, $CDCl_3$): δ 8.44 – 7.31 (m, 7H), 4.70 (br, 2H), 2.81 (app dd, *J* = 16.1, 4.3 Hz, 2H), 2.42 (app d, *J* = 16.0 Hz, 2H), 2.19 – 2.12 (m, 2H), 1.76 (app d, *J* = 7.9 Hz, 2H) ppm.

^{13}C NMR (151 MHz, $CDCl_3$): δ 207.2, 152.1, 146.3, 138.9, 130.3, 127.8, 126.6, 124.9, 114.1, 53.7, 48.9, 29.2 ppm.

IR (neat) ν_{max}: 1660, 1491, 1421, 1351, 1316, 1267, 1241, 1200, 1186, 1104, 1002, 818, 747 cm^{-1}.

HRMS (ESI$^+$): exact mass calculated for $[M+H]^+$ ($C_{17}H_{18}N_3O_2^+$) requires m/z 296.1394, found m/z 296.1392.

5.7. Synthesis of ureido-based peptidomimetic

Methyl (((*S*)-1-((tert-butoxycarbonyl)amino)-2-phenylethyl)carbamoyl)-*L*-isoleucinate (12)

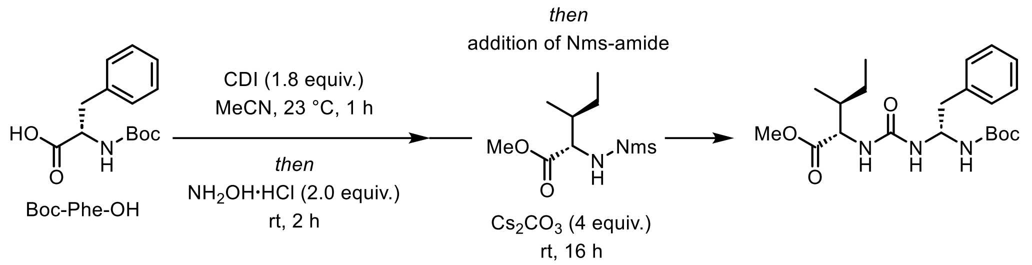

A vial (8 mL) was loaded with Boc-Phe-OH (59.7 mg, 0.225 mmol, 1.5 equiv.) dissolved in MeCN (1.5 mL). Then, 1,1'-carbonyldiimidazole (CDI, 45.1 mg, 0.27 mmol, 1.8 equiv.) was added and the solution was stirred for 1 h. Hydroxylamine hydrochloride (20.8 mg, 0.3 mmol, 2.0 equiv.) was added and the resulting mixture was stirred for another 2 h. Finally, sulfonamide **11** (73.4 mg, 0.15 mmol, 1.0 equiv.) and Cs_2CO_3 (195 mg, 0.6 mmol, 4.0 equiv.) were added and the solution was stirred for 16 h. The crude material was then passed through a Celite plug and, after solvent concentration, purified by column chromatography (heptane/EtOAc, 0-70%) to afford the titled compound as a colourless solid (60.9 mg, 91.3 µmol, 61%, >20:1 d.r.).

^{1}H NMR (400 MHz, $CDCl_3$): δ 7.32 – 7.16 (m, 5H), 5.97 (br s, 1H), 5.66 (br s, 1H), 5.34 (br s, 1H), 5.28 – 5.17 (m, 1H), 4.39 (dd, *J* = 8.1, 5.4 Hz, 1H), 3.70 (s, 3H), 3.15 – 3.00 (m, 2H), 1.90 – 1.77 (m, 1H), 1.44 – 1.38 (m, 1H), 1.37 (s, 9H), 1.23 – 1.12 (m, 1H), 0.88 (d, *J* = 7.1 Hz, 6H) ppm.

^{13}C NMR (101 MHz, $CDCl_3$): δ 173.5, 157.3, 155.8, 136.5, 129.5, 128.6, 127.0, 80.4, 60.3, 57.7, 52.0, 40.8, 37.9, 28.4, 25.2, 15.6, 11.7 ppm.

IR (neat) ν_{max}: 3341, 1692, 1557, 1518, 1248, 1171, 1011, 661 cm^{-1}.

HRMS (ESI$^+$): exact mass calculated for $[M+H]^+$ ($C_{21}H_{34}N_3O_5^+$) requires m/z 408.2493, found m/z 408.2487.

6. X-ray Crystallographic Data

X-ray of compound **7d**: **CCDC2269266**

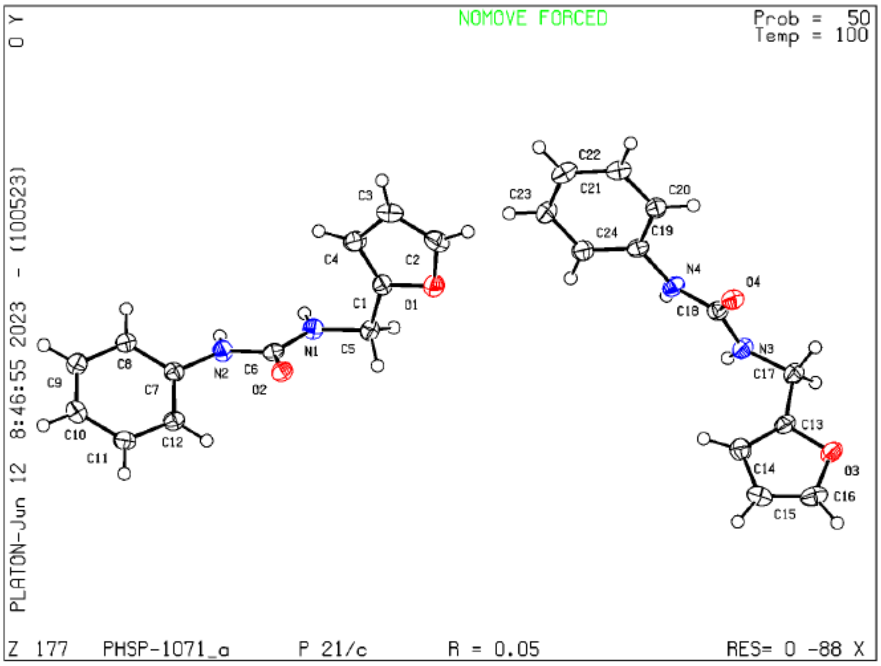

Bond precision: C-C = 0.0049 A Wavelength=1.54186

Cell:	a=21.3525(12)	b=8.6525(5)	c=11.7801(8)
	alpha=90	beta=105.852(5)	gamma=90
Temperature:	100 K		

	Calculated	Reported
Volume	2093.6(2)	2093.8(2)
Space group	P 21/c	P 21/c
Hall group	-P 2ybc	-P 2ybc
Moiety formula	C12 H12 N2 O2	?
Sum formula	C12 H12 N2 O2	C24 H24 N4 O4
Mr	216.24	432.47
Dx,g cm-3	1.372	1.372
Z	8	4
Mu (mm-1)	0.780	0.780
F000	912.0	912.0
F000’	914.85	
h,k,lmax	25,10,14	14,10,25
Nref	3870	3737
Tmin,Tmax	0.954,0.992	0.797,0.995
Tmin’	0.829	

Correction method= # Reported T Limits: Tmin=0.797 Tmax=0.995
AbsCorr = MULTI-SCAN

Data completeness= 0.966 Theta(max)= 68.588

R(reflections)= 0.0549(2313) wR2(reflections)= 0.1470(3737)

S = 1.039 Npar= 290

X-ray of compound **7j**: **CCDC2268806**

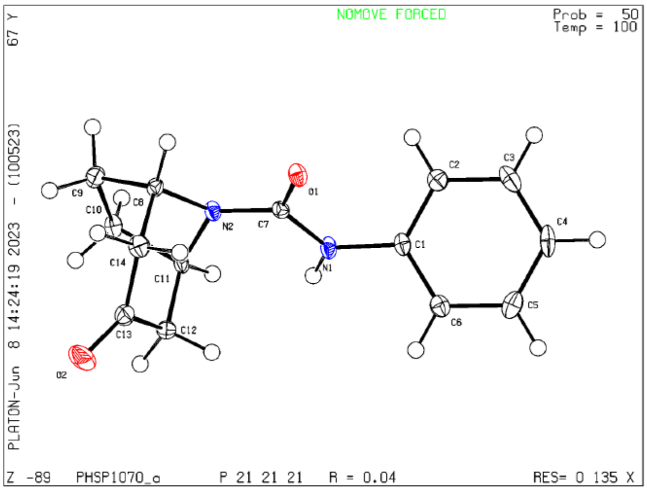

Bond precision: C-C = 0.0025 A Wavelength=0.71073

Cell:	a=9.8956(5)	b=10.1319(5)	c=11.9988(9)
	alpha=90	beta=90	gamma=90
Temperature:	100 K		

	Calculated	Reported
Volume	1203.02(12)	1203.01(12)
Space group	P 21 21 21	P 21 21 21
Hall group	P 2ac 2ab	P 2ac 2ab
Moiety formula	C14 H16 N2 O2	?
Sum formula	C14 H16 N2 O2	C14 H16 N2 O2
Mr	244.29	244.29
Dx,g cm-3	1.349	1.349
Z	4	4
Mu (mm-1)	0.091	0.091
F000	520.0	520.0
F000’	520.22	
h,k,lmax	14,14,17	14,14,17
Nref	3749[2141]	3565
Tmin,Tmax		0.990,0.997
Tmin’		

Correction method= # Reported T Limits: Tmin=0.990 Tmax=0.997
AbsCorr = SPHERE

Data completeness= 1.67/0.95 Theta(max)= 30.720

R(reflections)= 0.0378(2861) wR2(reflections)= 0.0825(3565)

S = 1.048 Npar= 163

7. NMR Spectra

2-(3-Cyano-4-isobutoxyphenyl)-*N*-hydroxy-4-methylthiazole-5-carboxamide (2i)

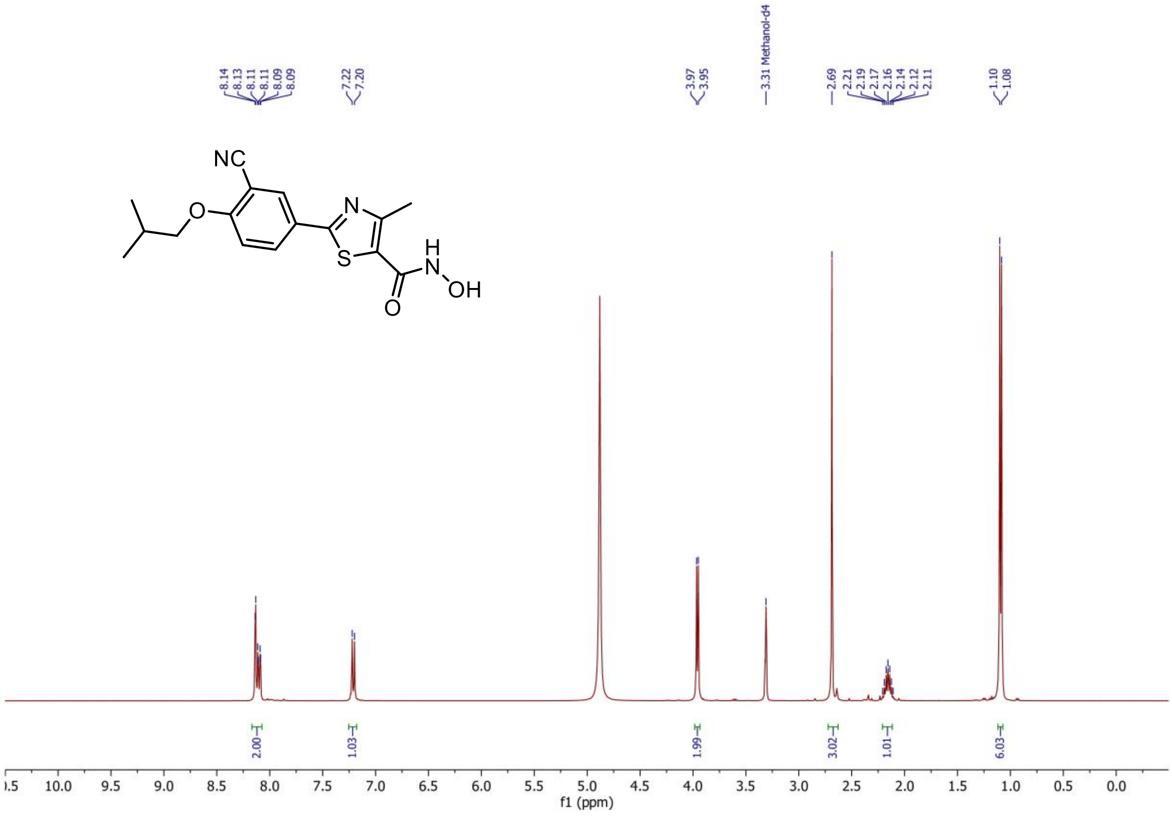

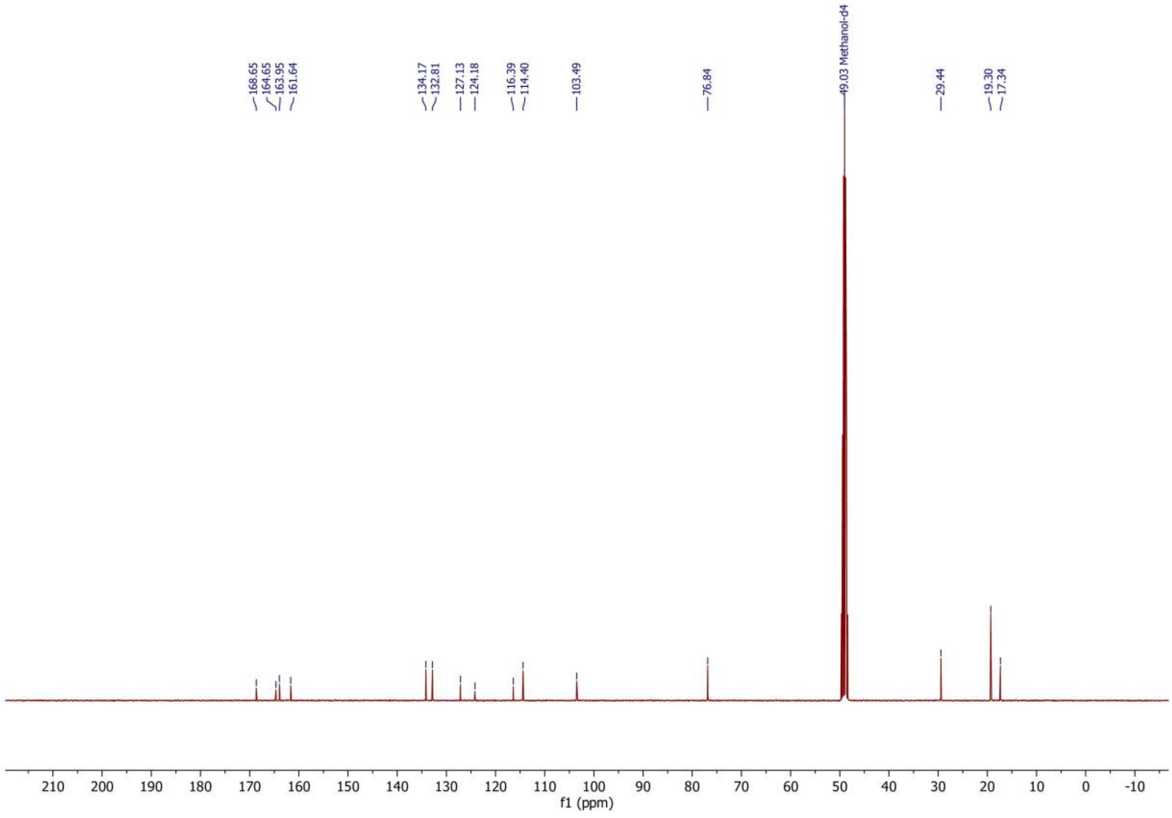

(*E*)-*N*-Hydroxy-6-(4-hydroxy-6-methoxy-7-methyl-3-oxo-1,3-dihydroisobenzofuran-5-yl)-4-methylhex-4-enamide (2j)

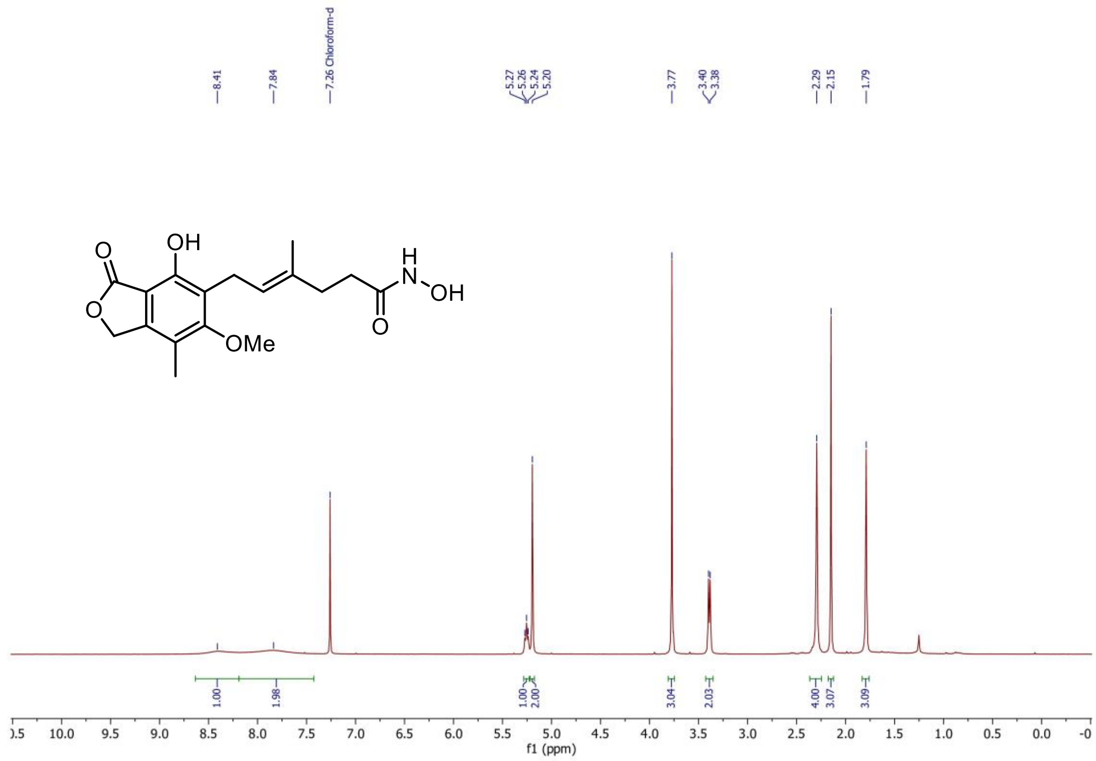

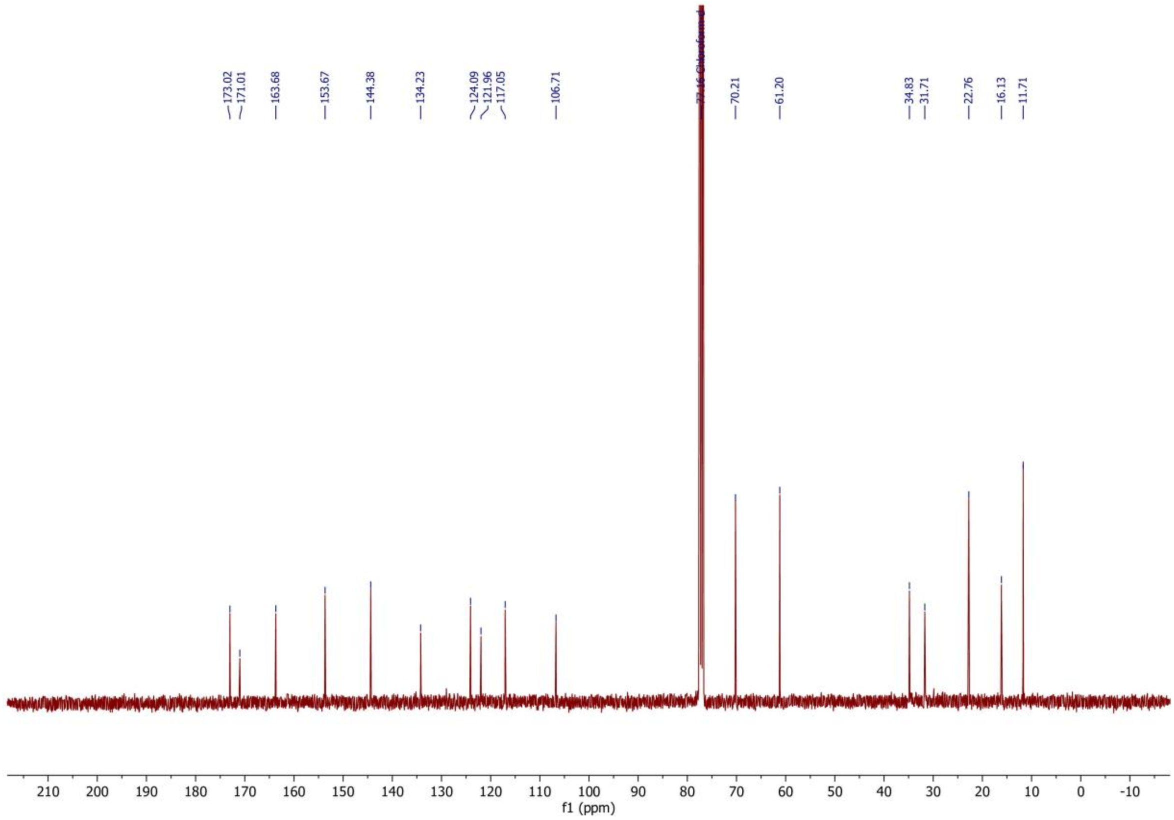

1-(3,4-Dimethoxyphenethyl)-1-methyl-3-phenylurea (3a)

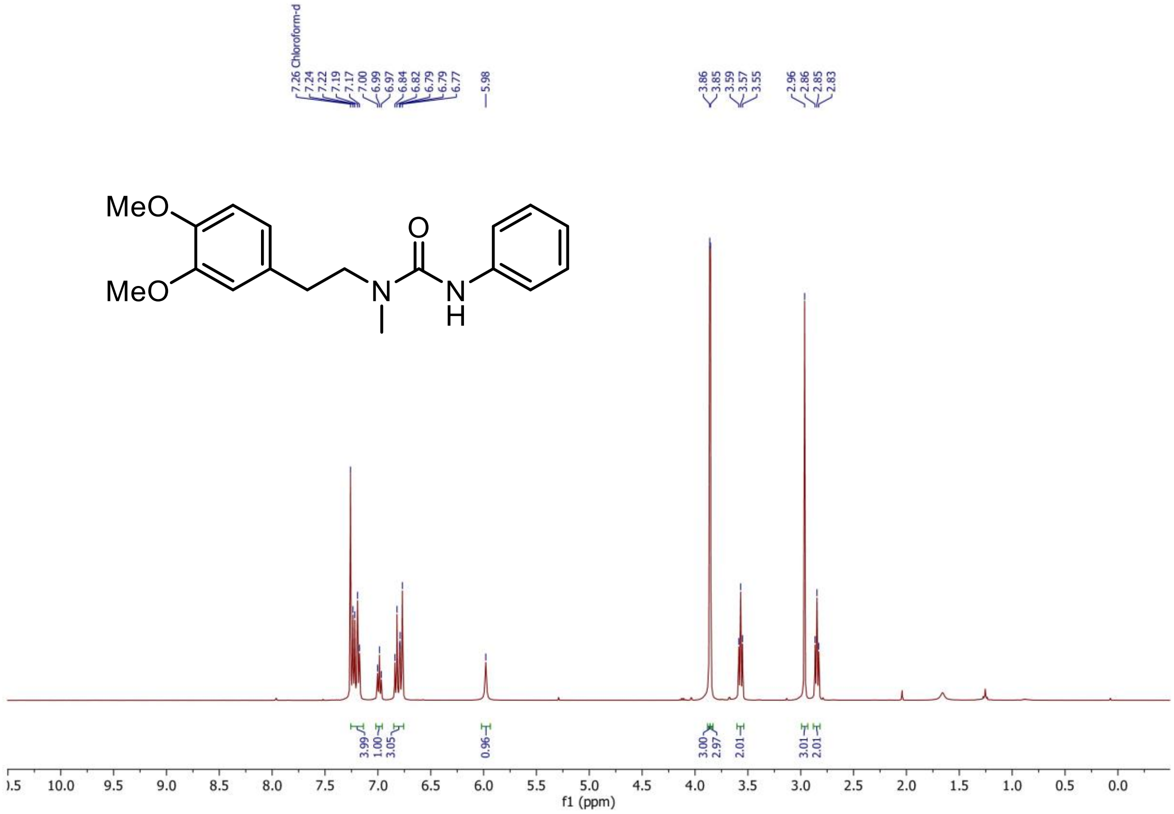

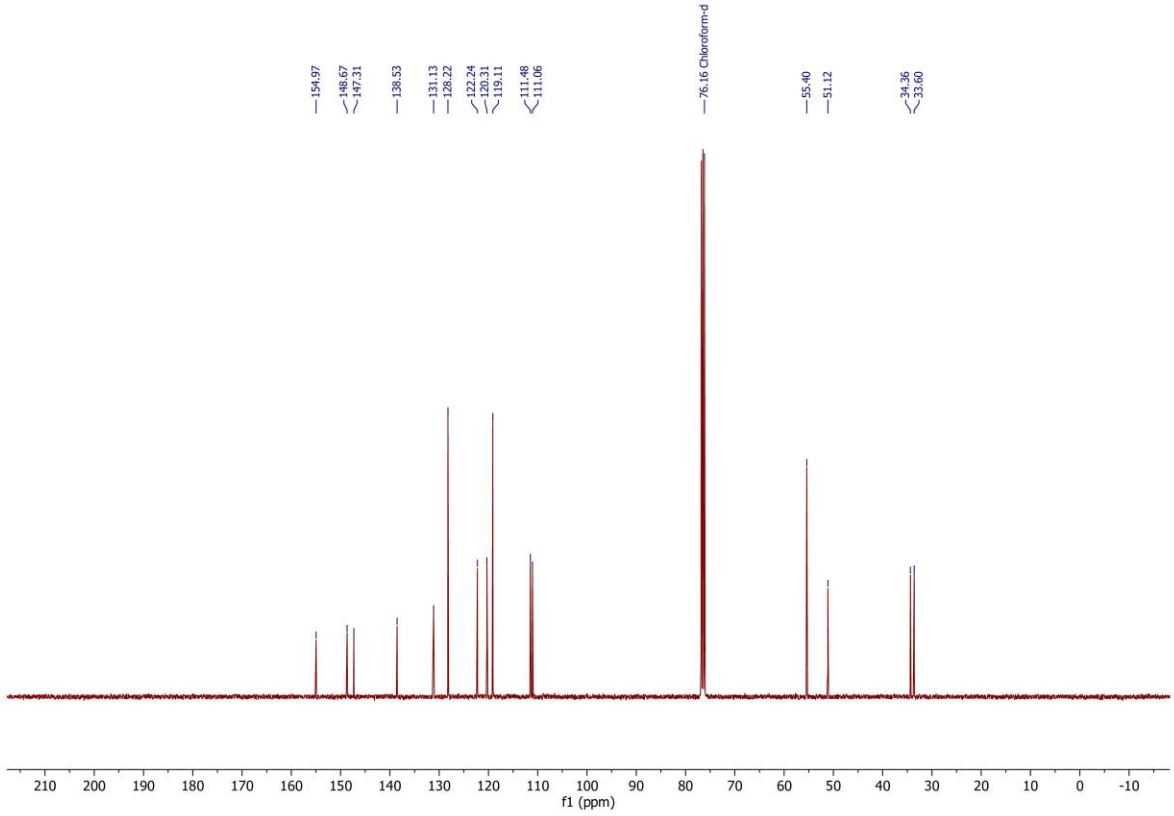

1-(3,4-Dimethoxyphenethyl)-1-methyl-3-phenethylurea (3b)

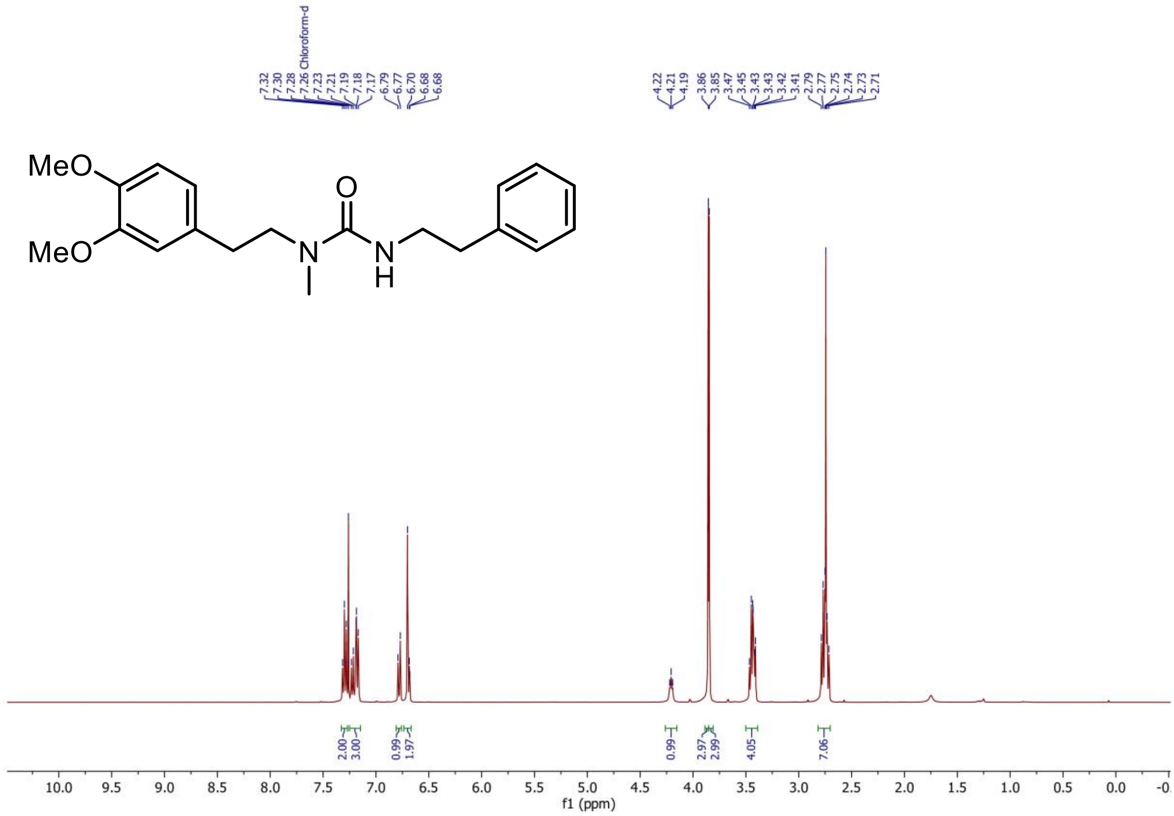

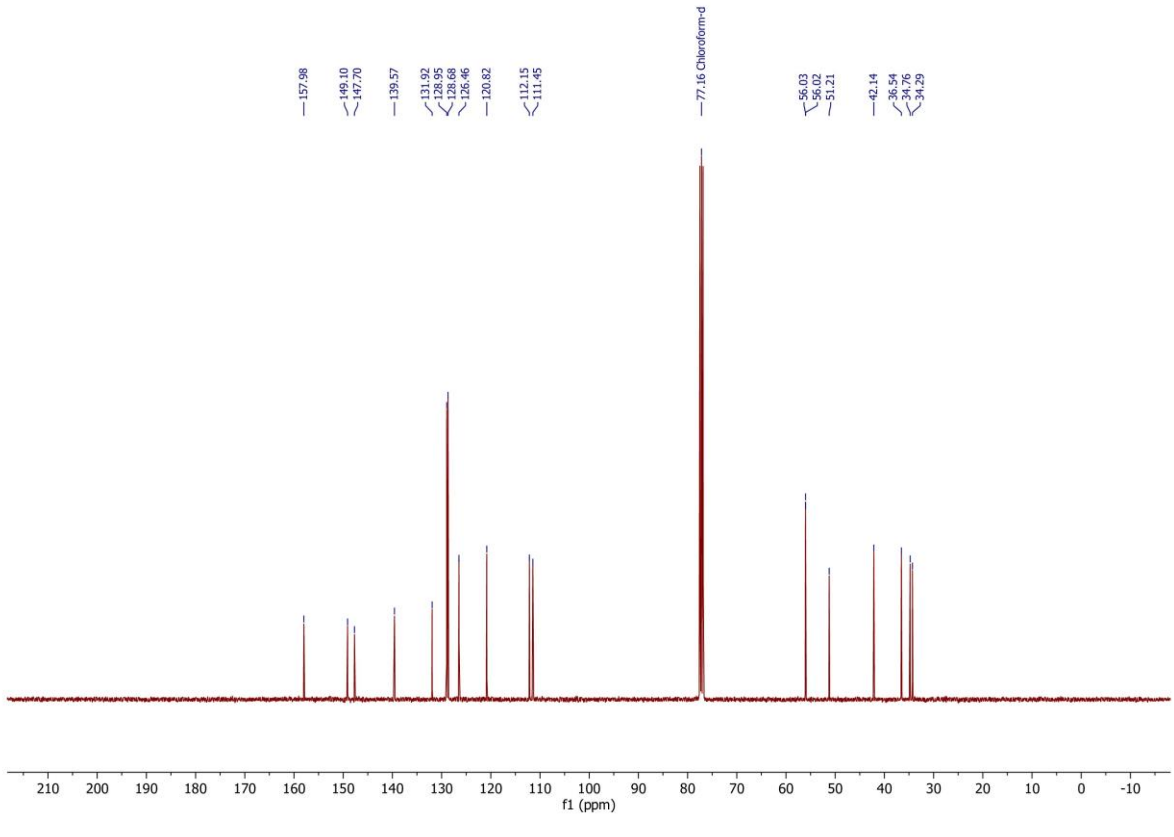

3-Cyclohexyl-1-(3,4-dimethoxyphenethyl)-1-methylurea (3c)

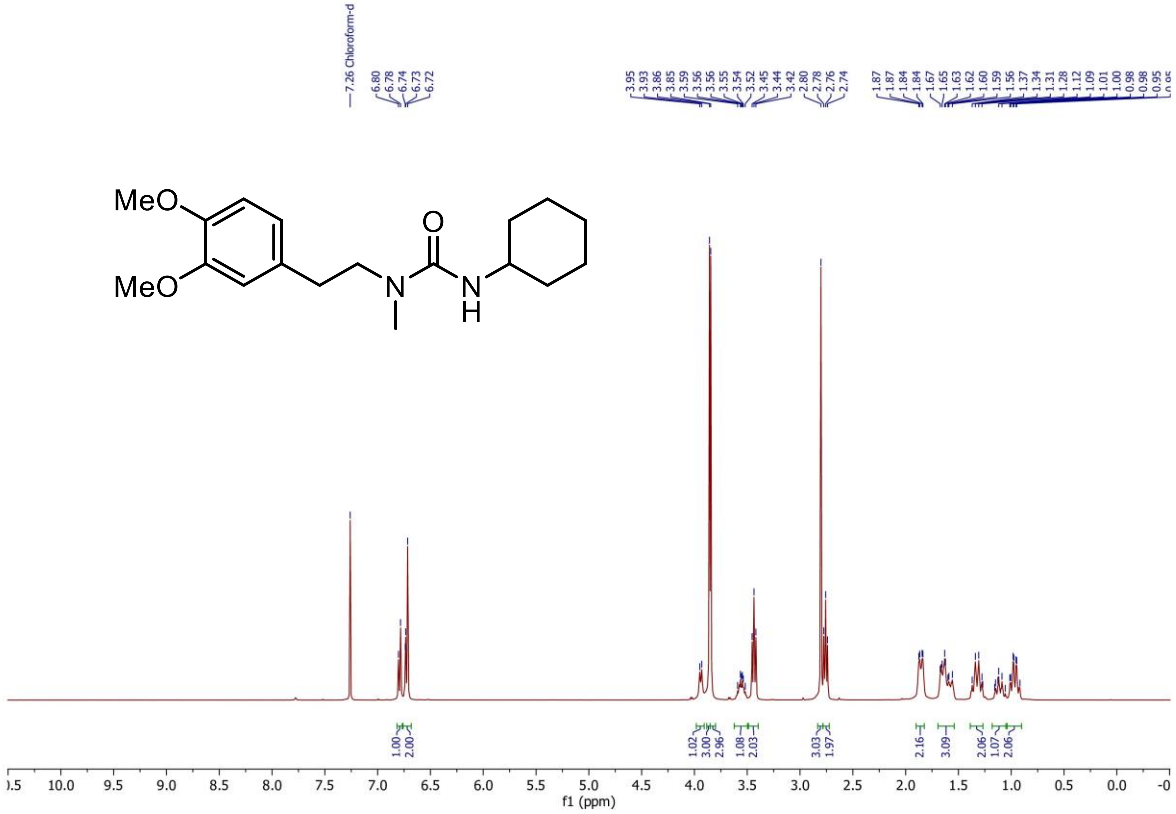

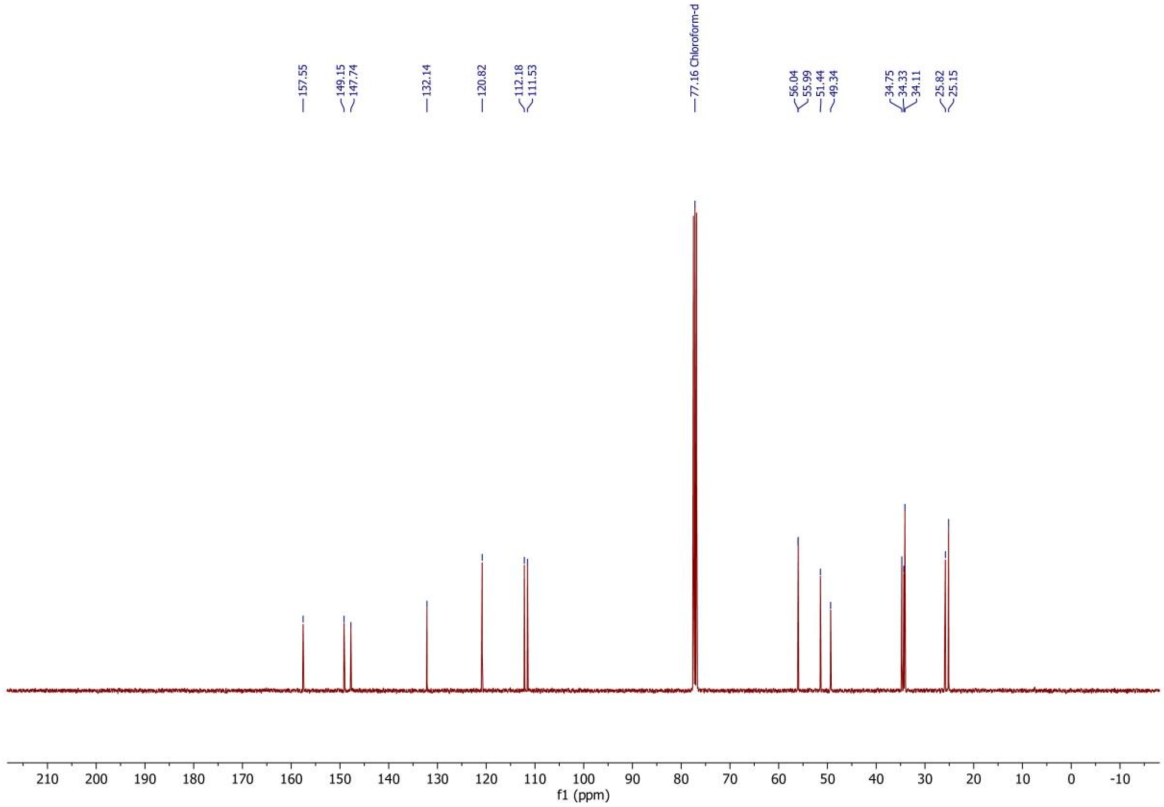

3-(*Tert*-butyl)-1-(3,4-dimethoxyphenethyl)-1-methylurea (3d)

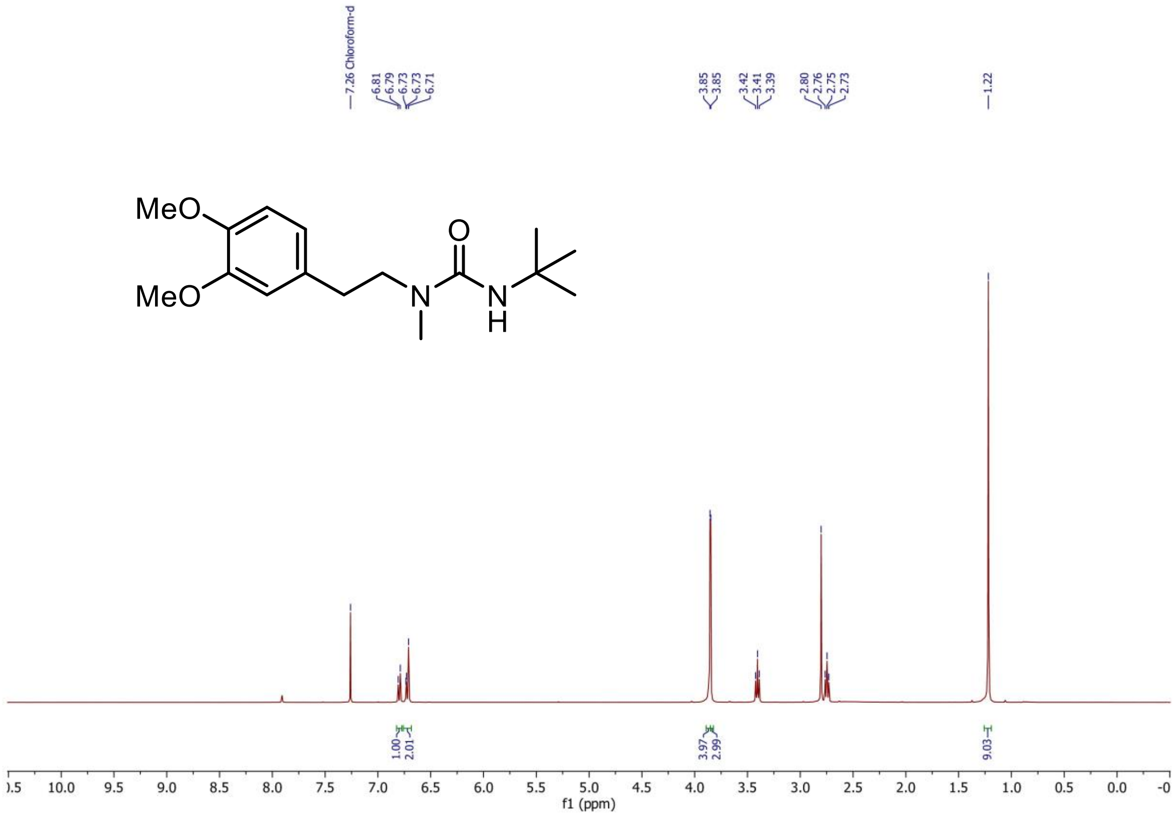

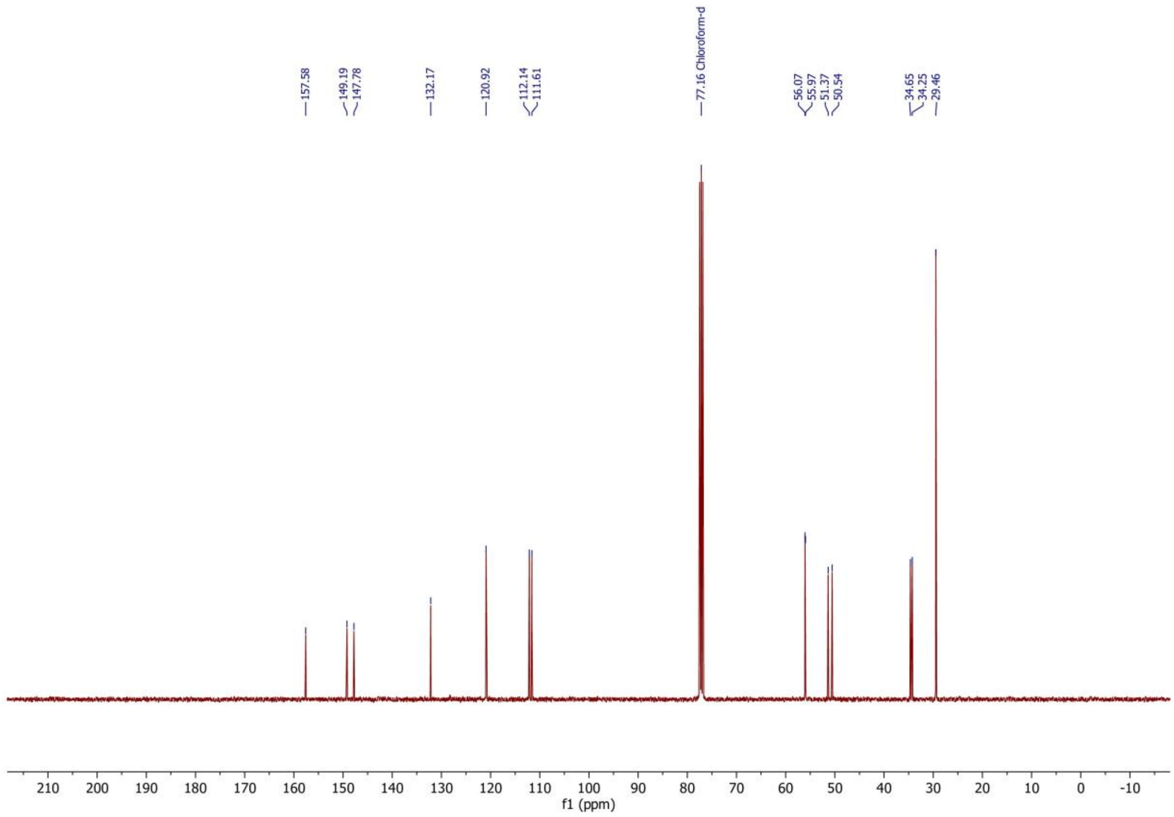

3-Benzyl-1-(3,4-dimethoxyphenethyl)-1-methylurea (3e)

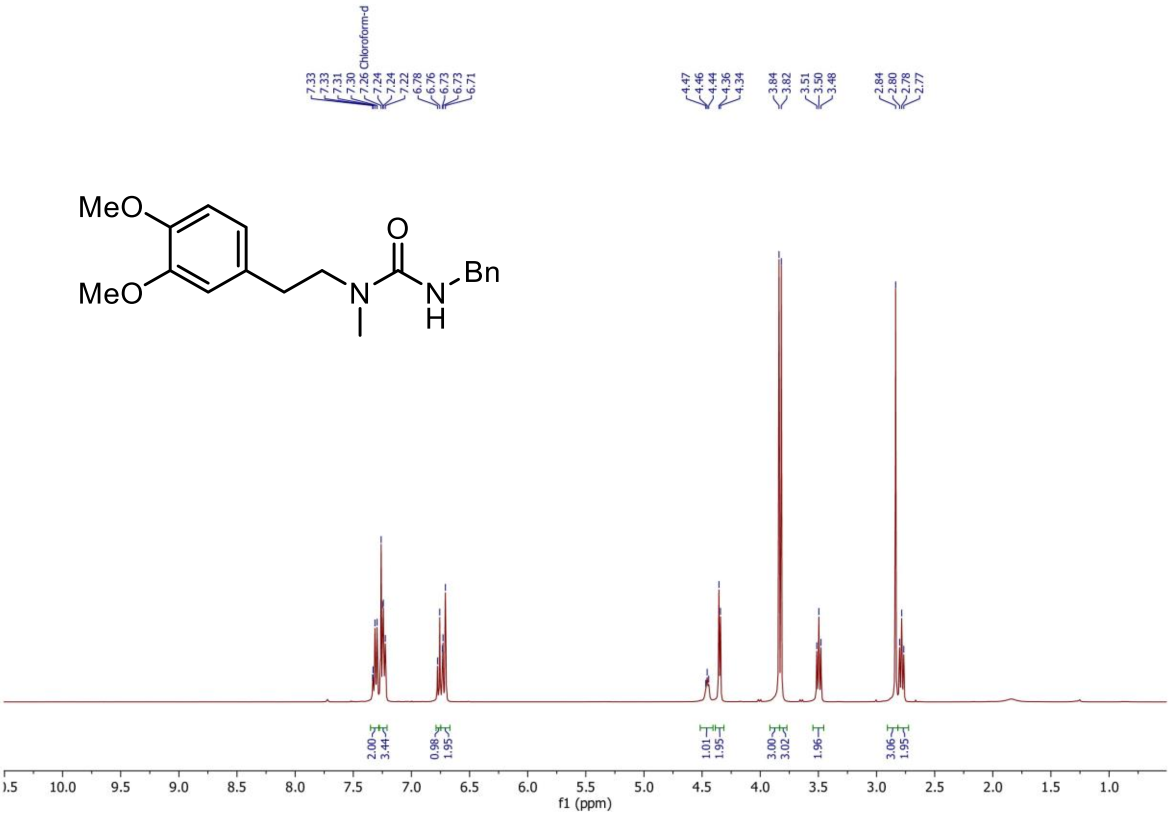

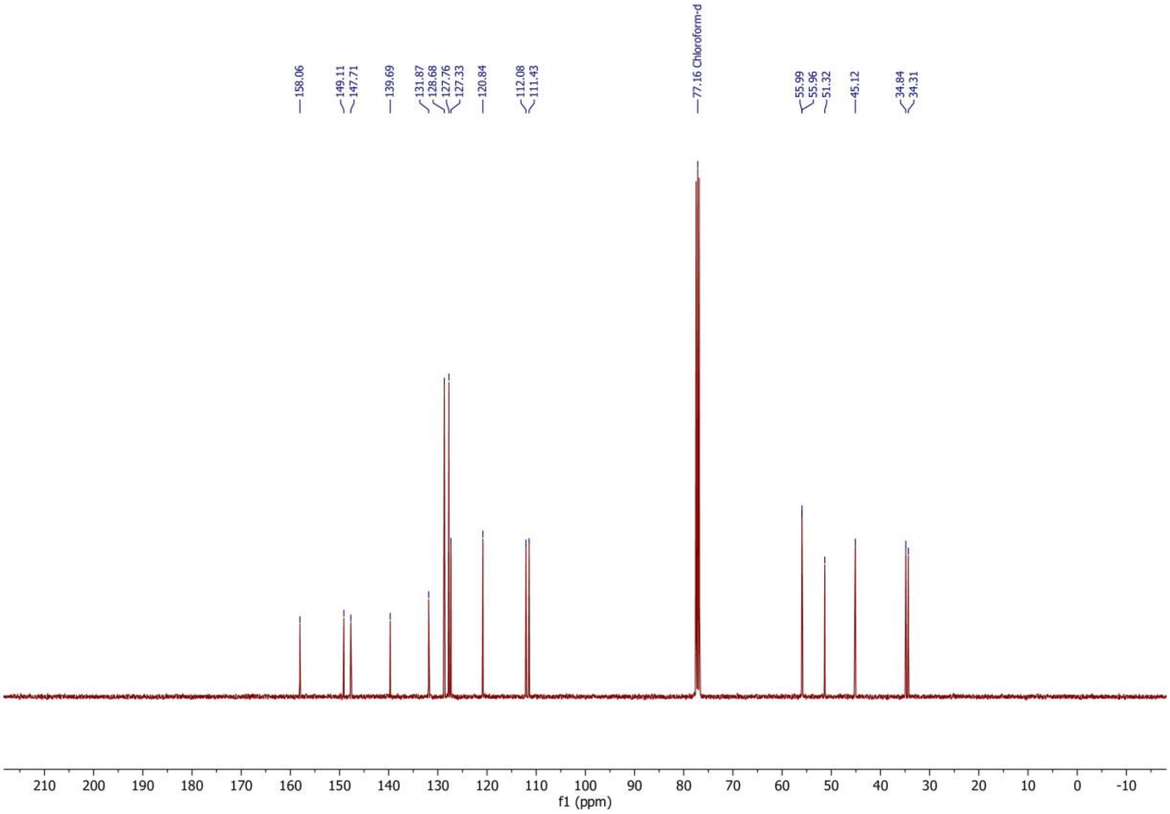

(*E*)-1-(3,4-Dimethoxyphenethyl)-1-methyl-3-styrylurea (3f)

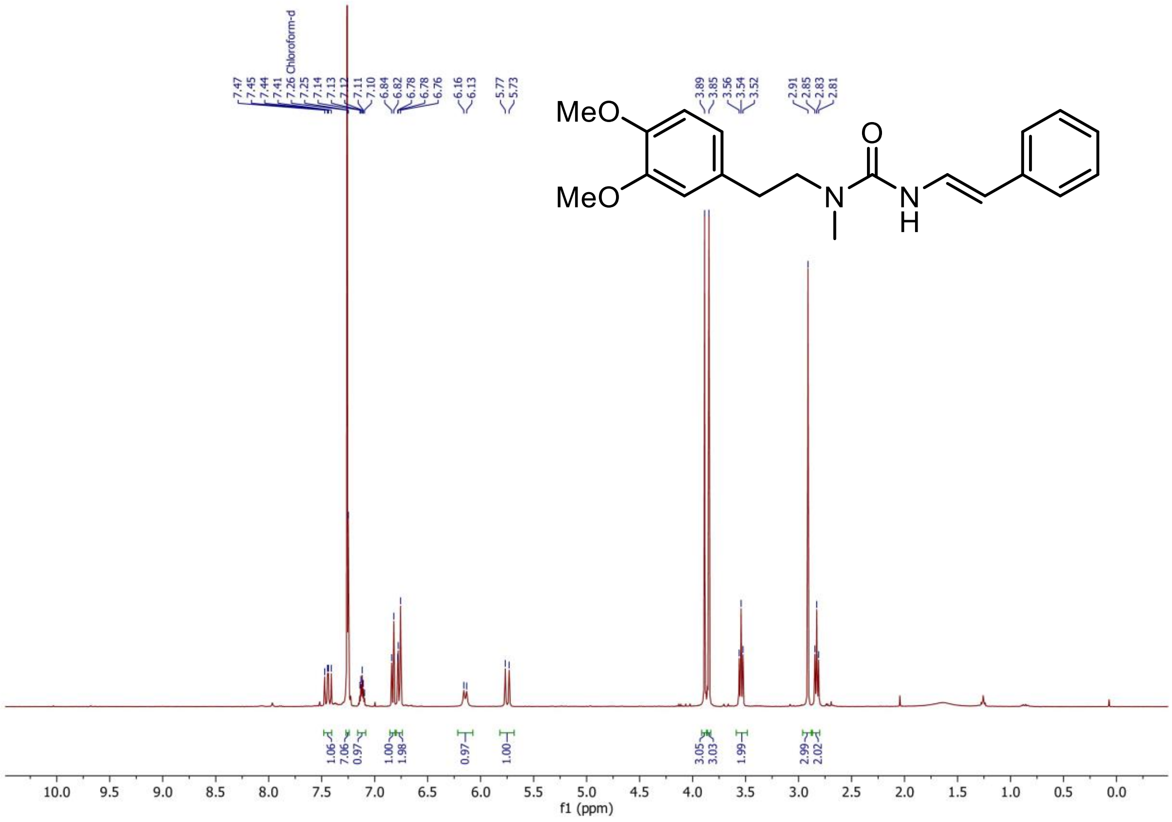

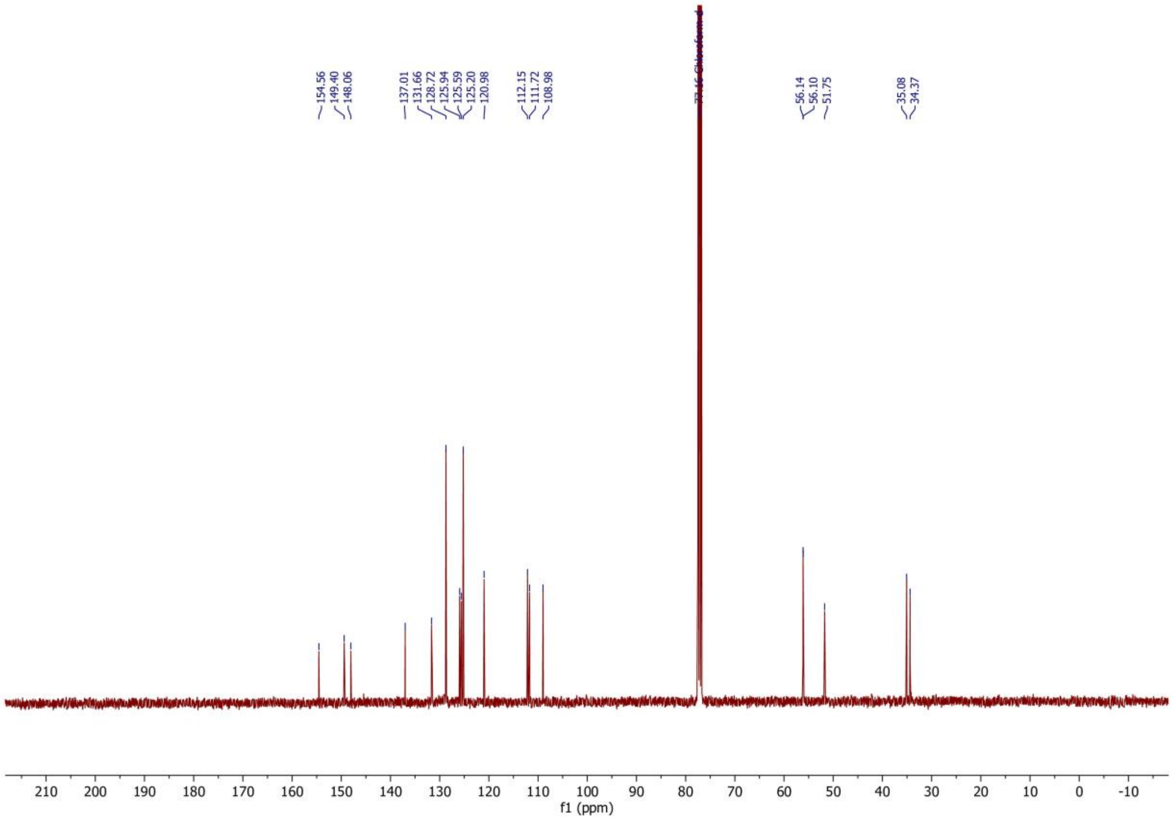

1-(3,4-Dimethoxyphenethyl)-1-methyl-3-(quinolin-2-yl)urea (3g)

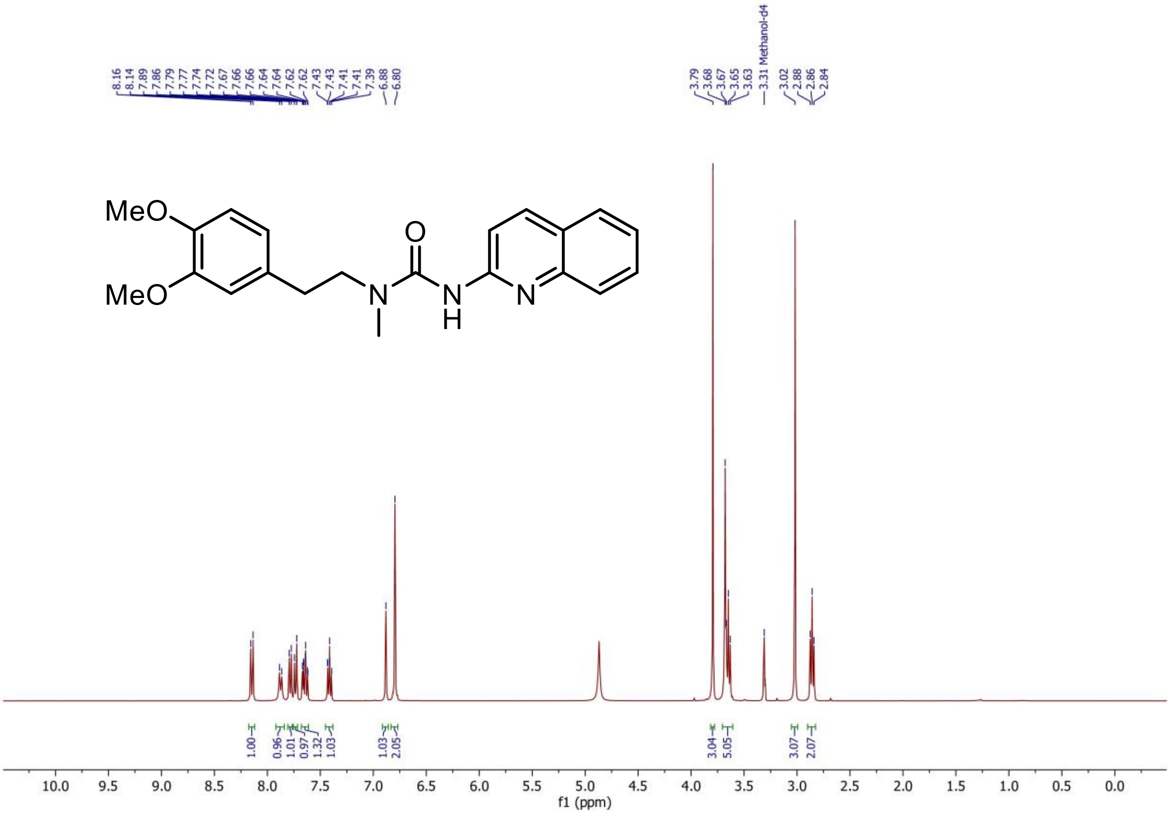

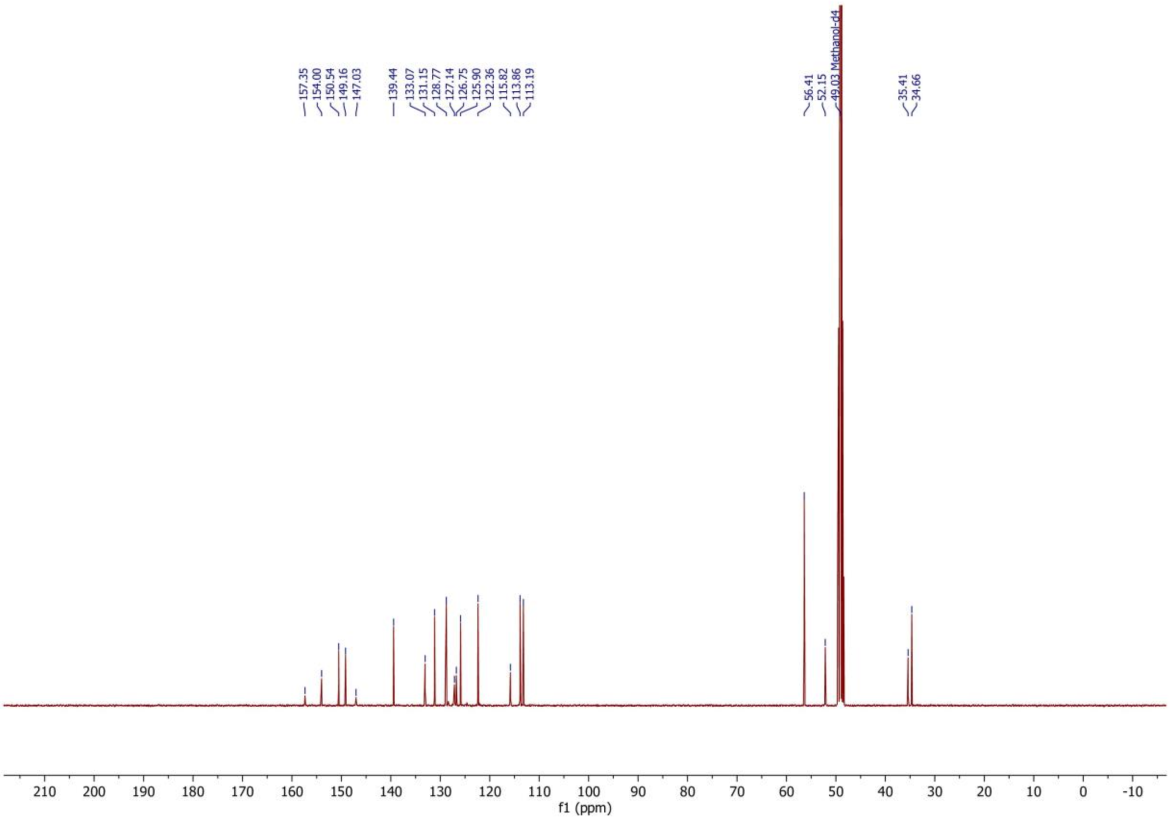

3-(2-(3-Cyano-4-isobutoxyphenyl)-4-methylthiazol-5-yl)-1-(3,4-dimethoxyphenethyl)-1-methylurea (3h)

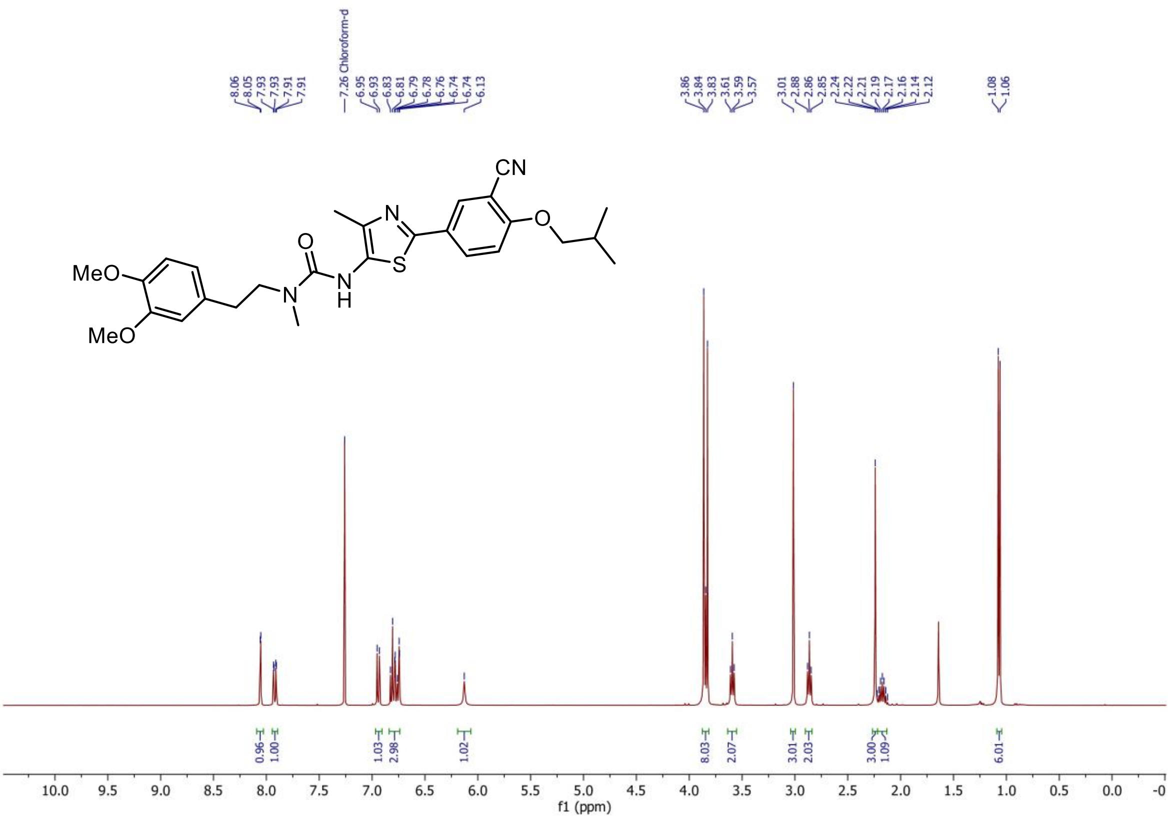

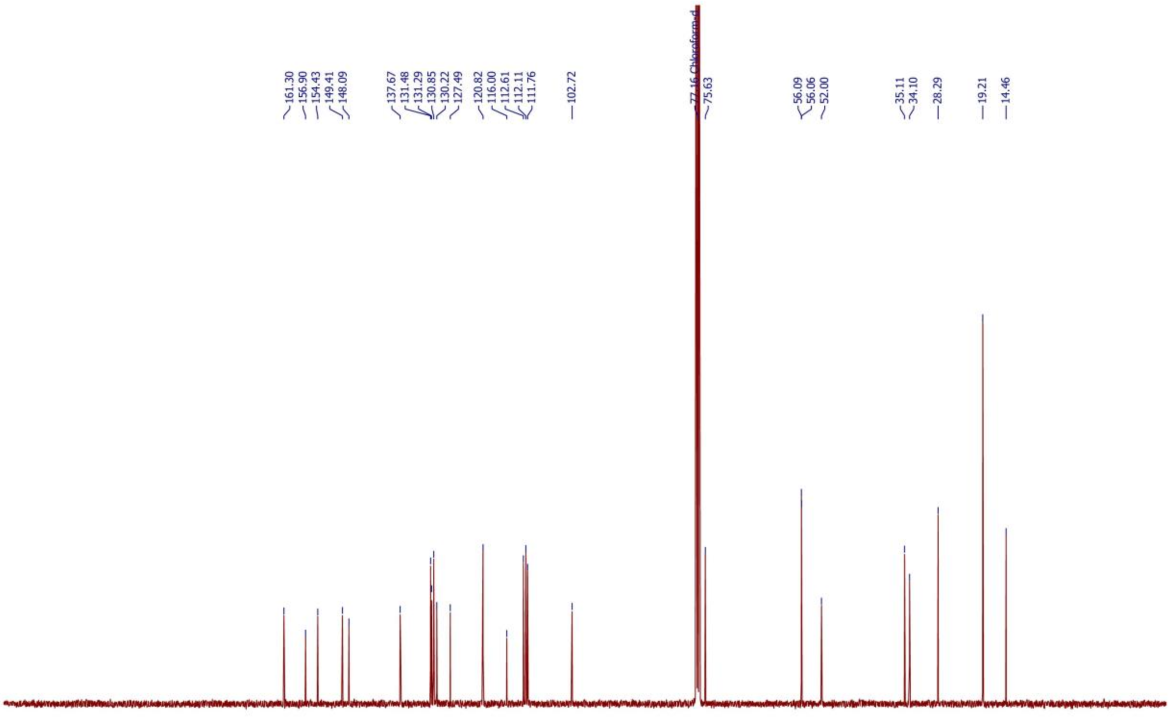

(*E*)-1-(3,4-dimethoxyphenethyl)-3-(5-(4-hydroxy-6-methoxy-7-methyl-3-oxo-1,3-dihydroisobenzofuran-5-yl)-3-methylpent-3-en-1-yl)-1-methylurea (3i)

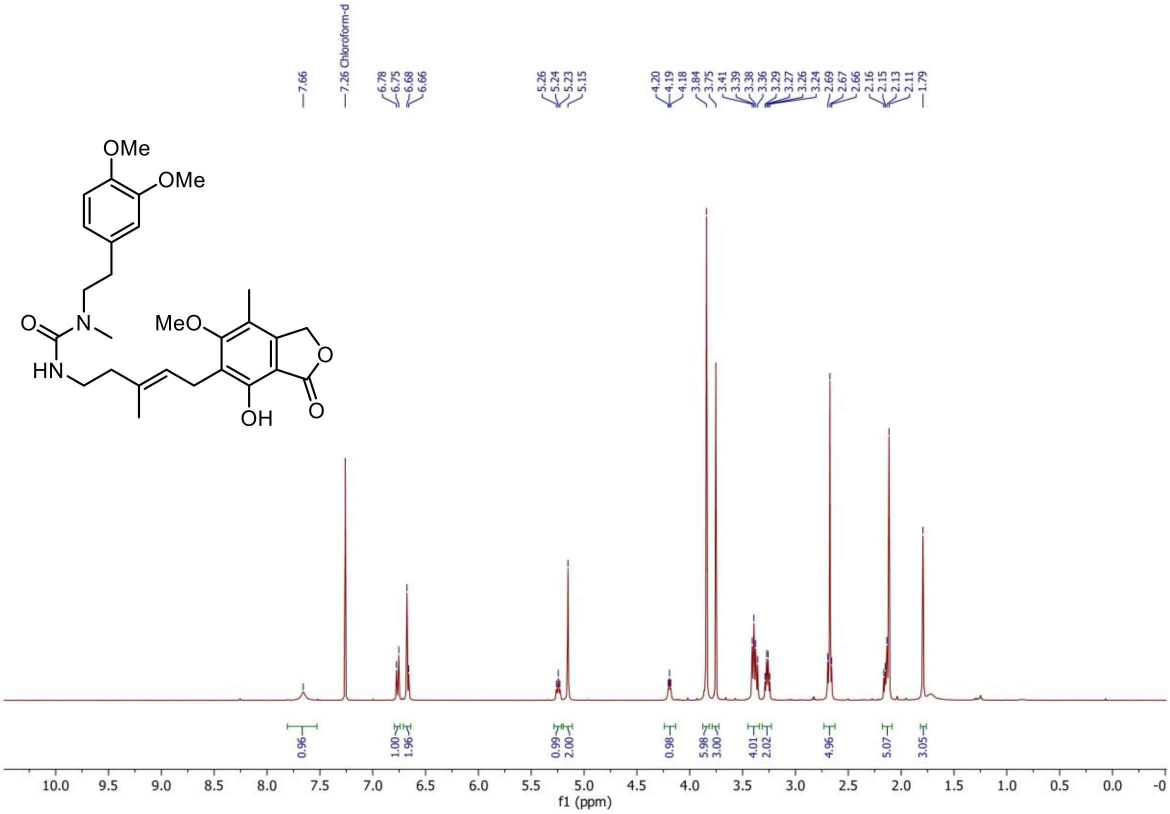

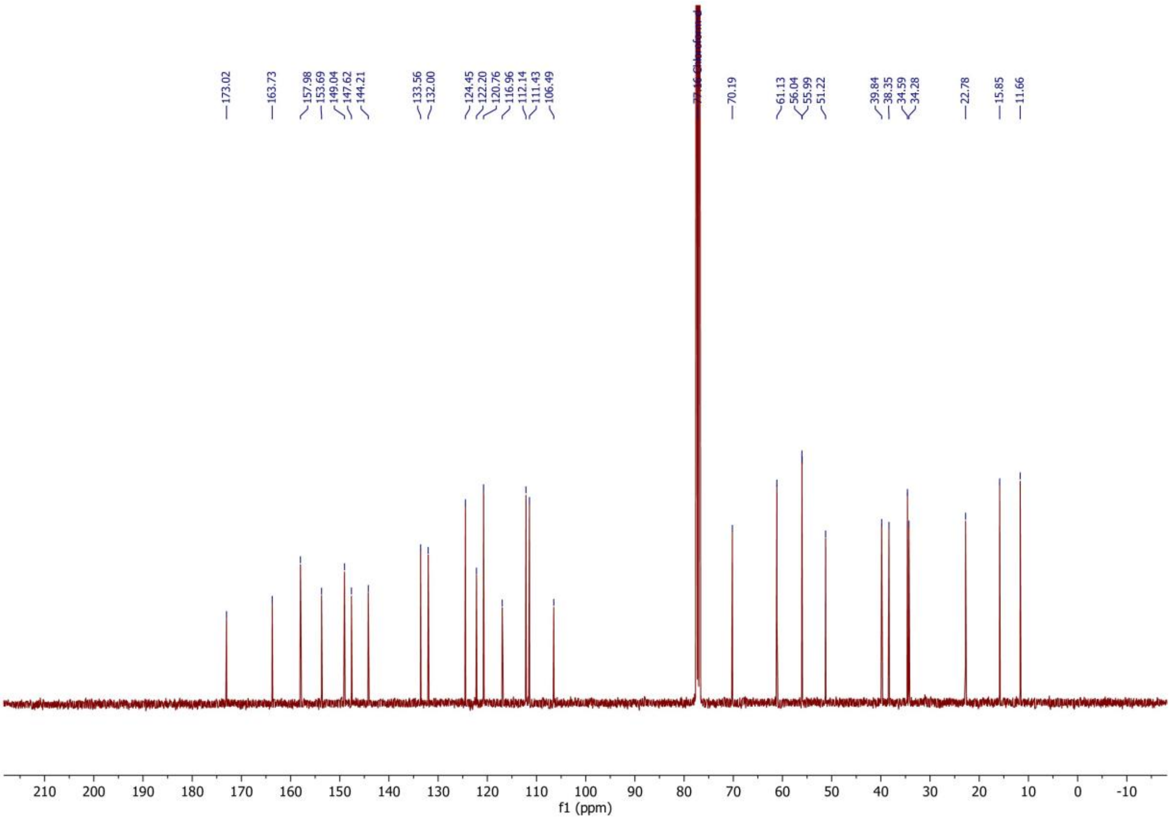

1-Phenethyl-3-phenylurea (7a)

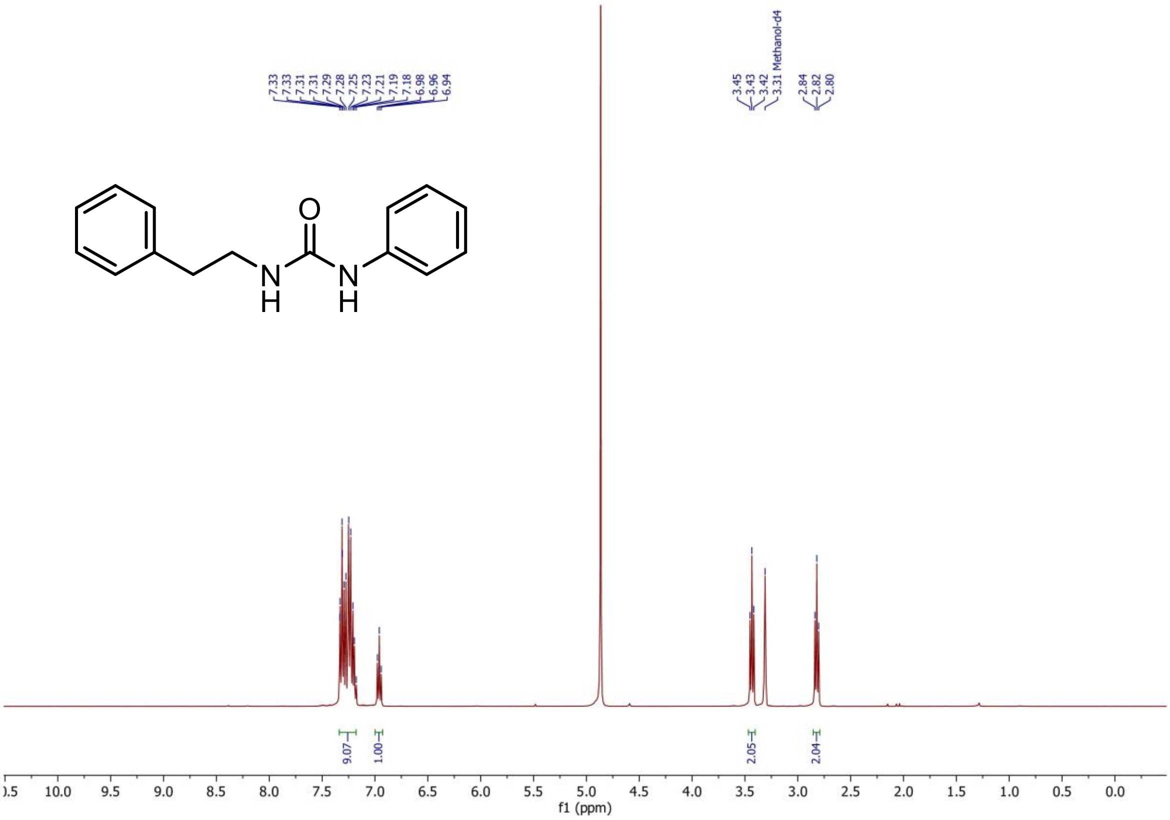

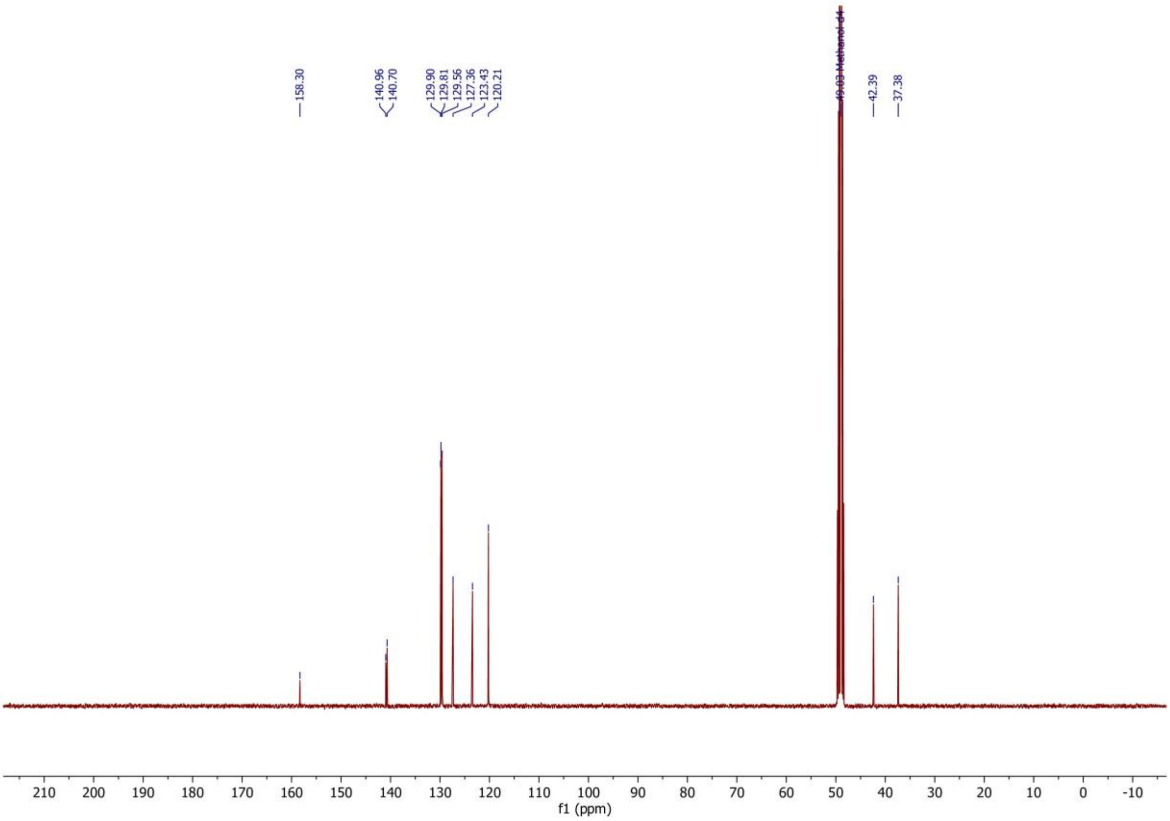

1-(2-(1*H*-Indol-3-yl)ethyl)-3-phenylurea (7b)

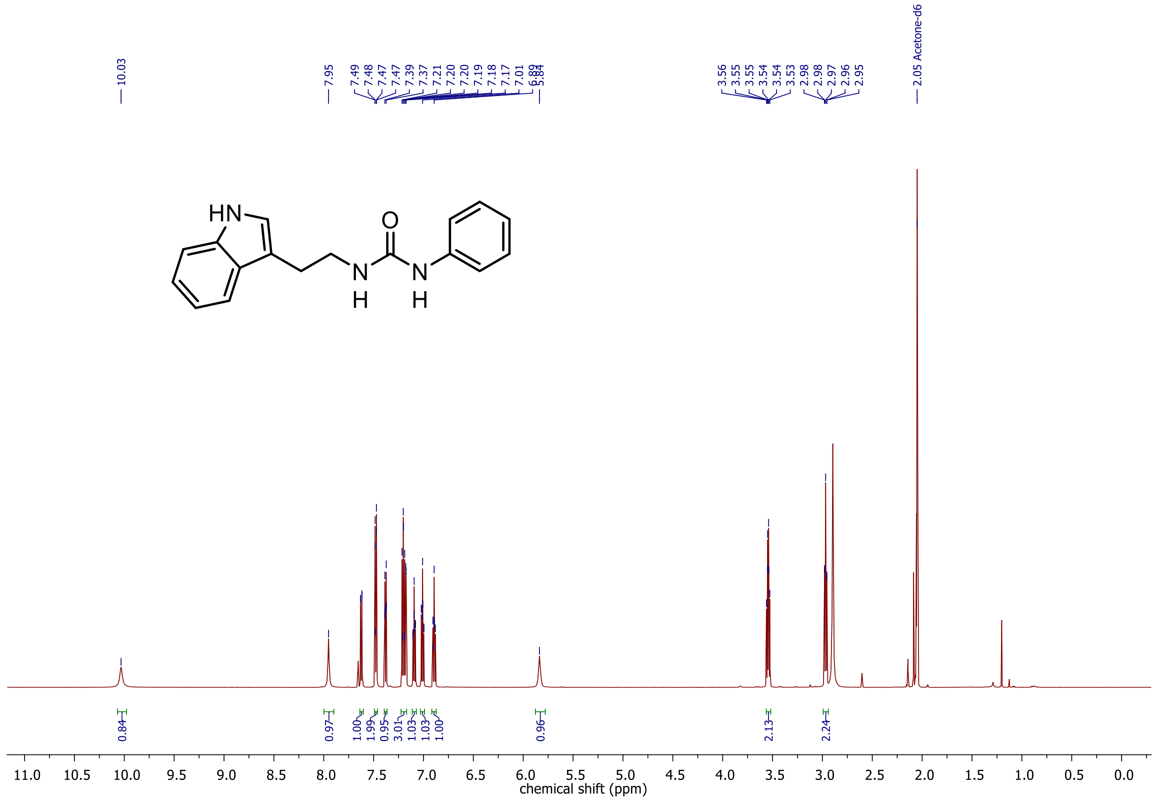

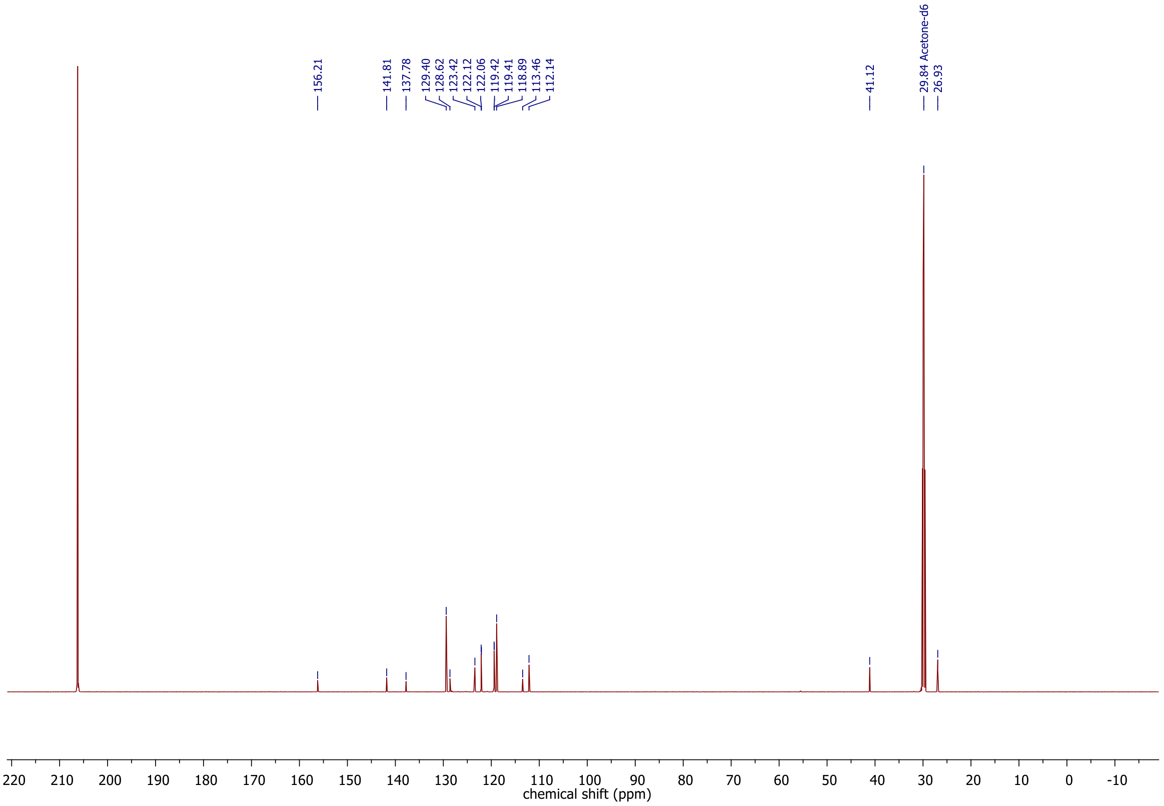

Tert-butyl (3-(3-phenylureido)propyl)carbamate (7c)

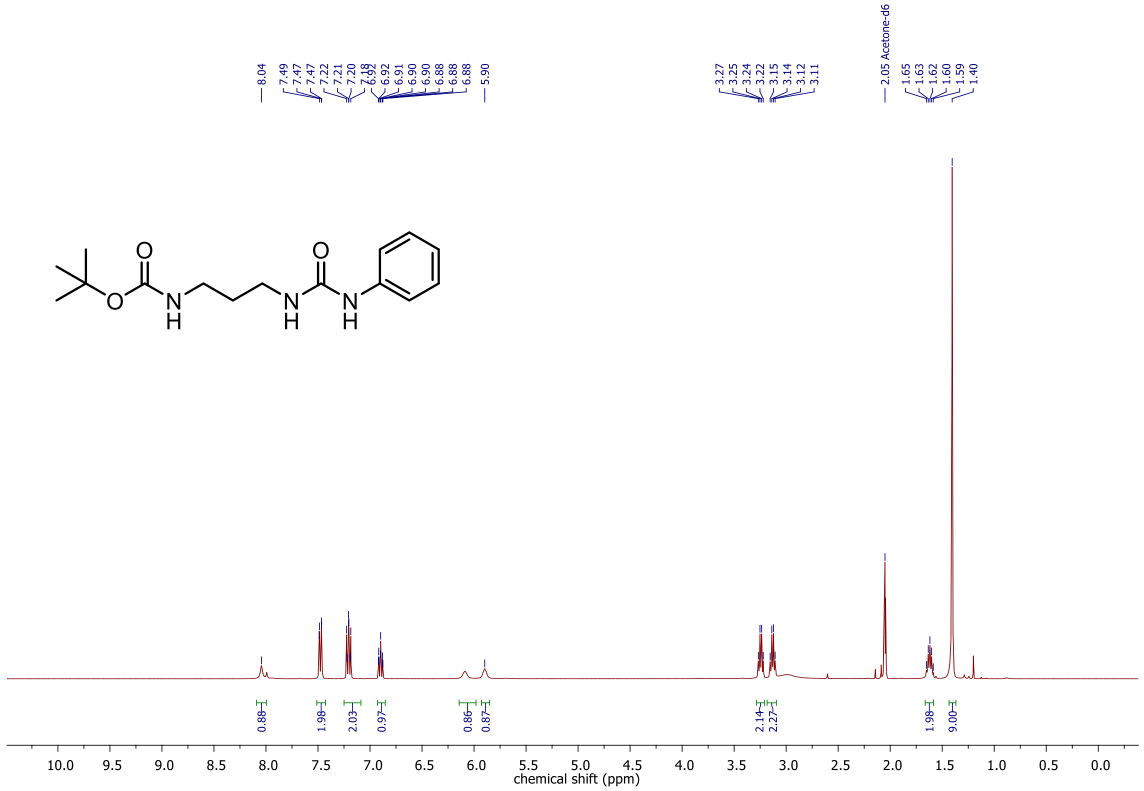

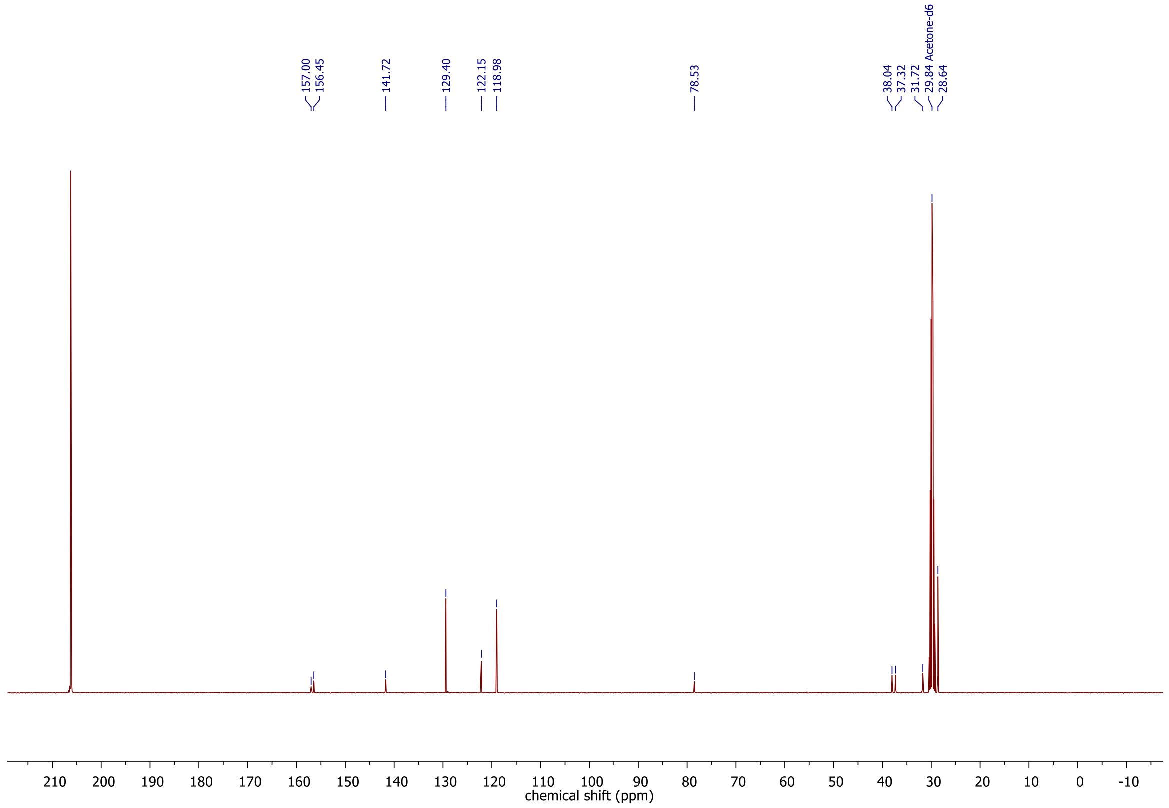

1-(Furan-2-ylmethyl)-3-phenylurea (7d)

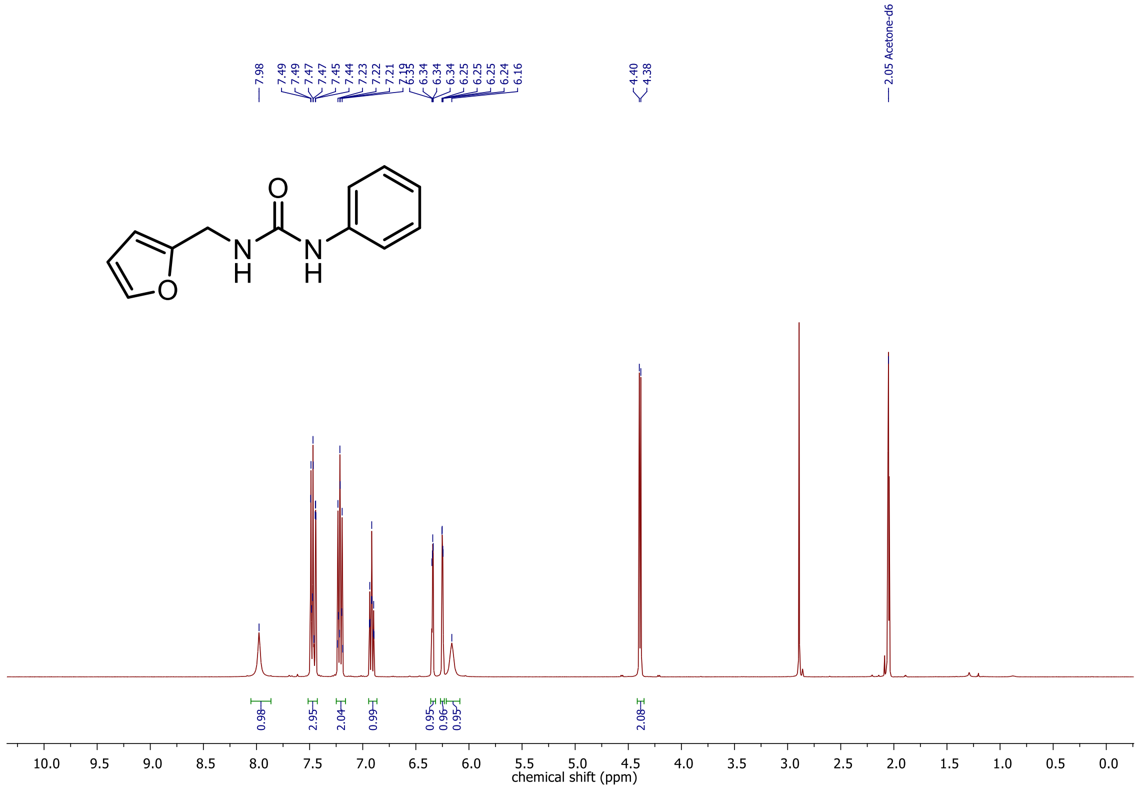

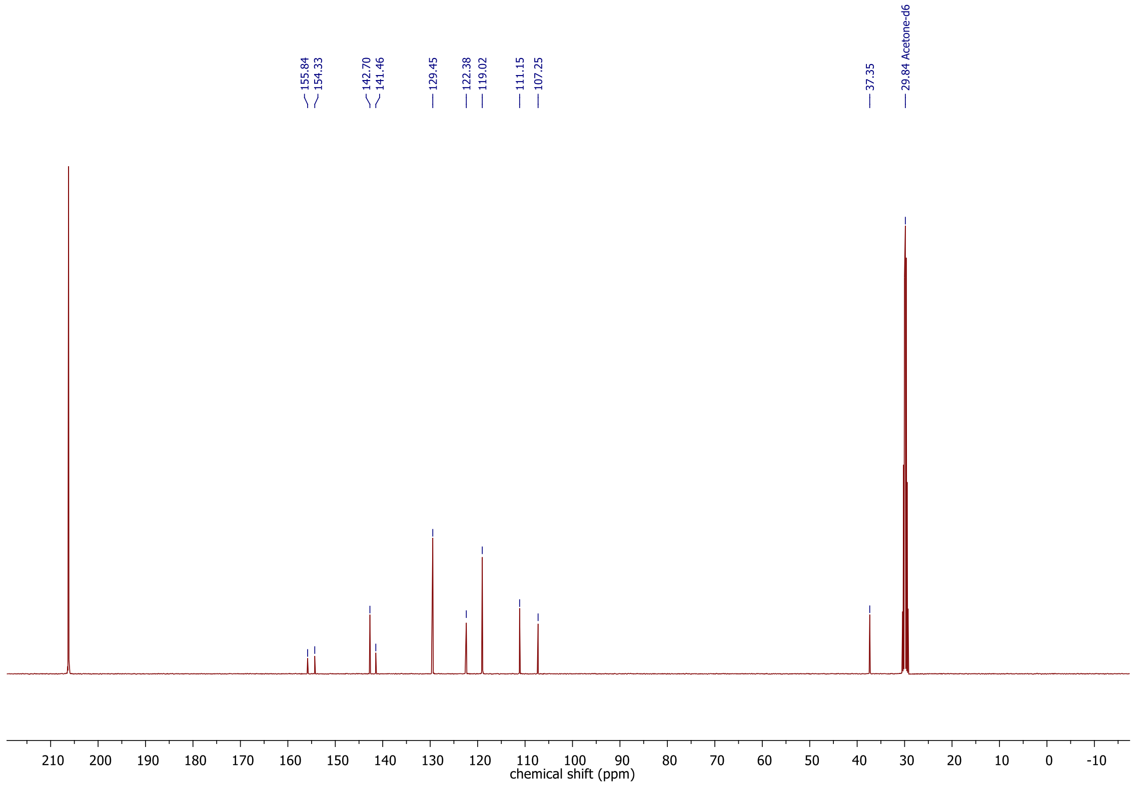

1-Phenyl-3-(pyridin-2-ylmethyl)urea (7e)

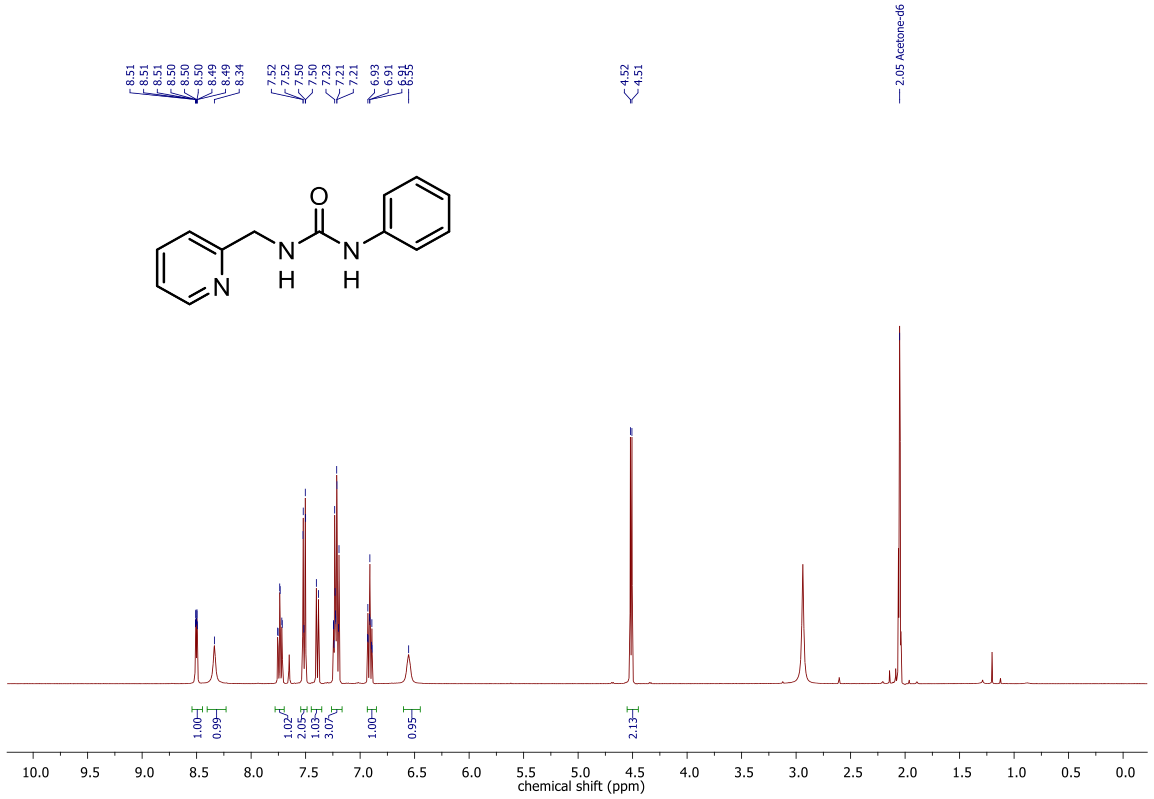

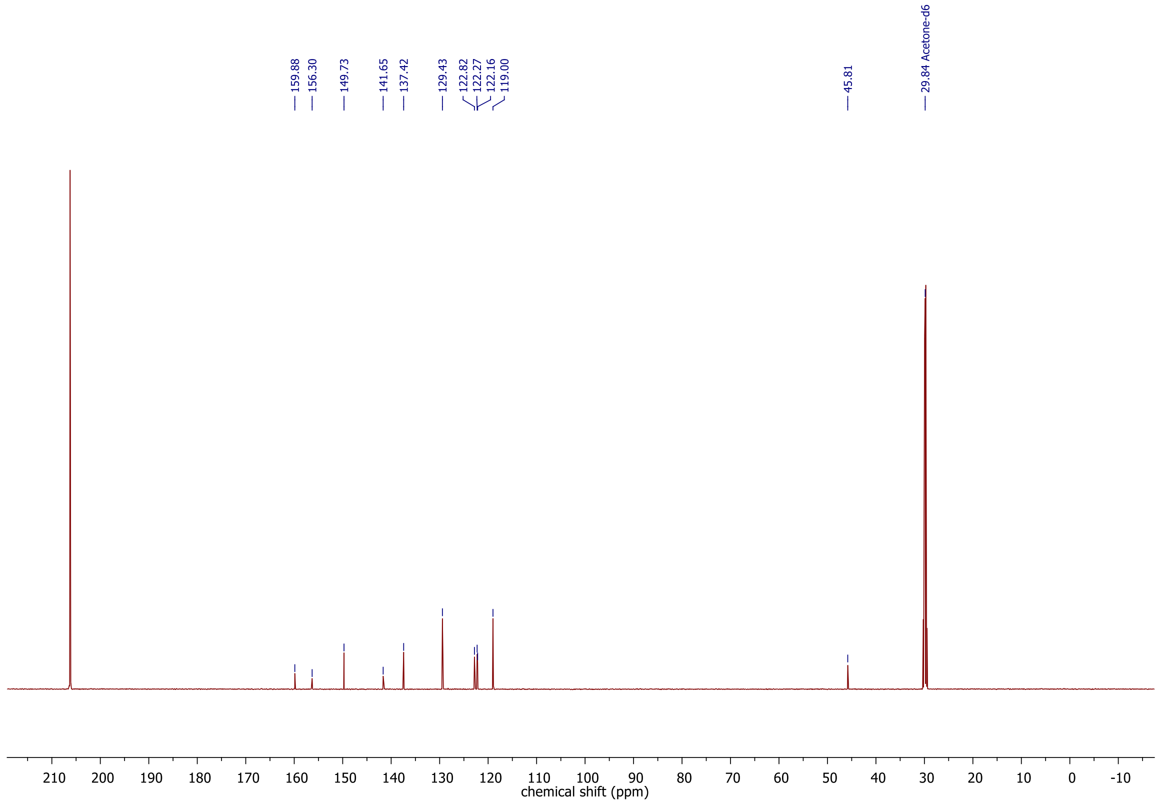

(*R*)-1-(1-(Naphthalen-1-yl)ethyl)-3-phenylurea (7f)

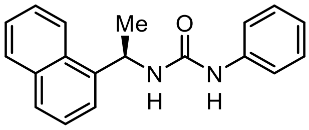

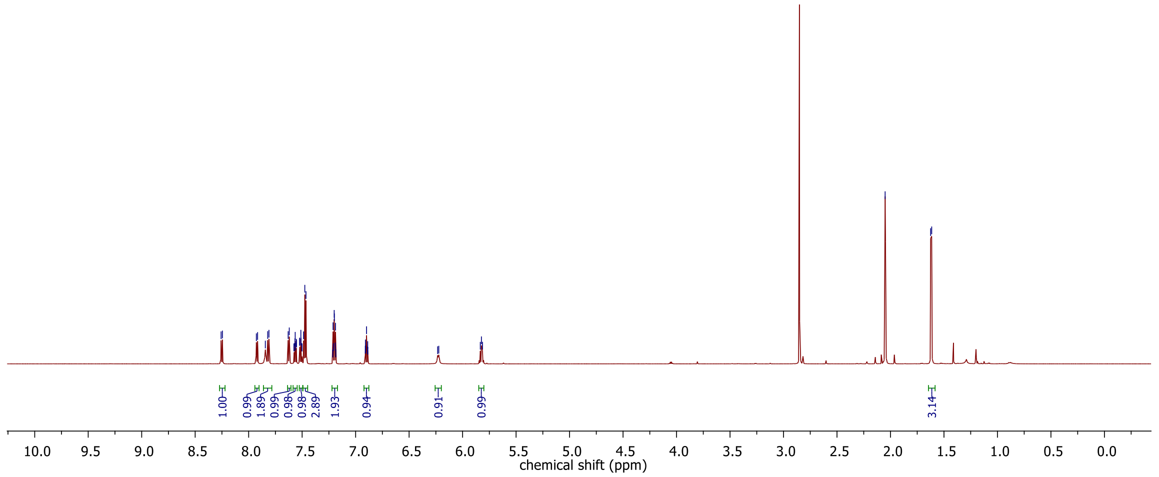

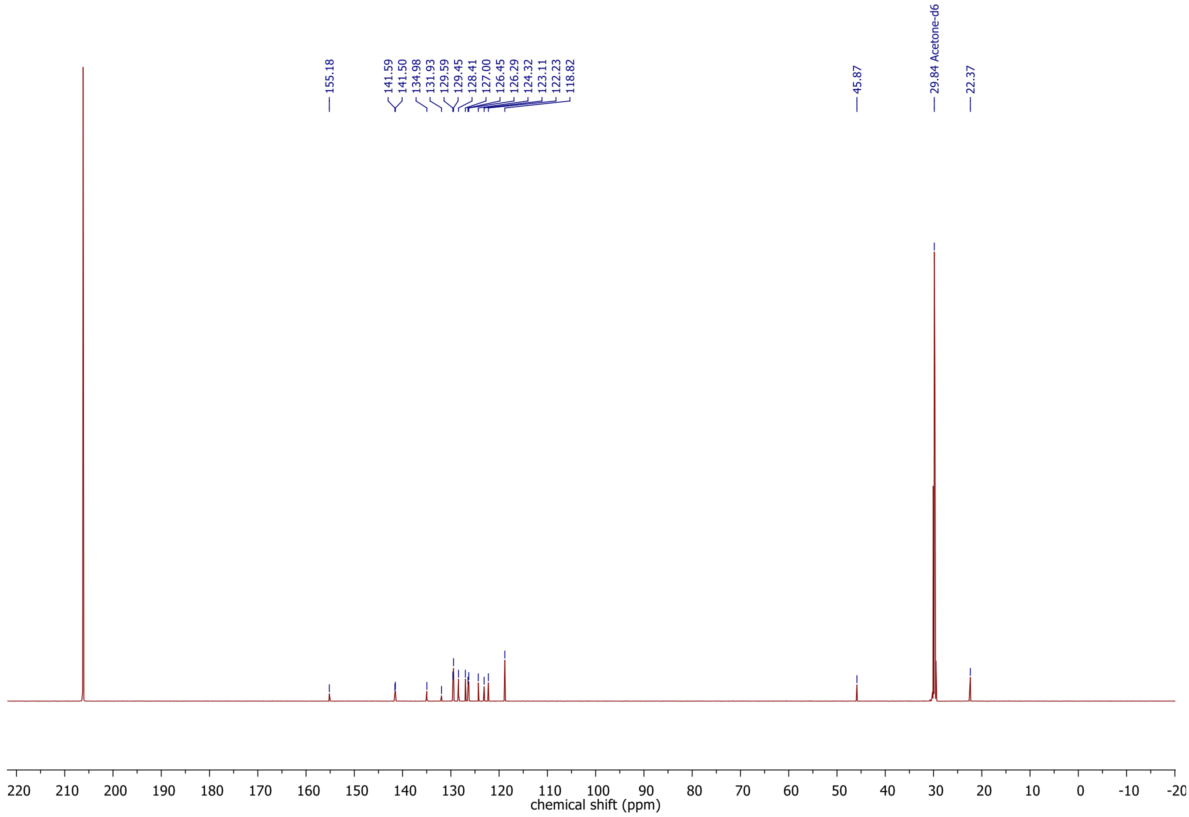

Benzyl (phenylcarbamoyl)-*L*-serinate (7g)

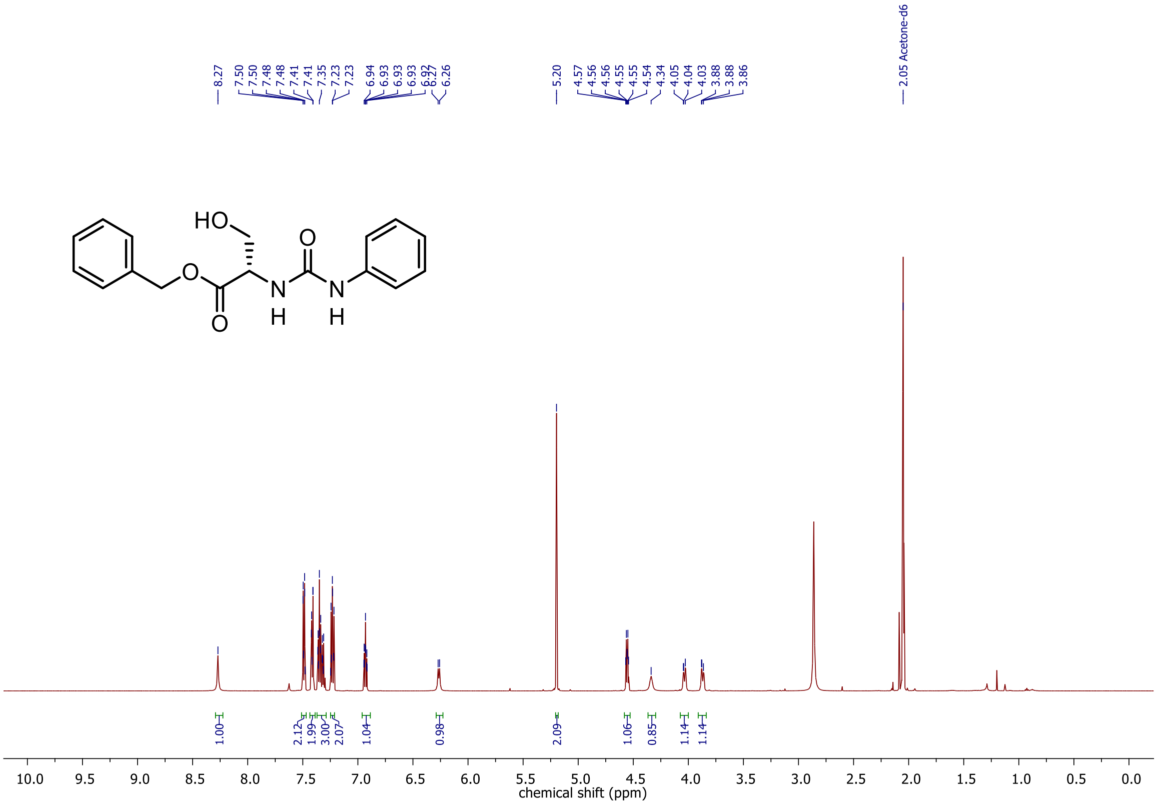

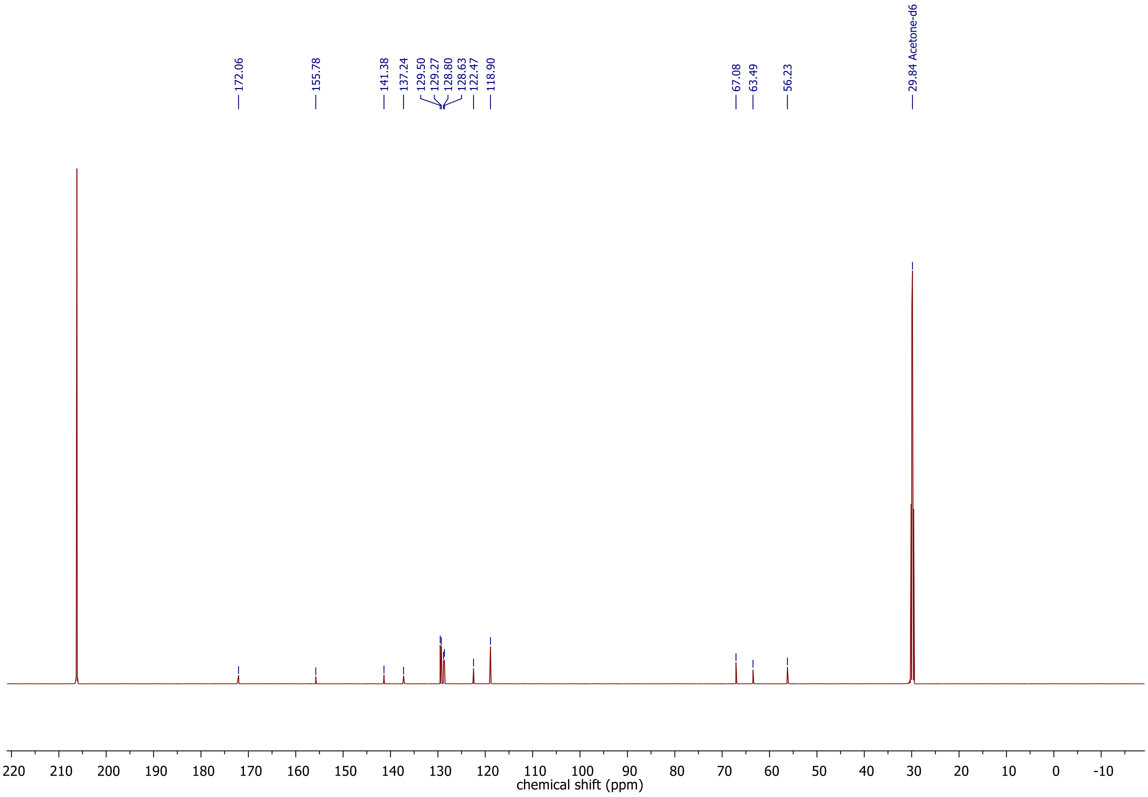

4-Bromo-*N*-phenylpiperidine-1-carboxamide (7h)

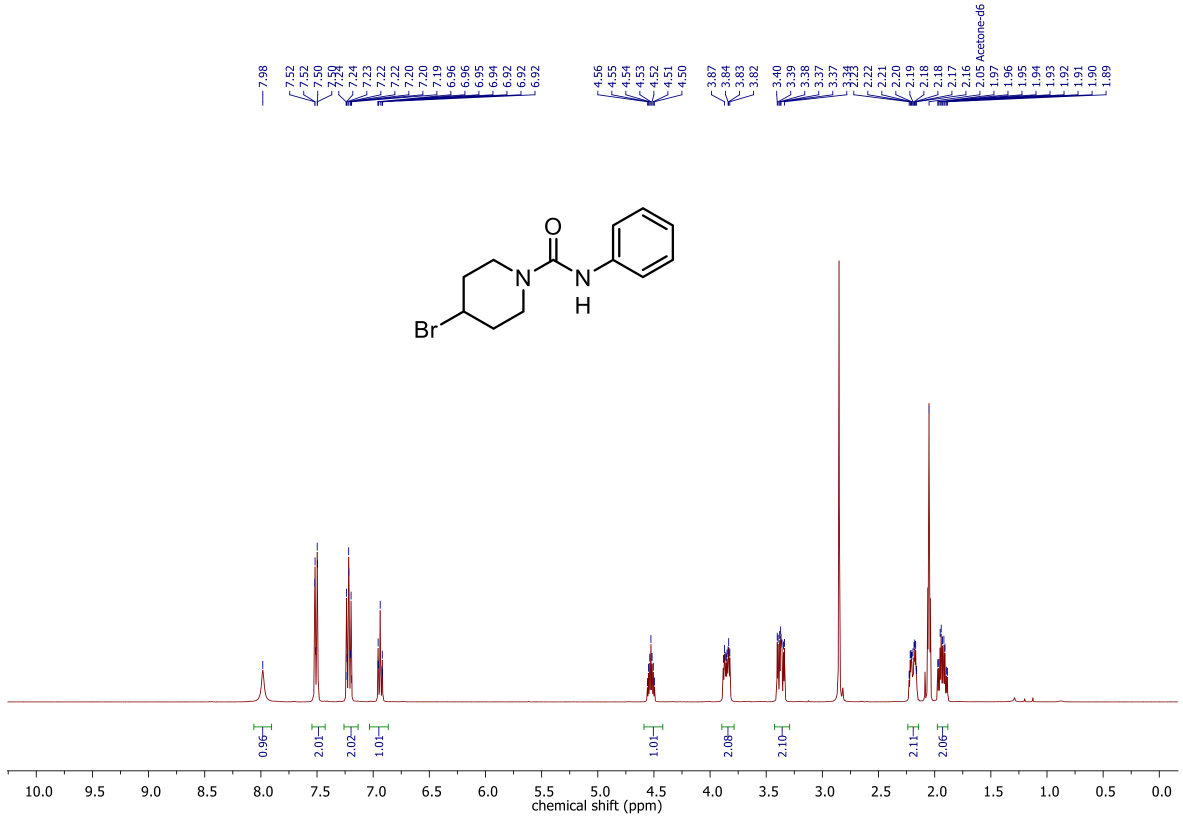

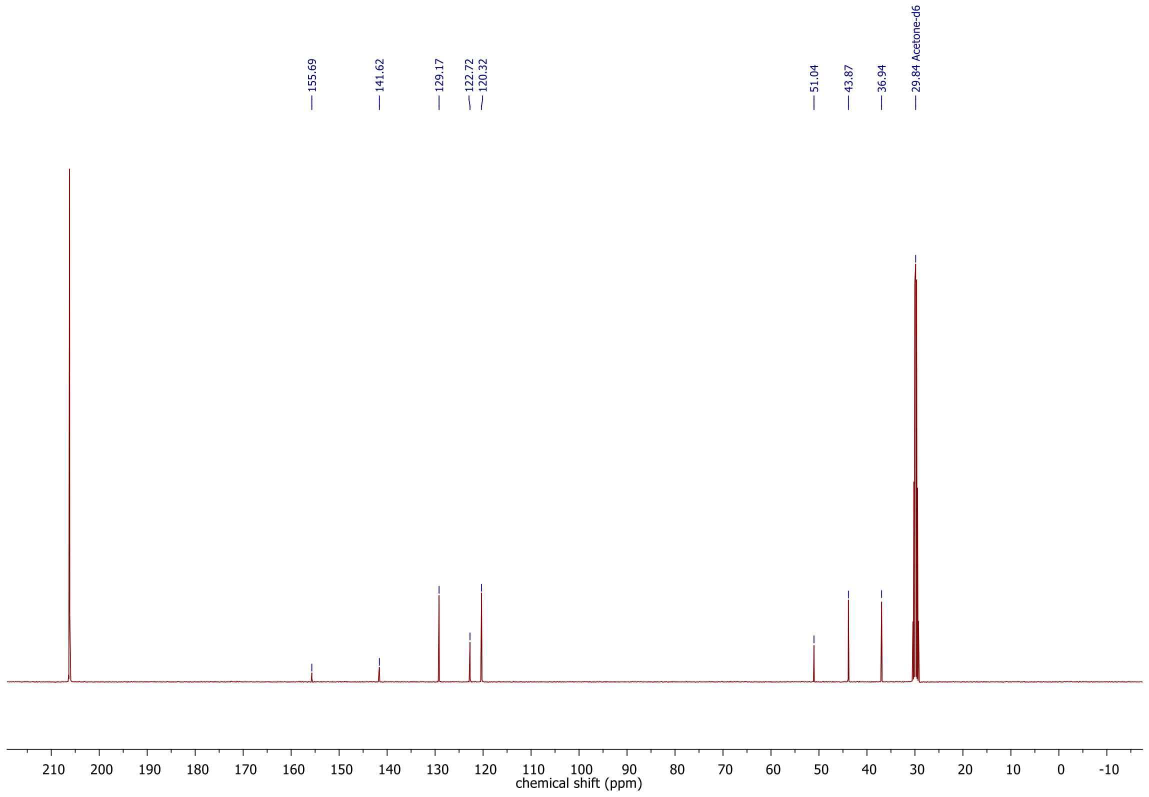

_N_-3-Oxo-_N_-phenyl-8-azabicyclo[3.2.1]octane-8-carboxamide (7i)

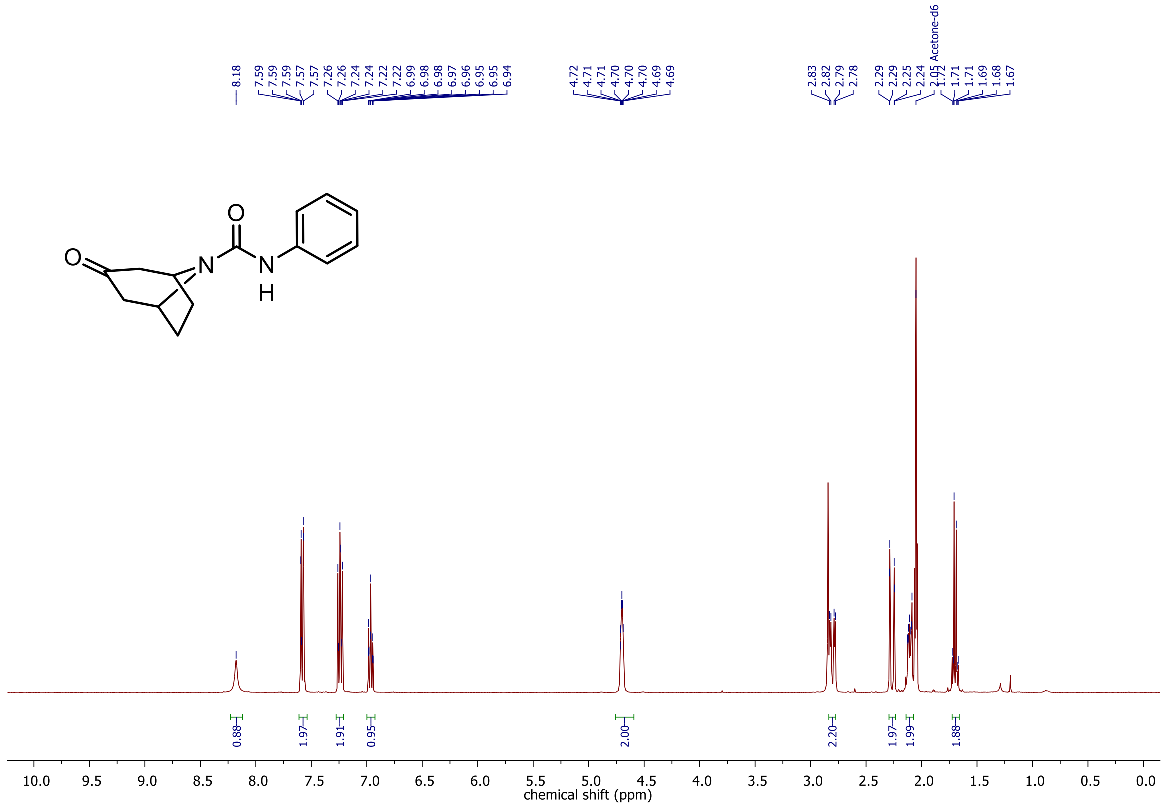

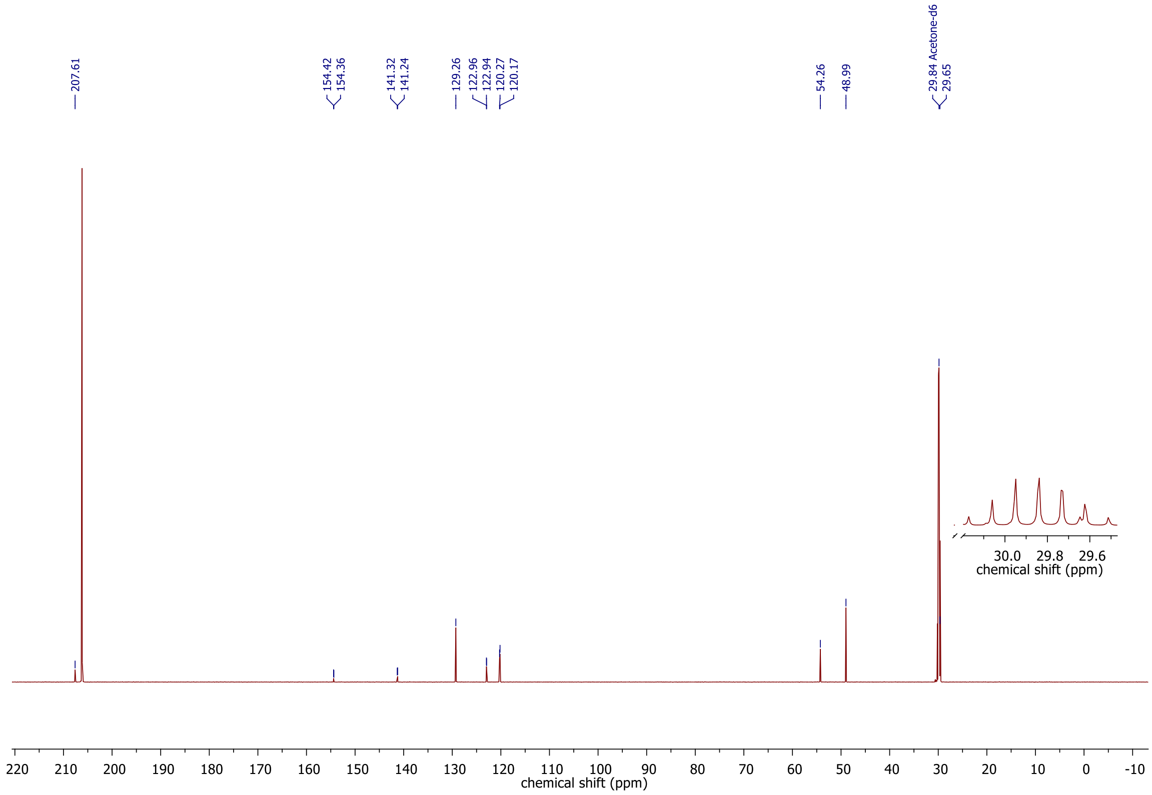

Phenyl-4-(4,4,5,5-tetramethyl-1,3,2-dioxaborolan-2-yl)piperidine-1-carboxamide (7j)

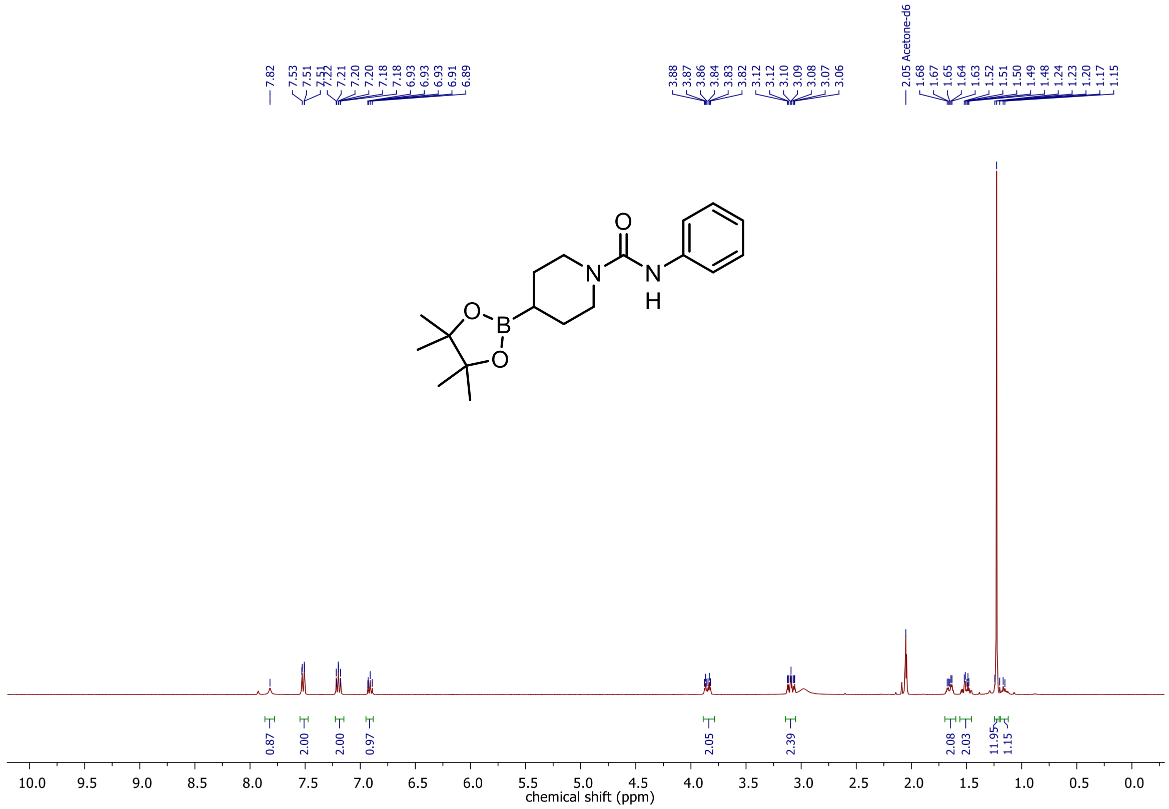

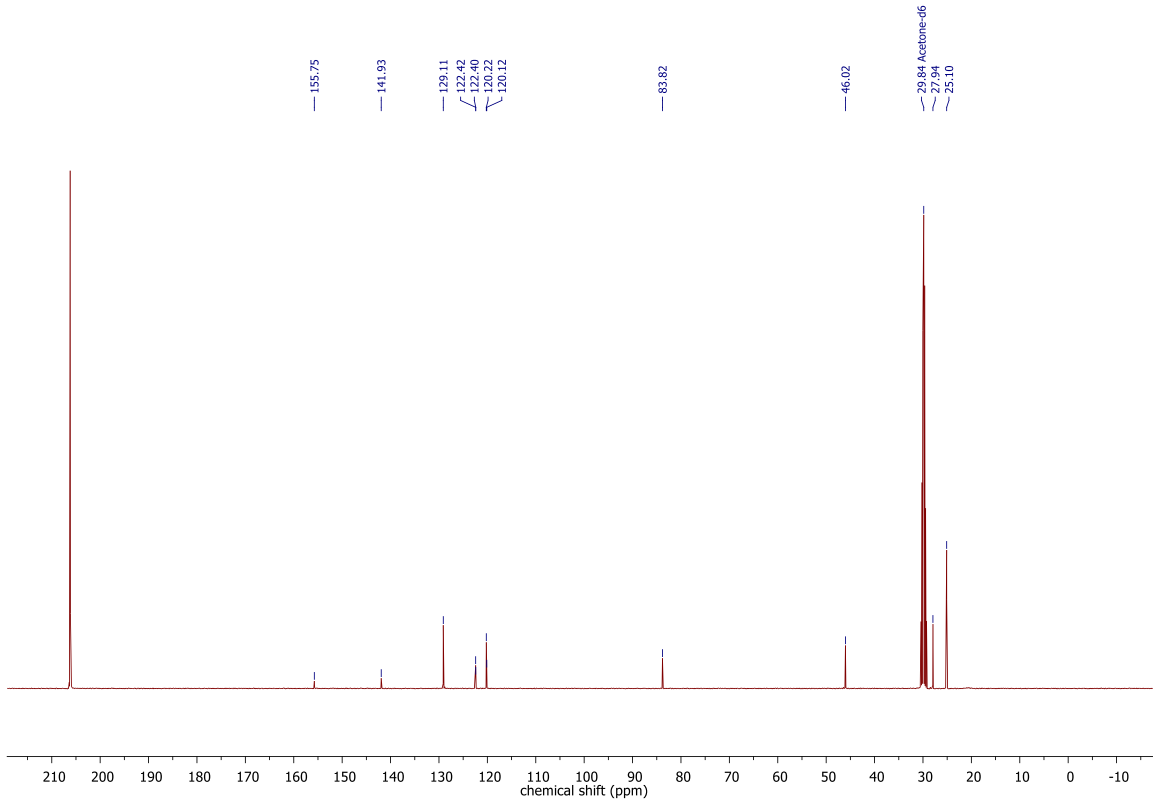

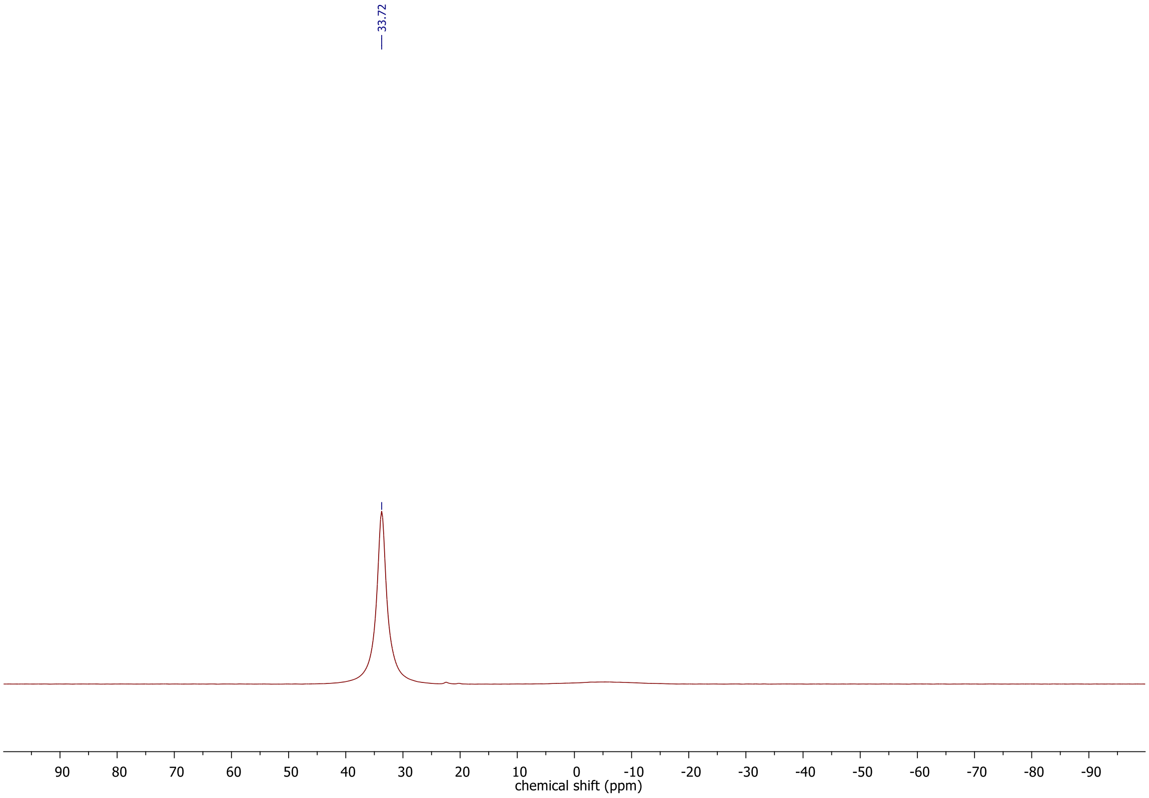
33.72
90
80
70
60
50
40
30
20
10
0
-10
-20
-30
-40
-50
-60
-70
-80
-90
chemical shift (ppm)

4-((2*E*,4*E*)-5-(Benzo[d][1,3]dioxol-5-yl)penta-2,4-dienoyl)-N-phenylpiperazine-1-carboxamide (7k)

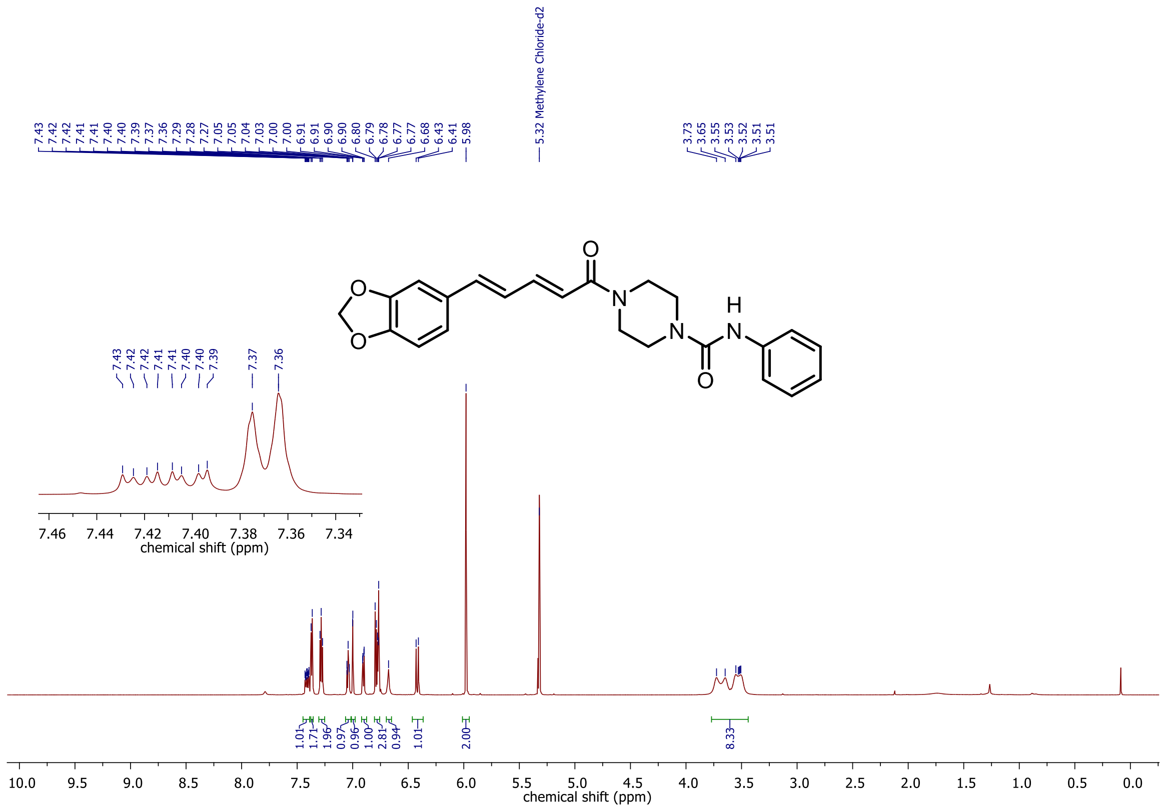

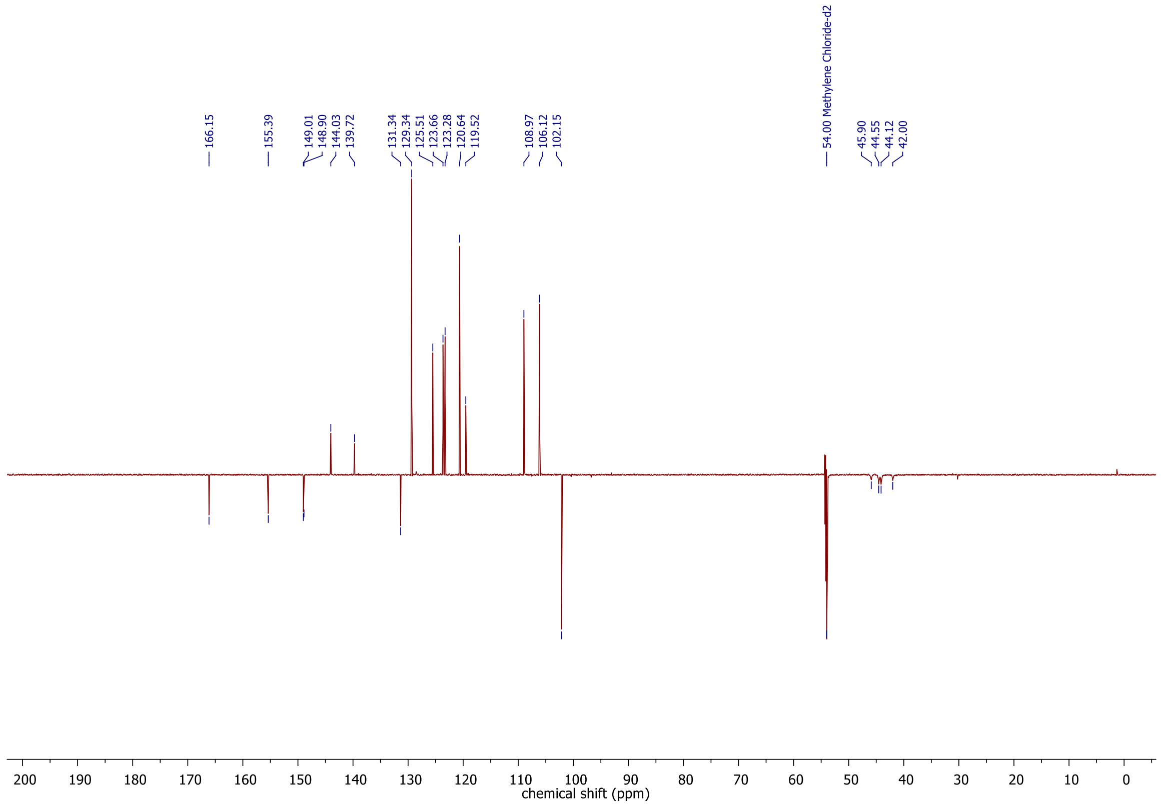

(*S*)-1-Methyl-1-(3-(naphthalen-1-yloxy)-3-(thiophen-2-yl)propyl)-3-phenylurea (7l)

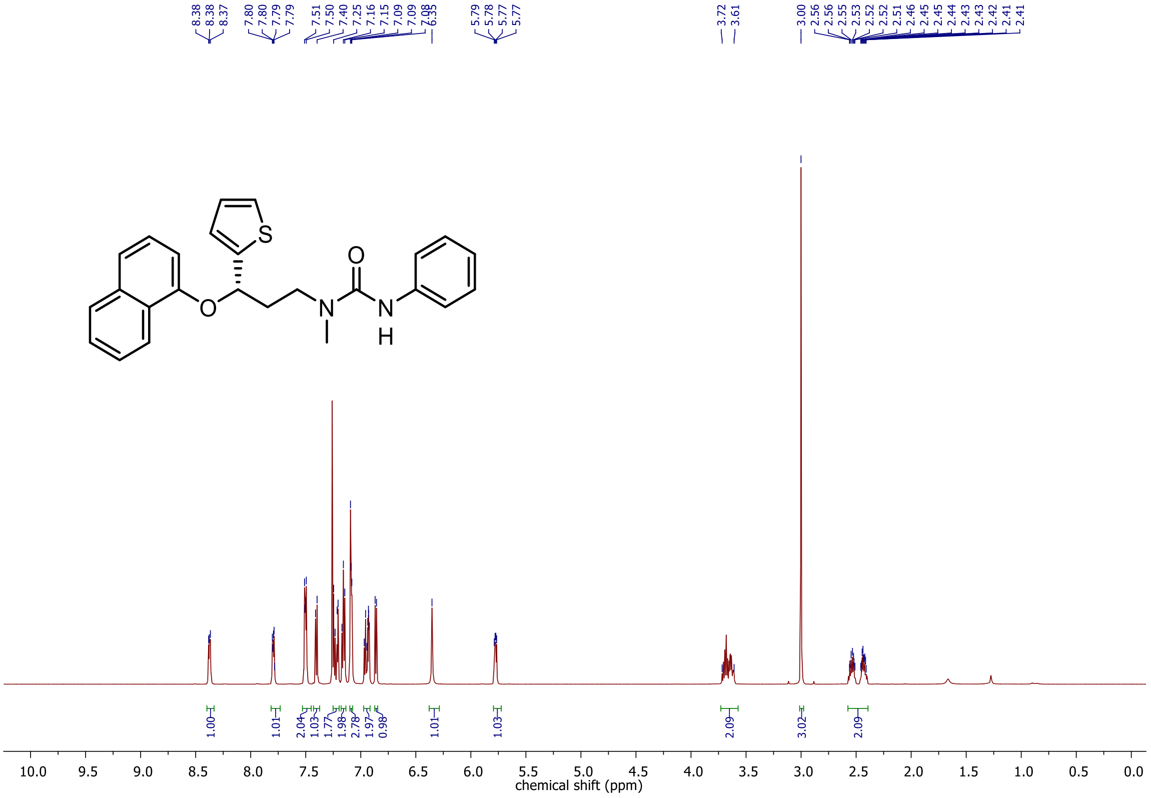

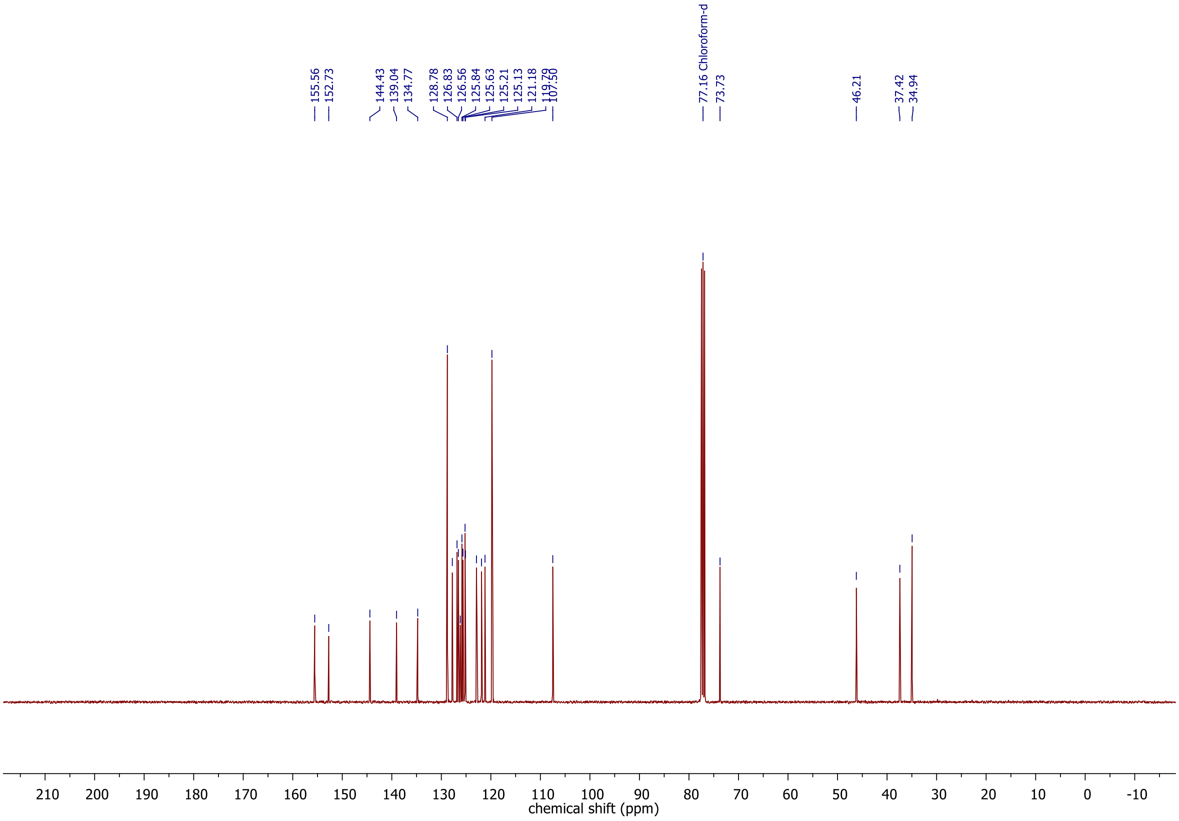

(3*S*,4*R*)-3-((Benzo[*d*][1,3]dioxol-5-yloxy)methyl)-4-(4-fluorophenyl)-*N*-phenylpiperidine-1-carboxamide (7m)

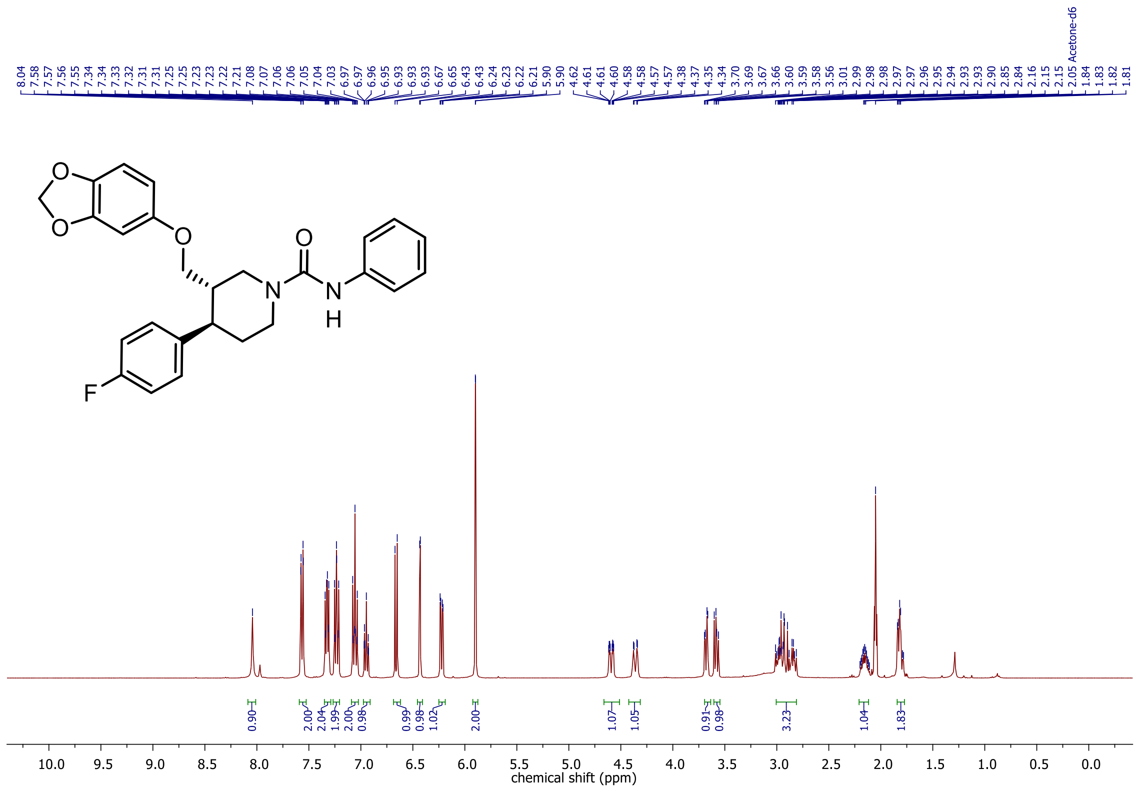

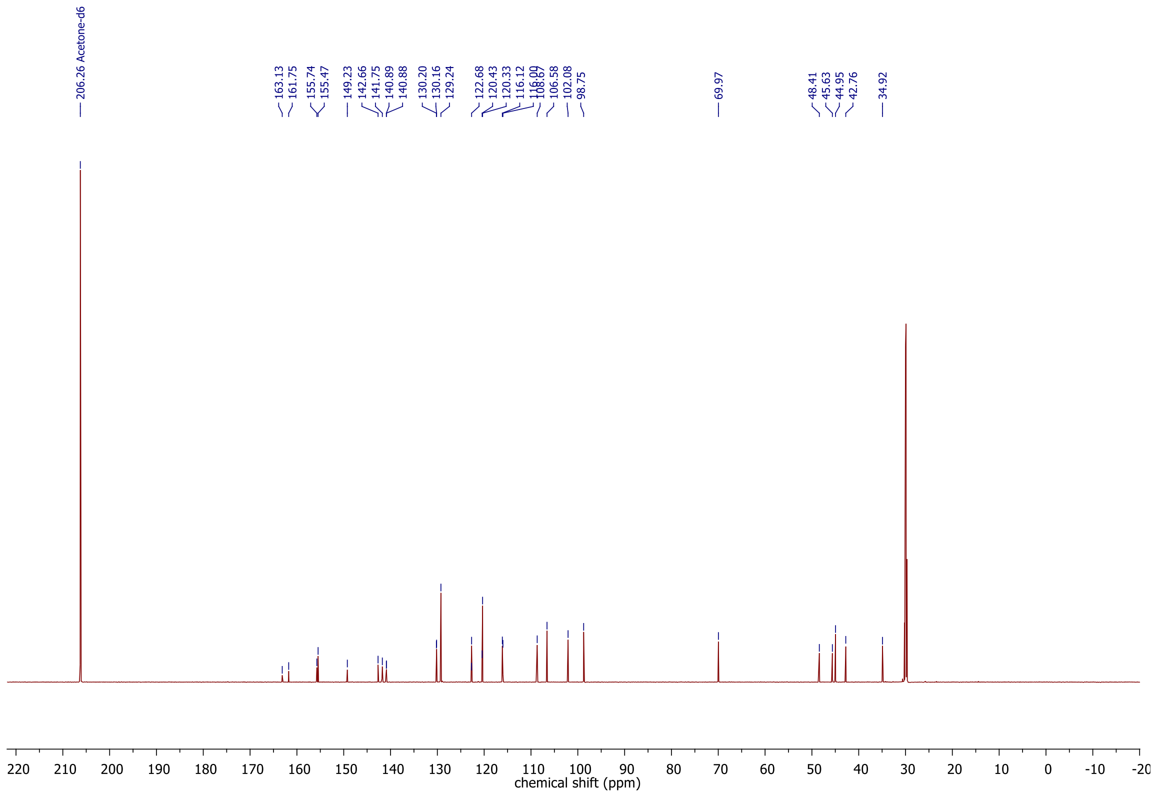

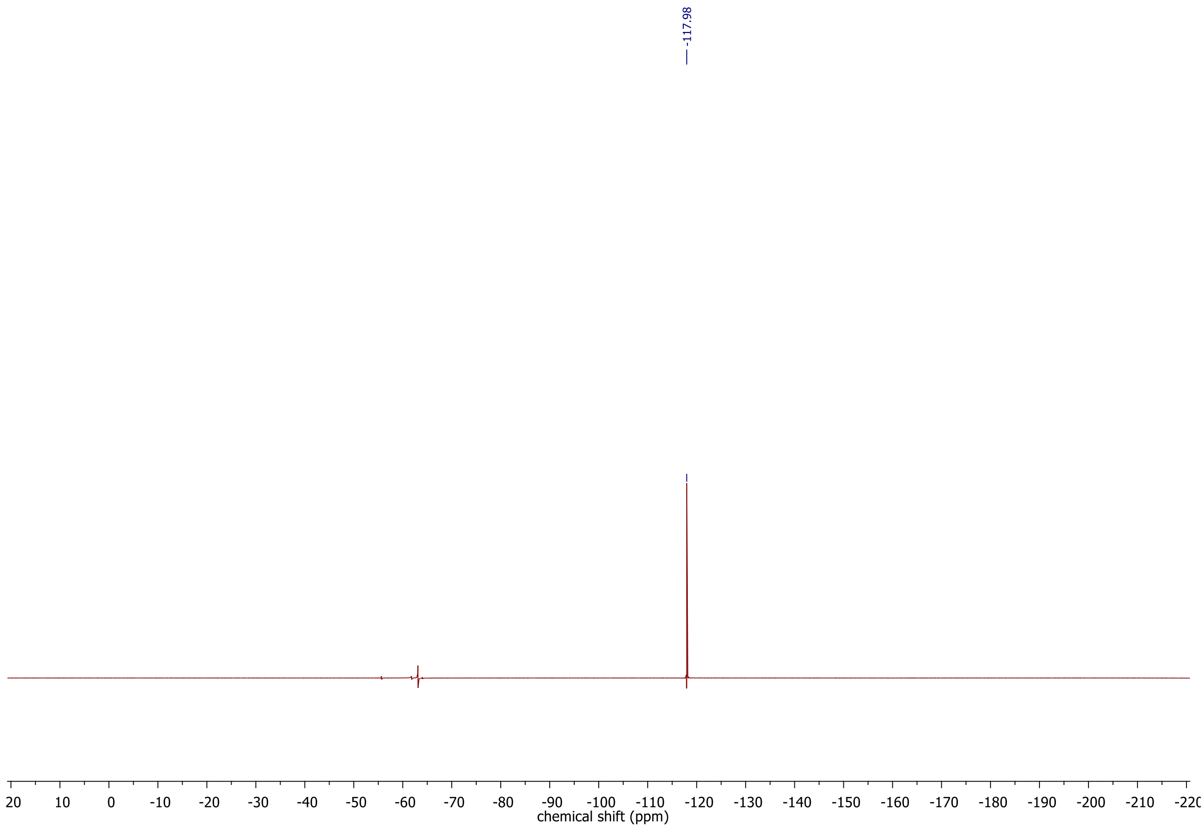
-117.98
20 10 0 -10 -20 -30 -40 -50 -60 -70 -80 -90 -100 -110 -120 -130 -140 -150 -160 -170 -180 -190 -200 -210 -220
chemical shift (ppm)

4-(8-Chloro-5,6-dihydro-11*H*-benzo[5,6]cyclohepta[1,2-*b*]pyridin-11-ylidene)-*N*-phenylpiperidine-1-carboxamide (7n)

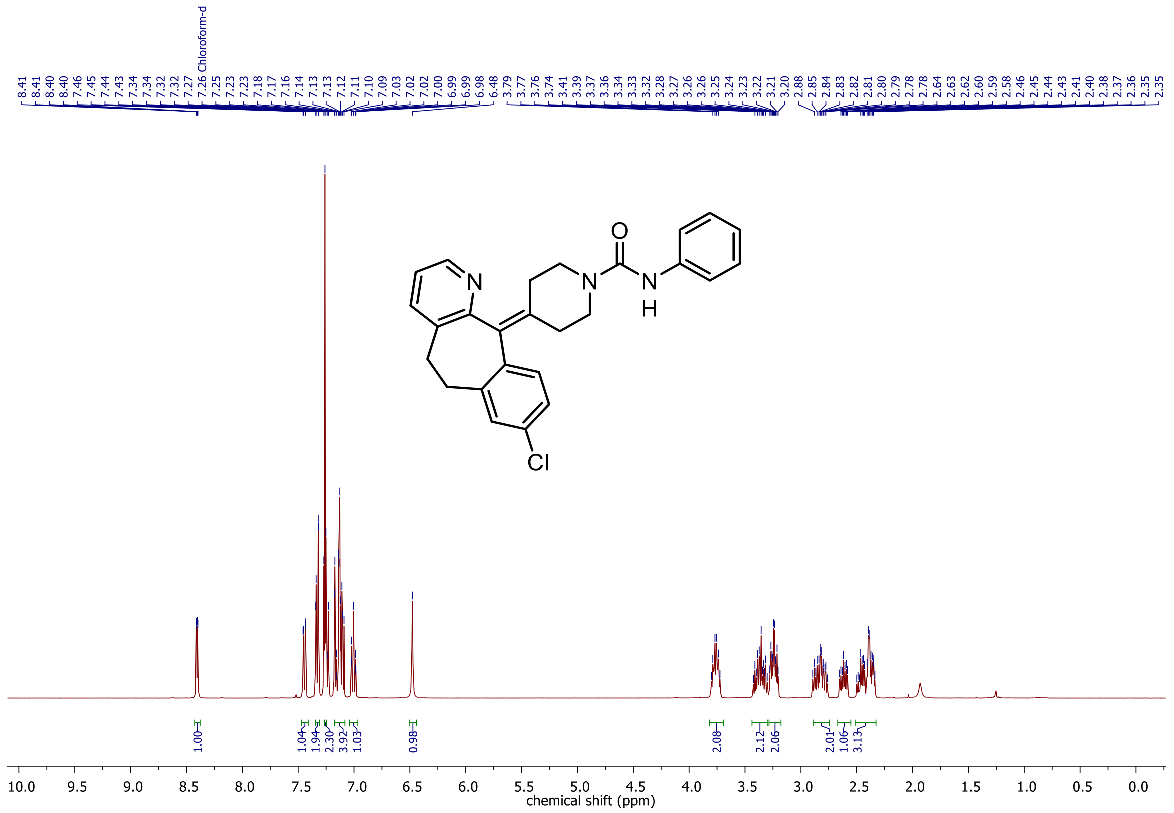

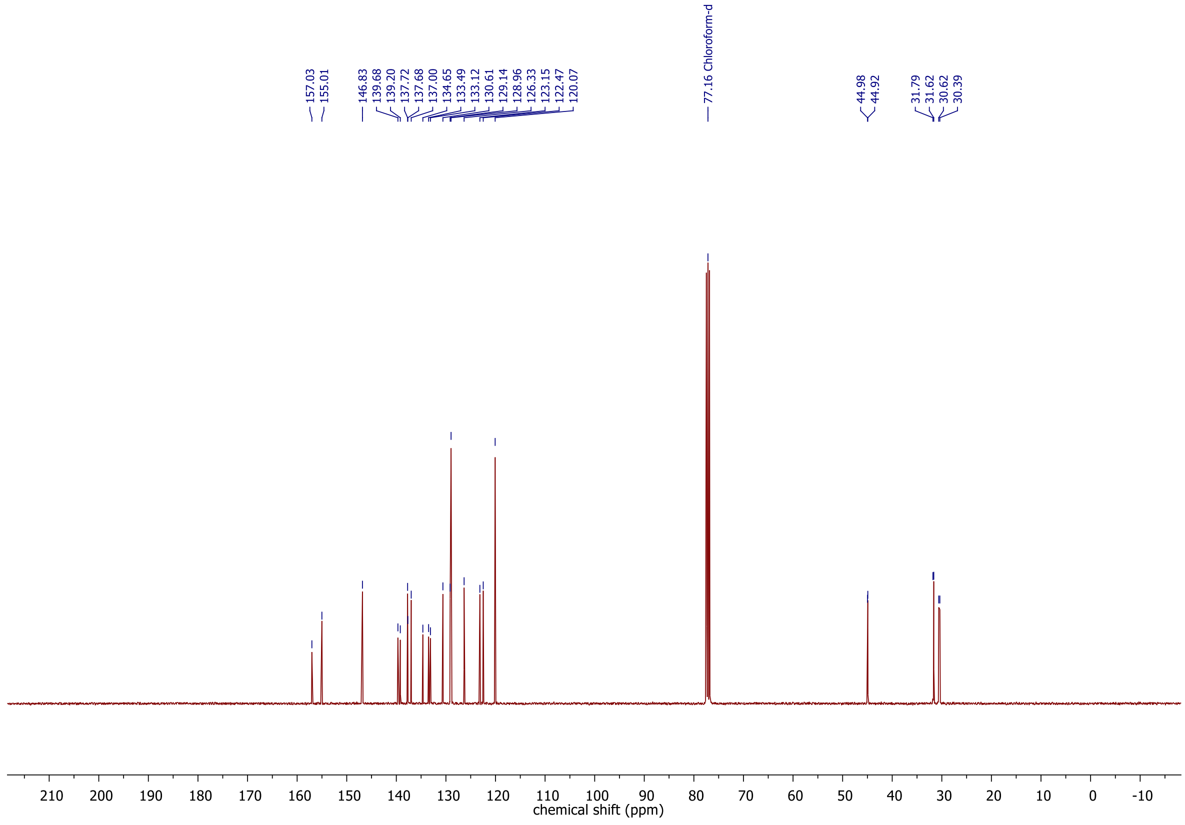

4-(2-Chlorodibenzo[*b*,*f*][1,4]oxazepin-11-yl)-*N*-phenylpiperazine-1-carboxamide (7o)

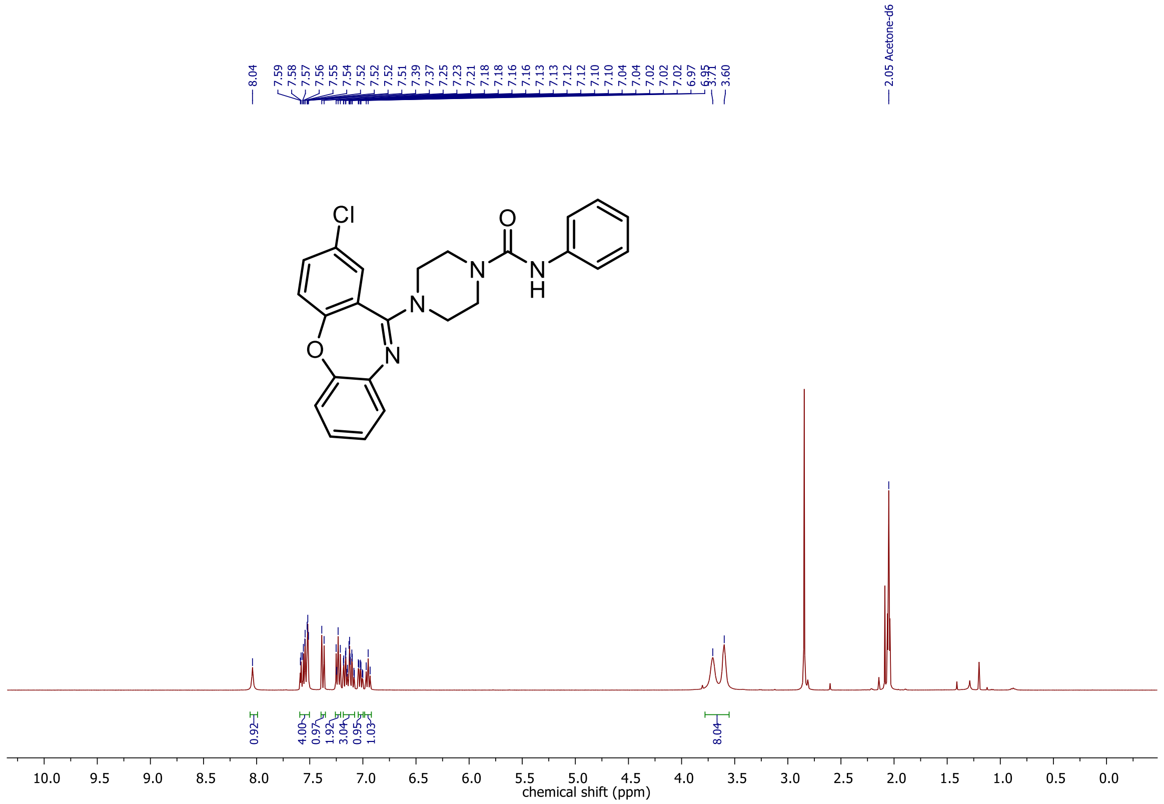

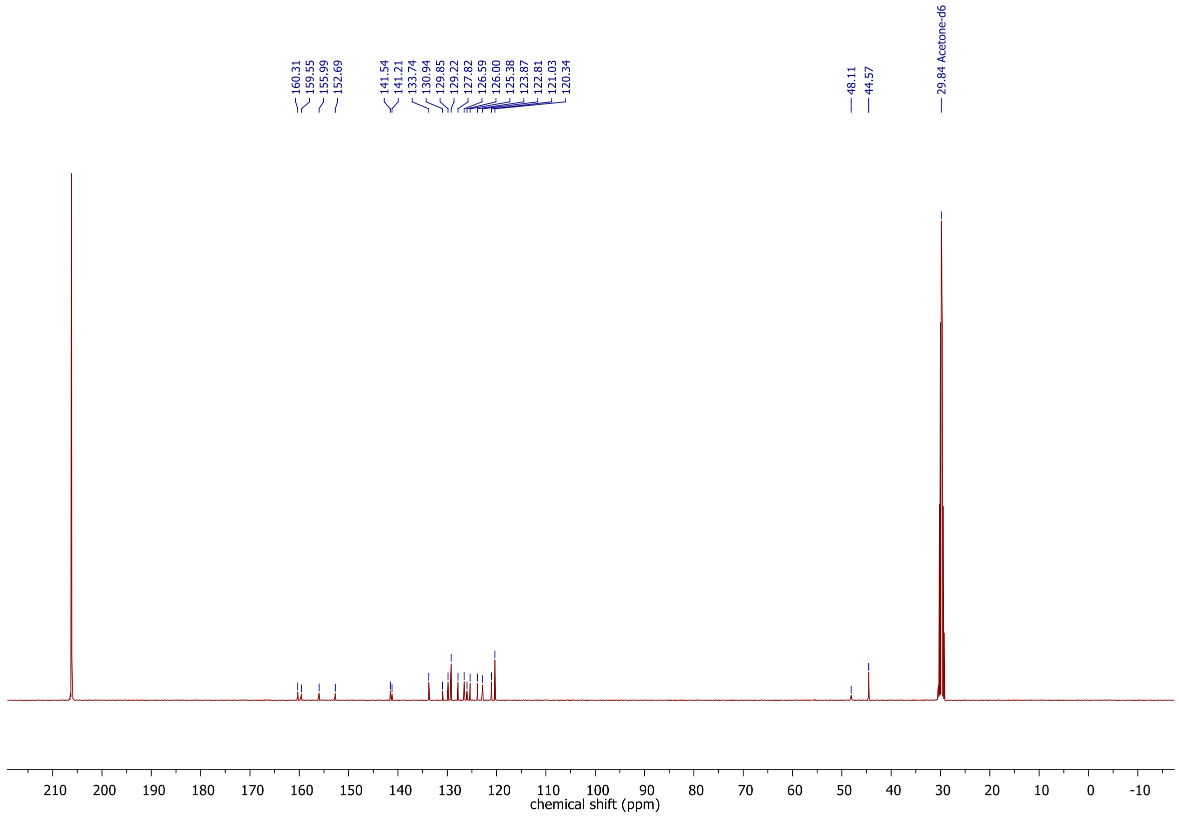

1-(2-(1*H*-Indol-3-yl)ethyl)-1-benzyl-3-phenylurea (9)

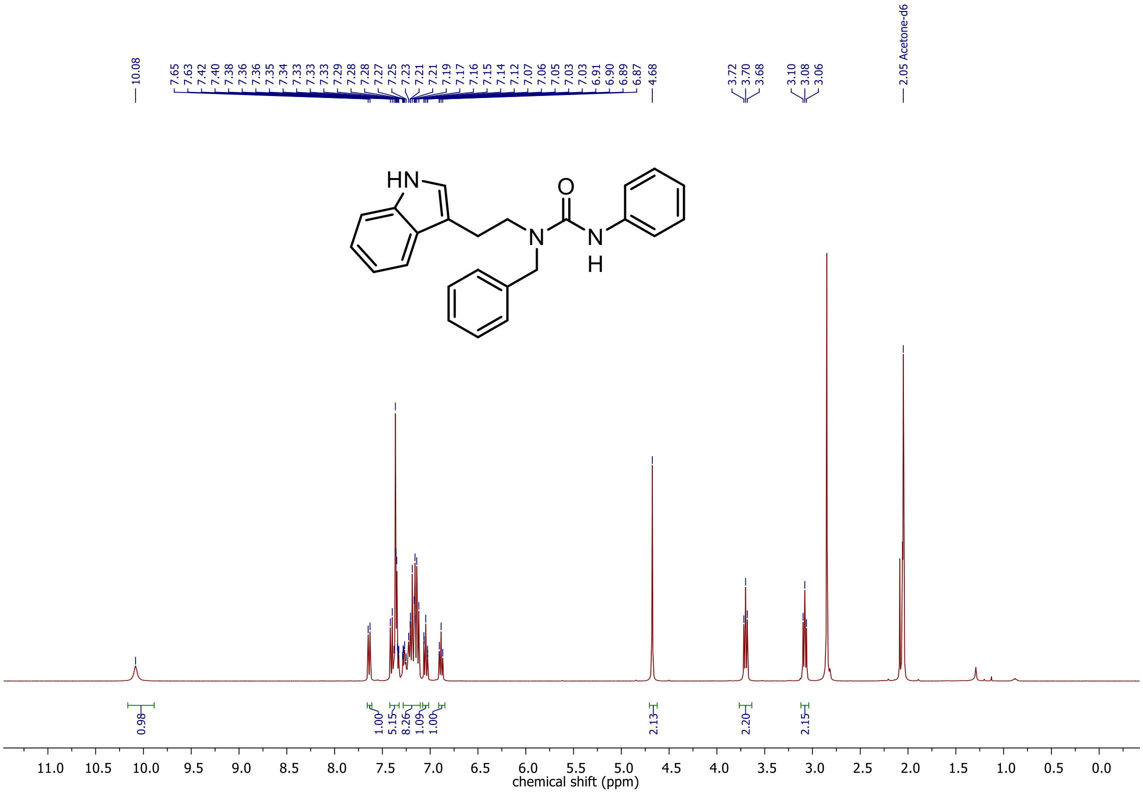

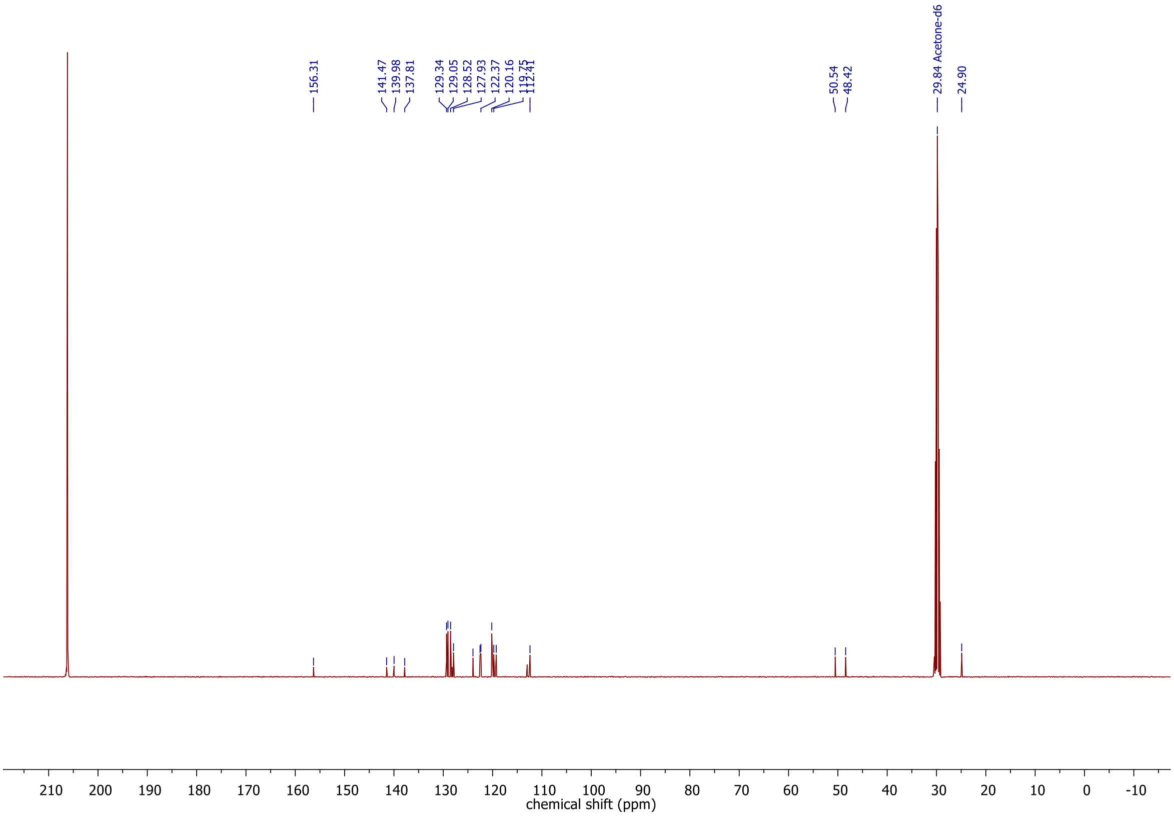

3-Oxo-*N*-(quinolin-2-yl)-8-azabicyclo[3.2.1]octane-8-carboxamide (10)

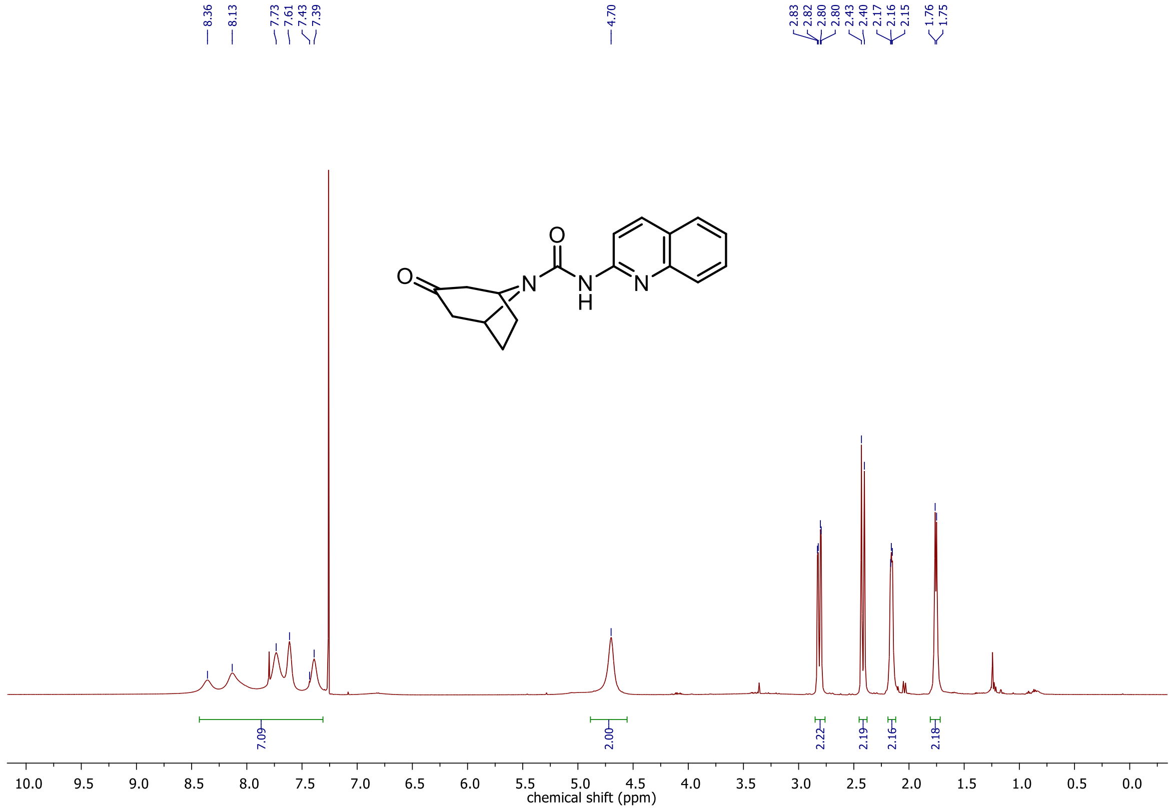

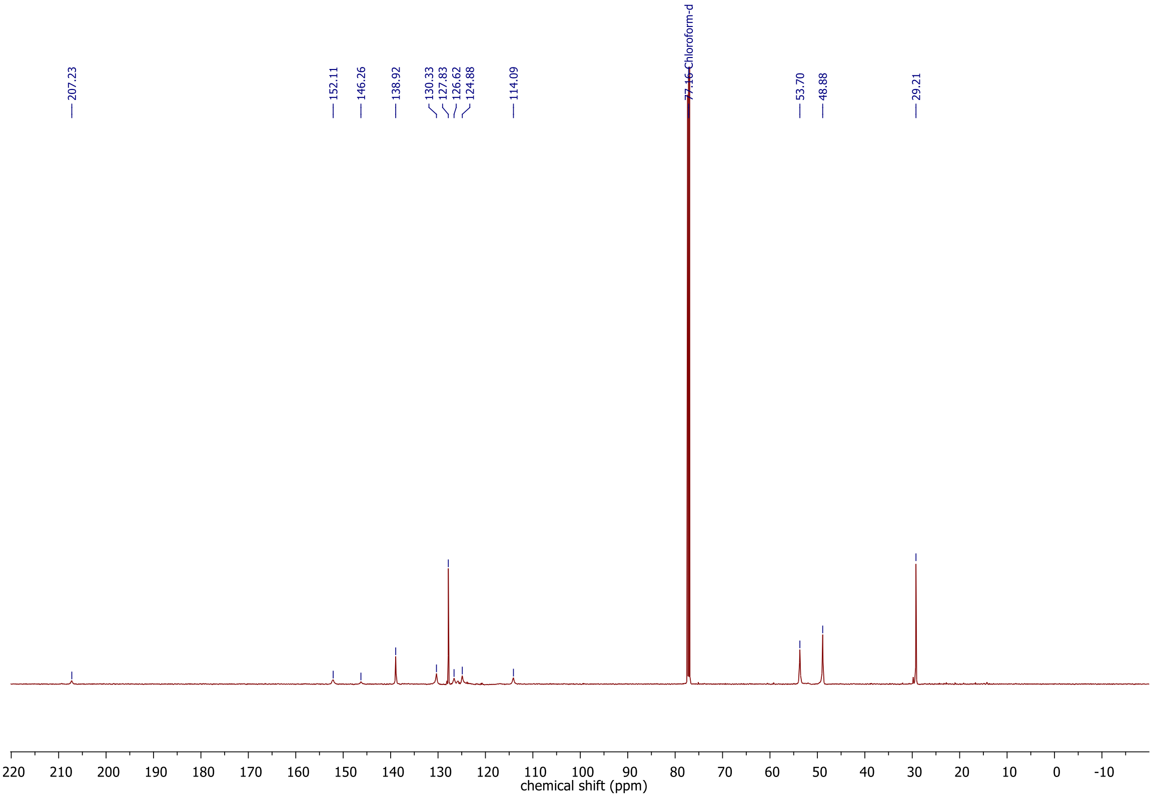

Methyl (((*S*)-1-((tert-butoxycarbonyl)amino)-2-phenylethyl)carbamoyl)-*L*-isoleucinate (12)

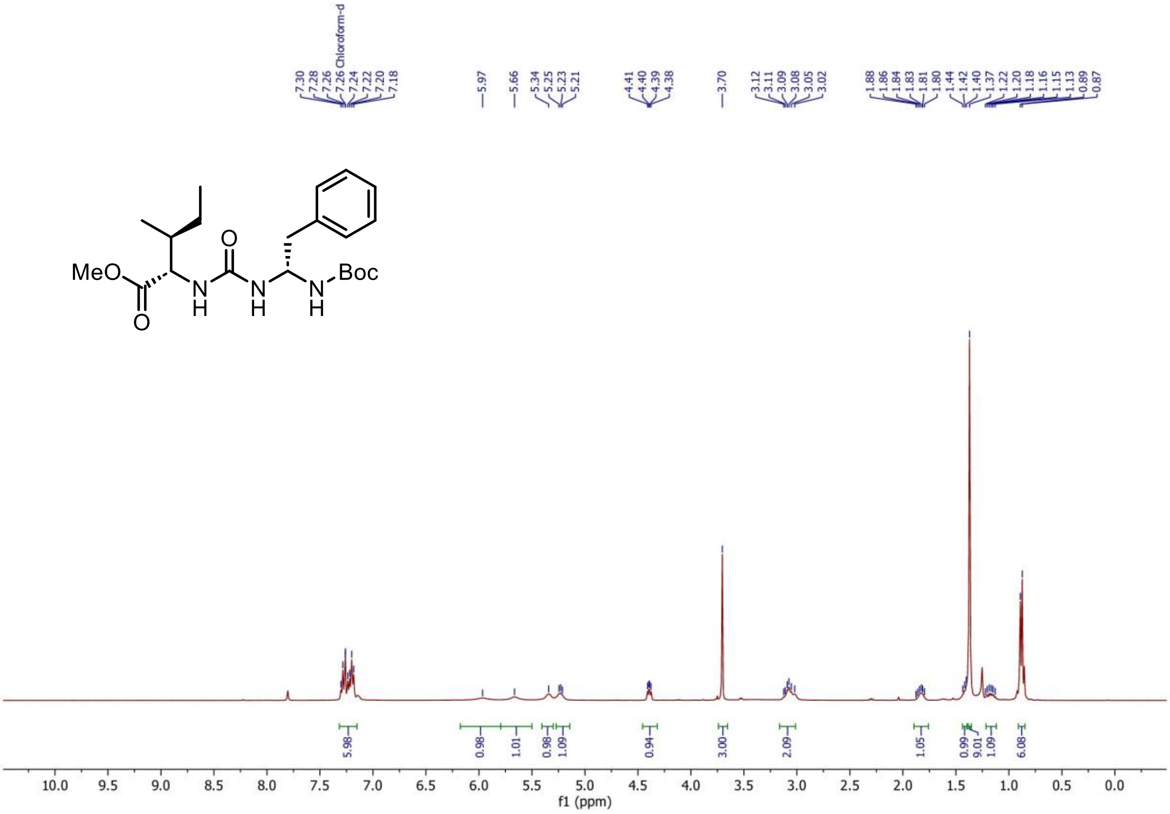

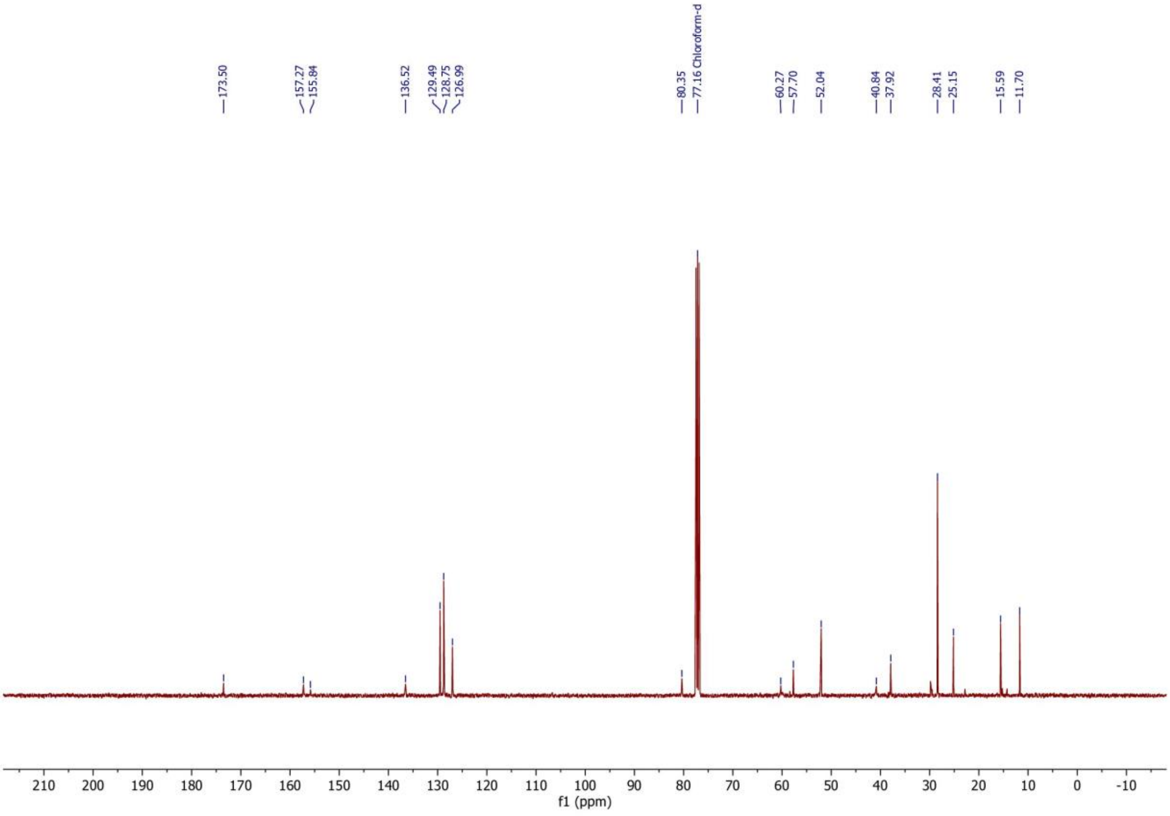

8. References

[1] Y. Kosugi, M. Akakura, K. Ishihara, *Tetrahedron* **2007**, *63*, 6191–6203.

[2] P. Spieß, A. Sirvent, I. Tiefenbrunner, J. Sargueil, A. J. Fernandes, A. Arroyo-Bondía, R. Meyrelles, D. Just, A. Prado-Roller, S. Shaaban, D. Kaiser, N. Maulide, *Chem. Eur. J.* **2023**, *29*, e202301312.

[3] P. Spieß, J. Brześkiewicz, R. Meyrelles, D. Just, N. Maulide, *Angew. Chem. Int. Ed.* **2024**, e202318304.

[4] G. Dettori, S. Gaspa, A. Porcheddu, L. De Luca, *Adv. Synth. Catal.* **2014**, *356*, 2709–2713.

[5] E. R. Trivedi, S. V. Eliseeva, J. Jankolovits, M. M. Olmstead, S. Petoud, V. L. Pecoraro, *J. Am. Chem. Soc.* **2014**, *136*, 1526–1534.
